# Structural Mechanism for Modulation of Synaptic Neuroligin-Neurexin Signaling by MDGA Proteins

**DOI:** 10.1016/j.neuron.2017.09.011

**Published:** 2017-09-27

**Authors:** Jonathan Elegheert, Vedrana Cvetkovska, Amber J. Clayton, Christina Heroven, Kristel M. Vennekens, Samuel N. Smukowski, Michael C. Regan, Wanyi Jia, Alexandra C. Smith, Hiro Furukawa, Jeffrey N. Savas, Joris de Wit, Jo Begbie, Ann Marie Craig, A. Radu Aricescu

(Neuron *95*, 896–913; August 16, 2017)

After publication, we noticed a number of minor errors within the main text and Figures 1 and 4 that escaped our attention during the proofreading of the manuscript.

In the top left panel in Figure 1C, FnIII_7_ loop *C’E* is incorrectly labeled as *CE*.

In the legend to Figure 3A, the buried surface area of Site II is incorrectly stated as 859 Å^2^, while the correct value is 1,000 Å^2^.

In Figure 4B, residue Glu294 (E294) is incorrectly labeled as Asp294 (D294). This error is also present in the main text paragraph “MDGA and NRX Share Binding Interfaces on NL.”

The errors have no effect on any of the conclusions in the paper, and the main text and Figures 1 and 4 have now been corrected online. The authors apologize for any confusion the errors may have caused.

**Figure 1C:**
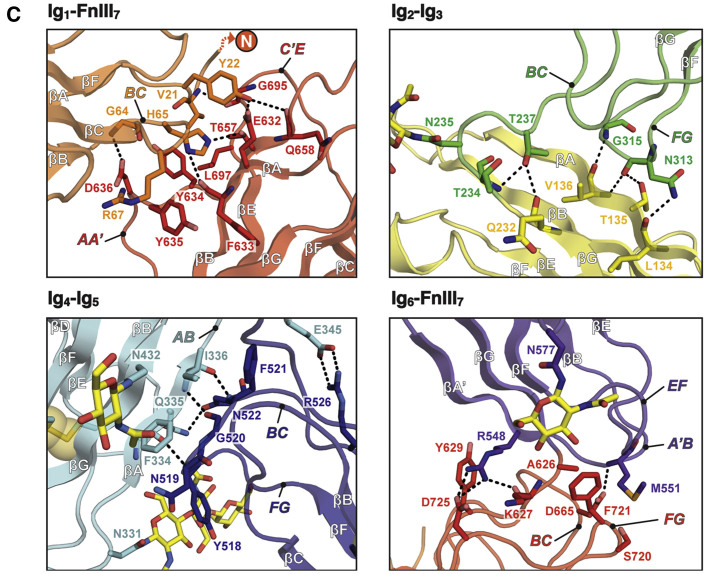
Crystal Structure of MDGA1 (corrected)

**Figure 1C:**
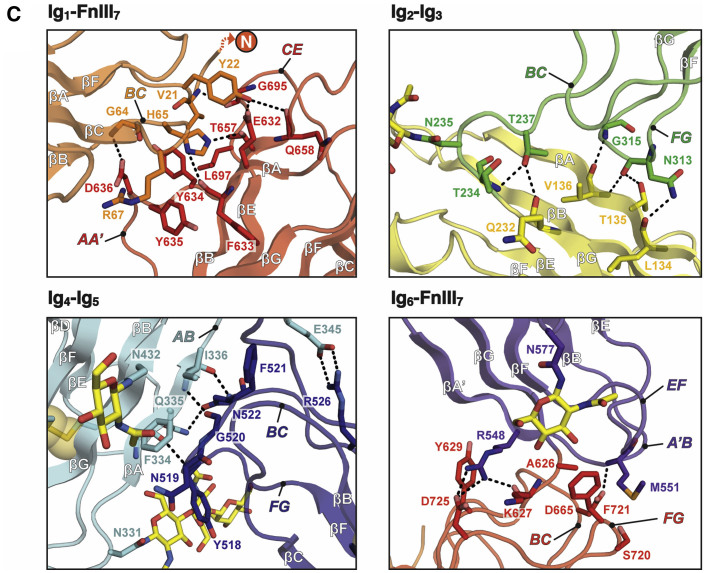
Crystal Structure of MDGA1 (original)

**Figure 4B:**
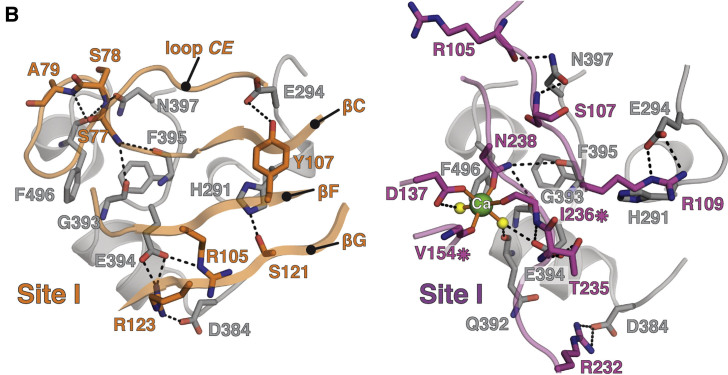
MDGA and NRX Compete for Binding to the NL Site I Interface (corrected)

**Figure 4B:**
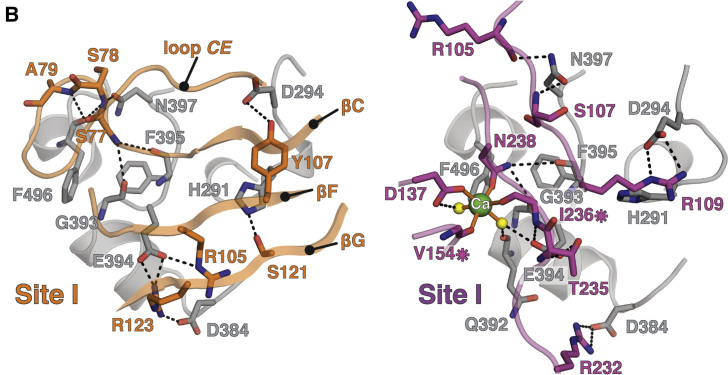
MDGA and NRX Compete for Binding to the NL Site I Interface (original)

