# Structural Mechanism for Modulation of Synaptic Neuroligin-Neurexin Signaling by MDGA Proteins

**DOI:** 10.1016/j.neuron.2020.12.006

**Published:** 2021-01-06

**Authors:** Jonathan Elegheert, Vedrana Cvetkovska, Amber J. Clayton, Christina Heroven, Kristel M. Vennekens, Samuel N. Smukowski, Michael C. Regan, Wanyi Jia, Alexandra C. Smith, Hiro Furukawa, Jeffrey N. Savas, Joris de Wit, Jo Begbie, Ann Marie Craig, A. Radu Aricescu

(Neuron *95*, 896–913; August 16, 2017)

It has come to our attention that there is an error in our report regarding the ASD-linked NL3 mutation Arg451Cys and its potential impact on the NL–MDGA interaction. It concerns the paragraph “The ASD-Linked NL3 Mutation Arg451Cys Prevents Suppression of Synapse Formation by MDGA1” and the associated Figures 7, S8B, S8C, and S8D. The ASD-linked Arg→Cys (R→C) mutation in human NL3 corresponds to Arg451 (sequence motif RKTLVA) in NL3 lacking spliced sequence A (SSA; UniProt entry Q9NZ94-2). However, we erroneously introduced the mutation based on the sequence numbering of NL3 containing the 20-residue spliced sequence A1 (UniProt entry Q9NZ94-1), which, by chance, also has an Arg at position 451 (sequence motif RETIKF). This latter mutation locates to the Site II interface and resembles the other ΔSite II mutants we described in Figure 6A. The correct R451C mutation, however, is not situated in either the Site I or Site II NL–MDGA interfaces. Therefore, our conclusion that R451C prevents NL–MDGA complex formation is wrong, and any speculation based on it in the Discussion section may be disregarded. We note that this error has no impact on the validity of any of the crystal structures or other results.

Furthermore, we noticed two unrelated minor errors within Figures 2, 4, and S3.

In Figures 2A and S3A, a N-linked glycosylation site is incorrectly shown in the intracellular (IC) tail of NL1.

**Figure 2A:**
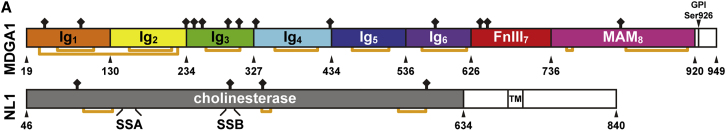
Crystal Structure of an NL-MDGA Complex

**Figure S3A:**
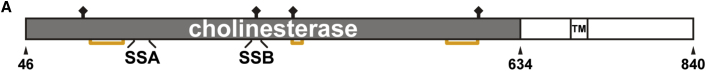
Sequence Alignment of the NL1, -2, -3, -4 and -5 Cholinesterase Domains, Related to Figure 2

In Figure 4C, there is a sign error in the bottom left panel: it reads −TΔS = −1.02 kcal mol^−1^ instead of 1.02 kcal mol^−1^. Therefore, ΔG = −8.66 kcal mol^−1^ instead of −6.62 kcal mol^−1^. The value for the K_D_ is correct, however.

**Figure 4C:**
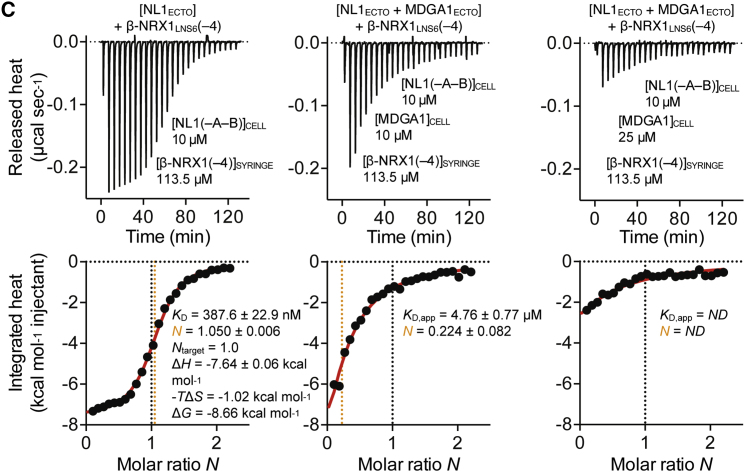
MDGA and NRX Compete for Binding to the NL Site I Interface

The authors apologize for any confusion the errors may have caused.

