# Supplementary material for: Structural Mechanism for Modulation of Synaptic Neuroligin-Neurexin Signaling by MDGA Proteins
Source: Neuron. 2017 Aug 16;95(4):896–913.e10. doi: 10.1016/j.neuron.2017.07.040 (PMC5563082; doi:10.1016/j.neuron.2017.07.040)
Supplement: Document S1. Figures S1–S11 and Tables S1 and S3–S5 [file mmc1.pdf]

Neuron, Volume *95*

Supplemental Information

Structural Mechanism for Modulation of Synaptic Neuroligin-Neurexin Signaling by MDGA Proteins

Jonathan Elegheert, Vedrana Cvetkovska, Amber J. Clayton, Christina Heroven, Kristel M. Vennekens, Samuel N. Smukowski, Michael C. Regan, Wanyi Jia, Alexandra C. Smith, Hiro Furukawa, Jeffrey N. Savas, Joris de Wit, Jo Begbie, Ann Marie Craig, and A. Radu Aricescu

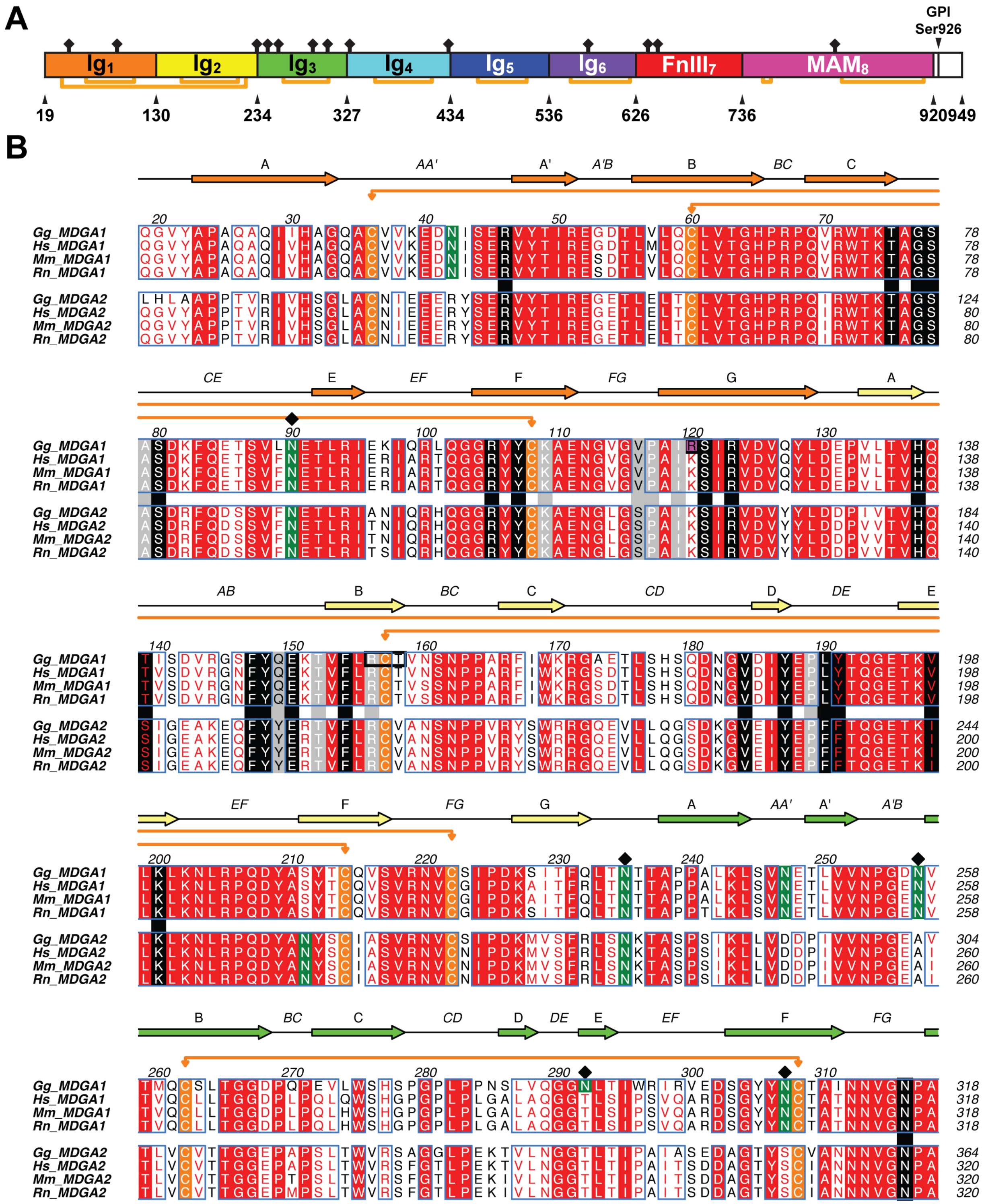
A
GPI
Ser926
Ig1
Ig2
Ig3
Ig4
Ig5
Ig6
FnIII7
MAM8
19
130
234
327
434
536
626
736
920
949
B
Gg_MDGA1
Hs_MDGA1
Mm_MDGA1
Rn_MDGA1
Gg_MDGA2
Hs_MDGA2
Mm_MDGA2
Rn_MDGA2

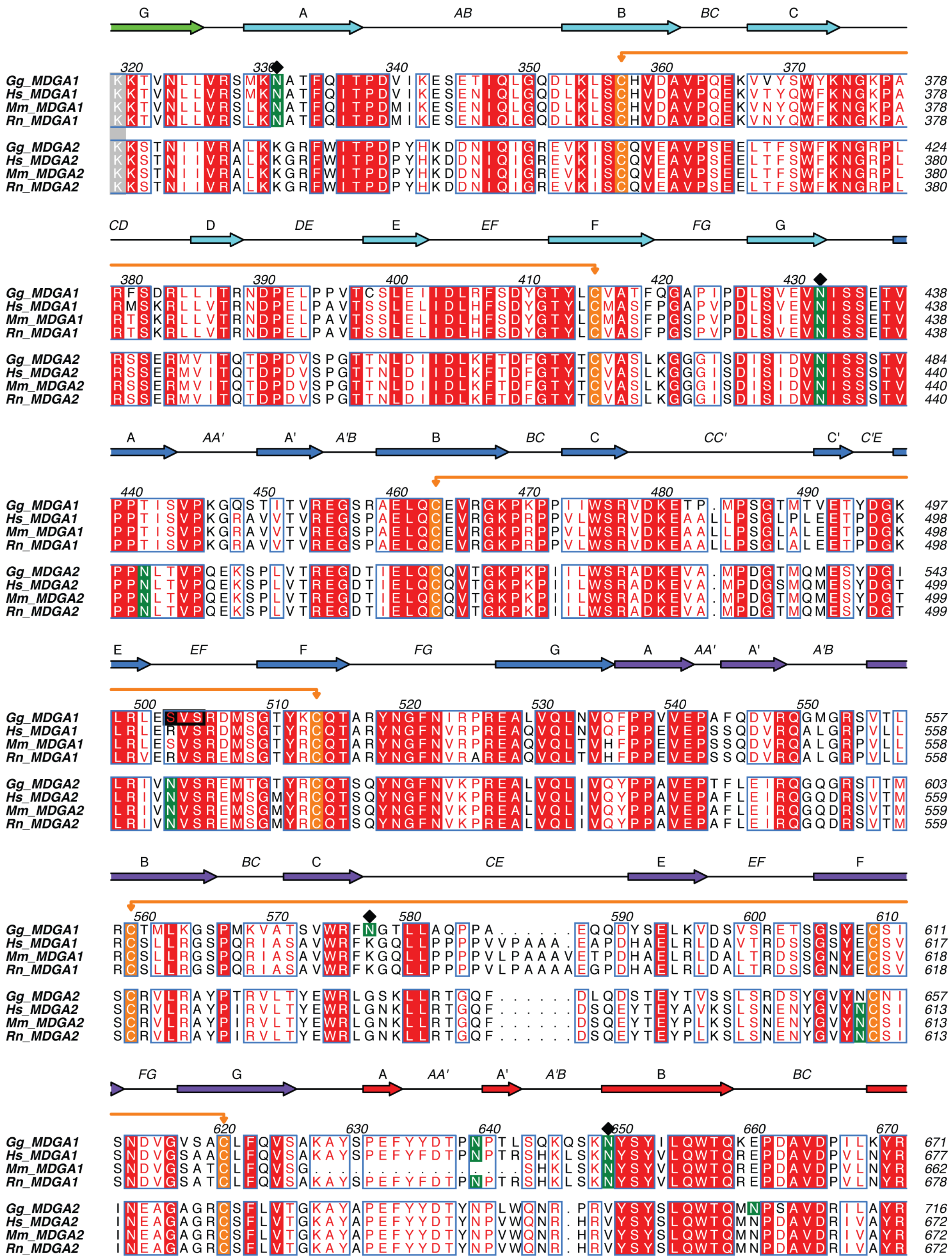
Gg_MDGA1
Hs_MDGA1
Mm_MDGA1
Rn_MDGA1
Gg_MDGA2
Hs_MDGA2
Mm_MDGA2
Rn_MDGA2
G A AB B BC C
CD D DE E EF F FG G
A AA' A' A'B B BC C CC' C' C'E
E EF F FG G A AA' A' A'B
B BC C CE E EF F
FG G A AA' A' A'B B BC

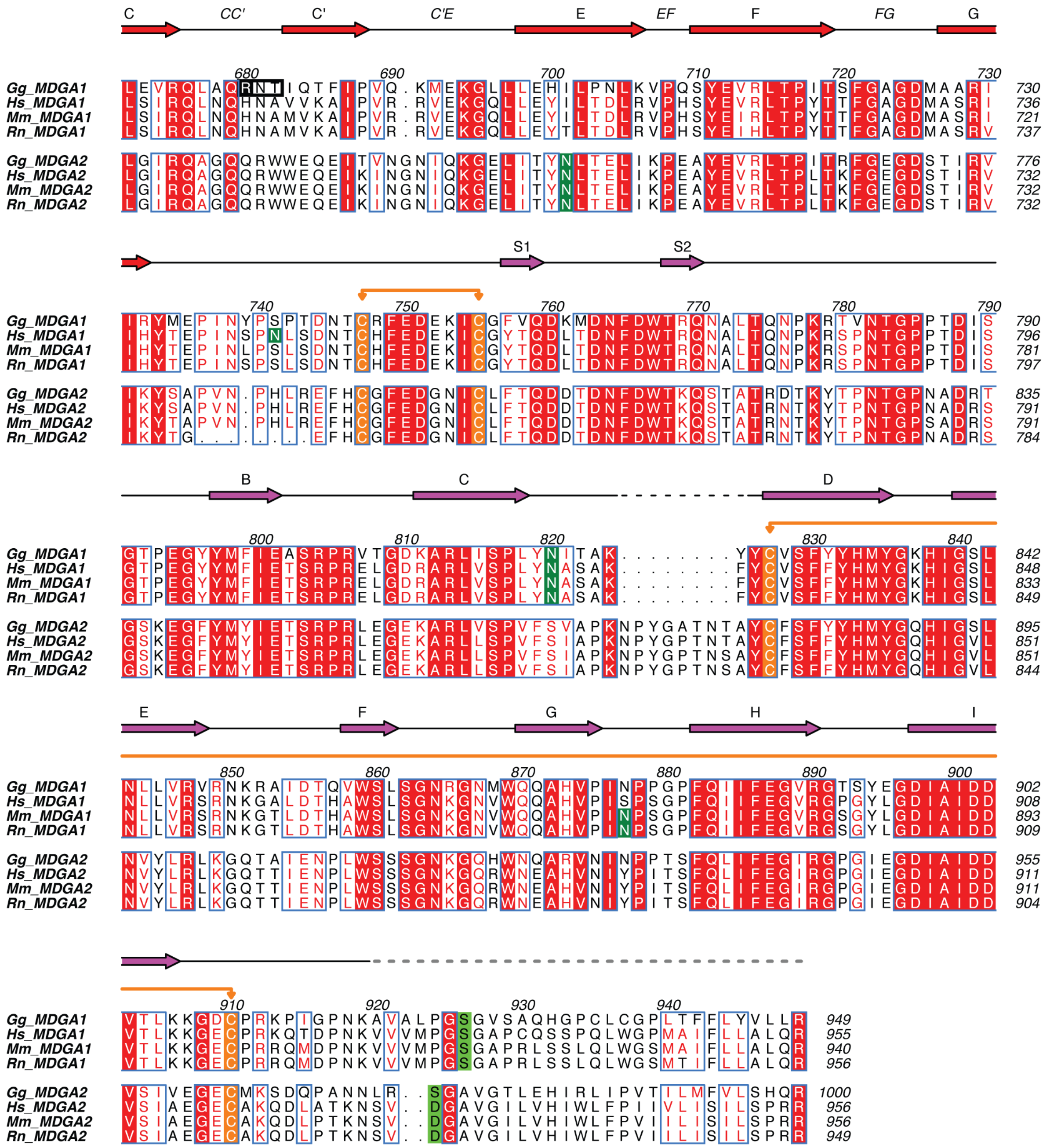

Figure S1. Sequence Alignment of the MDGA1 and -2 Ectodomains. Related to Figure 1 and Figure 2.

(A) Schematic representation of the chicken MDGA1 domain structure. Black diamonds denote predicted N-linked glycosylation sites.

(B) Amino acid sequences are taken from the UniProt database (entry code); *Gg*_MDGA1 (Q0WYX8), *Hs*_MDGA1 (Q8NFP4), *Mm*_MDGA1 (Q0PMG2), *Rn*_MDGA1 (P85171), *Gg*_MDGA2 (F1NIA0), *Hs*_MDGA2 (Q7Z553), *Mm*_MDGA2 (P60755), *Rn*_MDGA2

(P60756). *Gg*; *Gallus gallus*, *Hs*; *Homo sapiens*, *Mm*; *Mus Musculus*, *Rn*; *Rattus norvegicus*. Secondary structure elements for *Gg*_MDGA1 are annotated above the alignment. MDGA1 cysteine residues participating in disulfide bonds are shaded in orange and connected by orange lines. Putative N-linked glycosylation sites for all MDGA members are shaded in dark green. Black diamonds further highlight crystallographically confirmed N-linked MDGA1 glycosylation sites. The predicted residue positions where amide linking of the GPI anchor occurs (Ser932 in *Hs*_MDGA1 and Asp931 in *Hs*_MDGA2) are shaded in green. Positions where N-linked glycan consensus sequences (Asn-X-Ser/Thr) were inserted at cMDGA1$_{ECTO}$ crystallographic homophilic interfaces (Arg156Asn in Ig$_2$, Ser502Asn in Ig$_5$, and Arg680Asn in FnIII$_7$) are boxed in black. The Arg120Lys (R120K) mutation, introduced to bring the chicken MDGA1 sequence in line with the human, rat and mouse MDGA1-2 sequences at that position, is shaded in magenta and boxed in black. MDGA Ig$_1$, Ig$_2$, and Ig$_3$ residues in the "core" and at the "rim" of the NL–MDGA interface are highlighted in black and grey vertical rectangles, respectively. Ig$_1$ is an I-set Ig superfamily (IgSF) domain, however it lacks strand C' and D (AA'BCEFG). Ig$_2$ is a canonical C1-set IgSF domain (ABCDEFG). Ig$_3$ is an I-set IgSF domain, however it lacks strand C' (AA'BCDEFG). Ig$_4$ is a canonical C1-set IgSF domain (ABCDEFG). Ig$_5$ is an I-set IgSF domain, however it lacks strand D (AA'BCC'EFG). Ig$_6$ is an I-set IgSF domain, however it lacks strand C' and D (AA'BCEFG), similarly to Ig$_1$. Additionally, there is no canonical disulfide bond between strand B (Cys559) and strand F (Cys609) in Ig$_6$. Instead, Cys559 pairs with Cys620 on strand G. Finally, FnIII$_7$ is C2-set, and strand A is divided into strand A and A', separated by a long loop (AA'BCC'EFG).

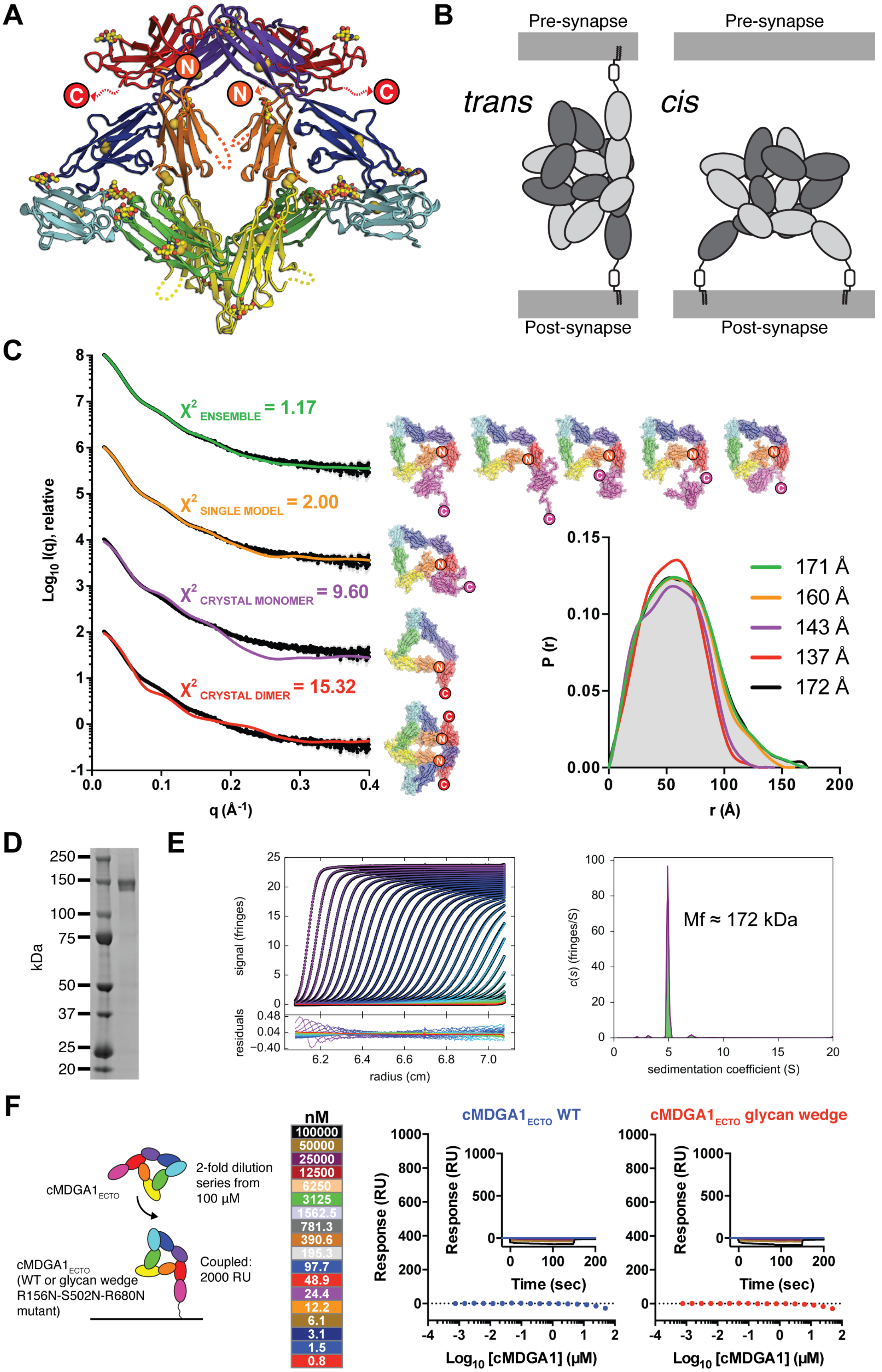

A
B
Pre-synapse
Pre-synapse
trans
cis
Post-synapse
Post-synapse
C
χ2 ENSEMBLE = 1.17
χ2 SINGLE MODEL = 2.00
χ2 CRYSTAL MONOMER = 9.60
χ2 CRYSTAL DIMER = 15.32
Log10 I(q), relative
q (Å-1)
P (r)
r (Å)
171 Å
160 Å
143 Å
137 Å
172 Å
D
kDa
E
signal (fringes)
residuals
radius (cm)
c(s) (fringes/S)
Mf ≈ 172 kDa
sedimentation coefficient (S)
F
cMDGA1ECTO
2-fold dilution series from 100 µM
cMDGA1ECTO (WT or glycan wedge R156N-S502N-R680N mutant)
Coupled: 2000 RU
nM
cMDGA1ECTO WT
cMDGA1ECTO glycan wedge
Response (RU)
Time (sec)
Log10 [cMDGA1] (µM)

Figure S2. MDGA1 is Monomeric in Solution. Related to Figure 1.

(A) Cartoon representation of the intertwined crystallographic $cMDGA1_{ECTO}$ dimer.

(B) Schematic representation of the potential MDGA1 homophilic *trans*-dimer or *cis*-dimer, GPI-anchored to the pre- and/or postsynaptic membranes.

(C) Solution structure of $cMDGA1_{ECTO}$. Experimental scattering curves (black) and calculated scattering patterns (colored) are shown to a maximal momentum transfer of $q = 0.40$ Å^{-1}. Individual data:fit pairs are displaced along an arbitrary y axis to allow for better visualization. Bottom curve: cMDGA1 crystallographic dimer (red). Second curve from bottom: extracted $cMDGA1_{ECTO}$ crystallographic monomer (purple). Second curve from top: best single monomeric model (orange). Top curve: best five-membered minimal ensemble (green). Atomic models, corresponding to the individual curves, are shown in ribbon and surface representation. The inset shows the experimental (black line and grey shade) and calculated (colored) pairwise distance distribution (P(r)) functions and derived maximum intra-particle distance (D_{MAX}) values.

(D) SDS-PAGE of glycosylated human $MDGA1_{ECTO}$ ($hMDGA1_{ECTO}$), showing an apparent molecular weight of ~150 kDa.

(E) SV-AUC data for $hMDGA1_{ECTO}$ at 60 μM. The sedimentation coefficient is ~5.1 S, and there is no indication of oligomer formation. The derived molar mass is consistent with a glycosylated, monomeric $hMDGA1_{ECTO}$ molecule.

(F) Lack of detectable $cMDGA1_{ECTO}$ self-interaction. Schematic representation of the structure-guided $cMDGA1_{ECTO}$ glycan wedge mutant ($cMDGA1_{ECTO}$-GW; combined Arg156Asn, Ser502Asn and Arg680Asn mutations); these mutations were chosen to disrupt crystallographically observed homophilic interfaces. Binding isotherms and sensorgrams for the (self-)interaction of wild-type $cMDGA1_{ECTO}$ and $cMDGA1_{ECTO}$-GW with wild-type $cMDGA1_{ECTO}$ are shown**.** $cMDGA1_{ECTO}$-GW serves as negative control for the detection of a potential $cMDGA1_{ECTO}$–$cMDGA1_{ECTO}$ self-interaction.

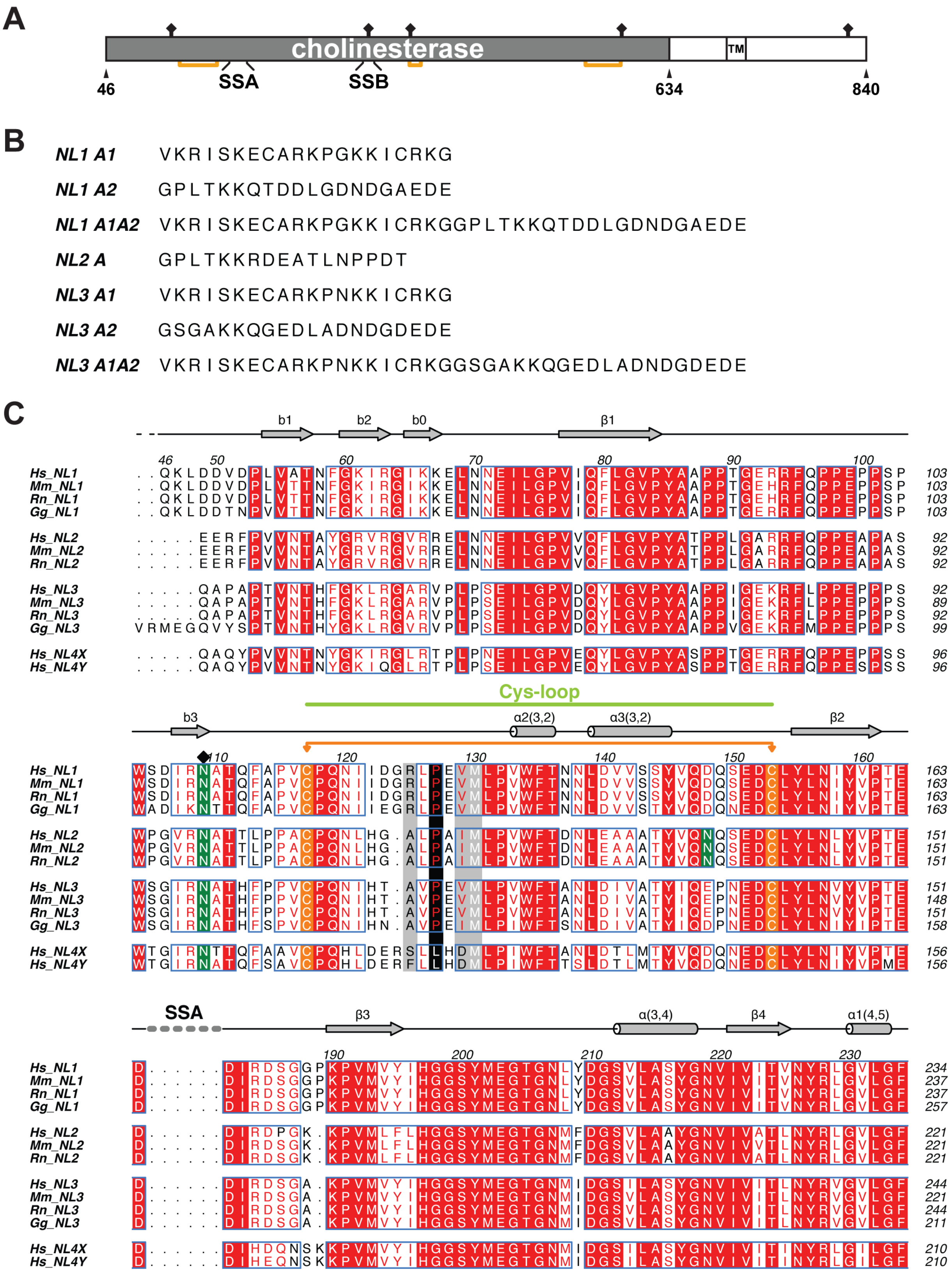
A
cholinesterase
TM
SSA
SSB
46
634
840
B
NL1 A1 VKRISKECARKPGKKICRKG
NL1 A2 GPLTKKQTDDLGDNDGAEDE
NL1 A1A2 VKRISKECARKPGKKICRKGGPLTKKQTDDLGDNDGAEDE
NL2 A GPLTKKRDEATLNPPDT
NL3 A1 VKRISKECARKPNKKICRKG
NL3 A2 GSGAKKQGEDLADNDGDEDE
NL3 A1A2 VKRISKECARKPNKKICRKGGSGAKKQGEDLADNDGDEDE
C
Cys-loop
SSA
Hs_NL1
Mm_NL1
Rn_NL1
Gg_NL1
Hs_NL2
Mm_NL2
Rn_NL2
Hs_NL3
Mm_NL3
Rn_NL3
Gg_NL3
Hs_NL4X
Hs_NL4Y

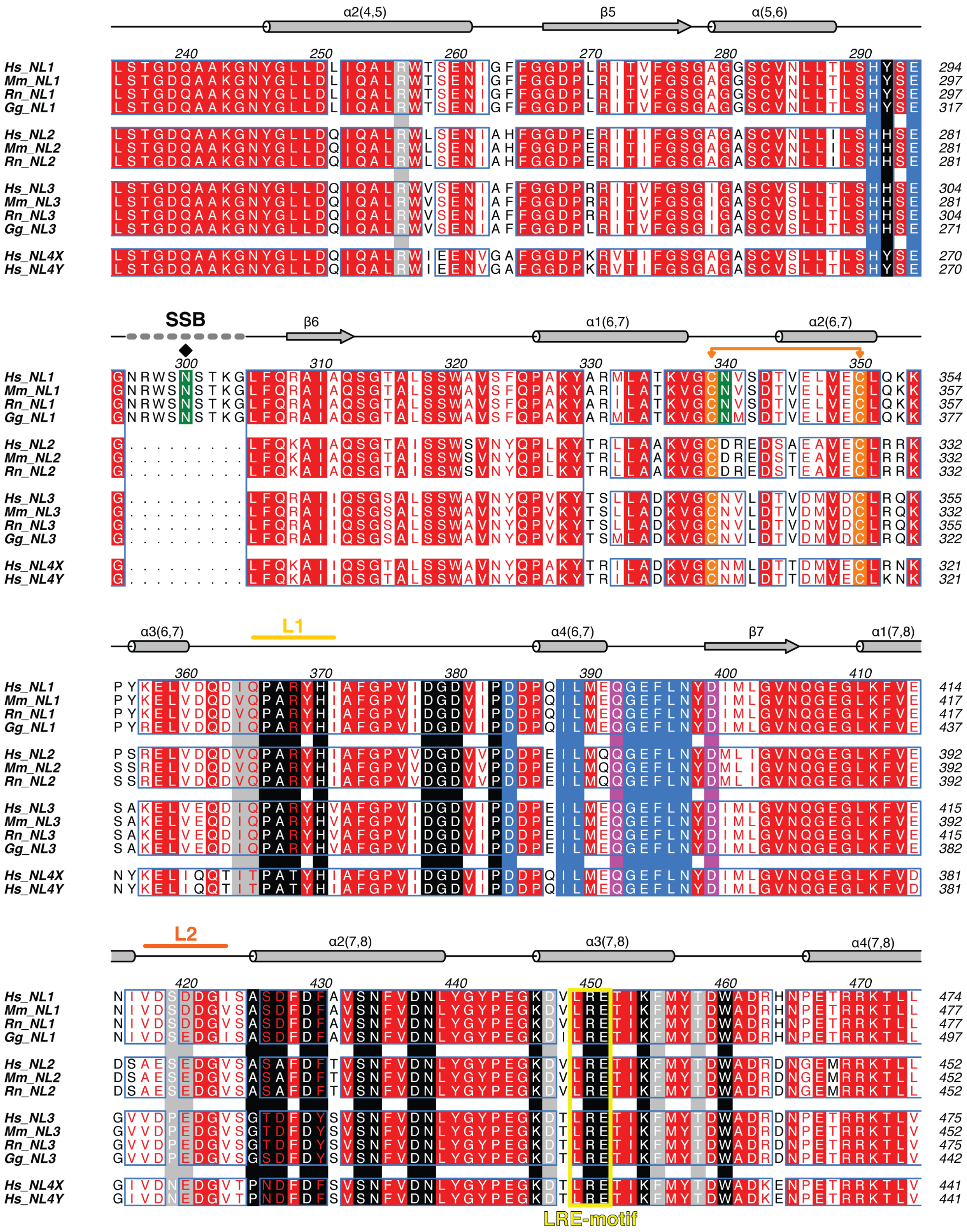

α2(4,5)
β5
α(5,6)
Hs_NL1
Mm_NL1
Rn_NL1
Gg_NL1
Hs_NL2
Mm_NL2
Rn_NL2
Hs_NL3
Mm_NL3
Rn_NL3
Gg_NL3
Hs_NL4X
Hs_NL4Y
SSB
β6
α1(6,7)
α2(6,7)
α3(6,7)
L1
α4(6,7)
β7
α1(7,8)
L2
α2(7,8)
α3(7,8)
α4(7,8)
LRE-motif

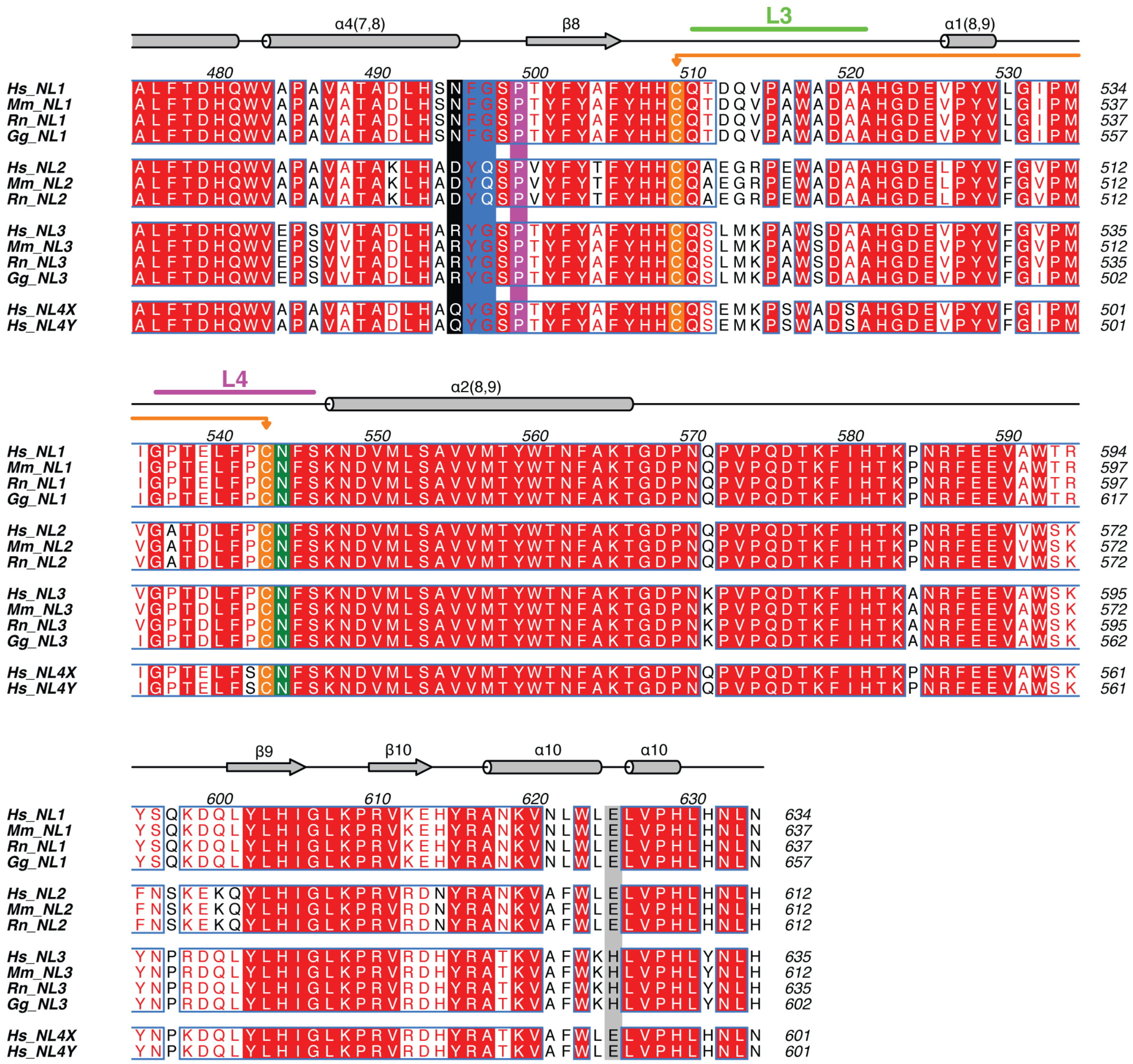

Figure S3. Sequence Alignment of the NL1, -2, -3, -4 and -5 Cholinesterase Domains. Related to Figure 2.

(A) Schematic representation of the human NL1 domain structure. SSA: spliced sequence A, SSB: spliced sequence B, TM: trans-membrane.

(B) Sequences of the different possible NL1, NL2 and NL3 spliced sequences A.

(C) Amino acid sequences are taken from the UniProt database (entry code); *Hs*_NL1 (Q8N2Q7), *Mm*_NL1 (Q99K10), *Rn*_NL1 (Q62765), *Gg*_NL1 (D2X2H3), *Hs*_NL2 (Q8NFZ4), *Mm*_NL2 (Q69ZK9), *Rn*_NL2 (Q62888), *Hs*_NL3 (Q9NZ94), *Mm*_NL3 (Q8BYM5), *Rn*_NL3 (Q62889), *Gg*_NL3 (D3WGL3), *Hs*_NL4X (Q8N0W4), *Hs*_NL4Y (*Hs*_NL5: Q8NFZ3). *Hs*; *Homo sapiens*, *Mm*; *Mus Musculus*, *Rn*; *Rattus norvegicus*, *Gg*; *Gallus gallus*. Secondary structure elements for

*Hs*_NL1 are annotated above the alignment. *Hs*_NL1 cysteine residues participating in disulfide bonds are shaded in orange and connected by orange lines. Putative N-linked glycosylation sites for all NL members are shaded in dark green (black diamonds further highlight crystallographically confirmed N-linked NL1 glycosylation sites). NL residues unique to the "core" and "rim" of the NL–MDGA interface are highlighted in black and grey vertical rectangles, respectively. NL residues unique to the NL–NRX interface are highlighted in pink vertical rectangles. NL residues that are shared between the NL–MDGA and NL–NRX interfaces are highlighted in blue vertical rectangles. The Leu-Arg-Glu (LRE) motif, conserved in all NLs and located in the α3(7,8) helix, is boxed in yellow. The positions of the Cys-loop and of loops L1, L2, L3 and L4 are indicated.

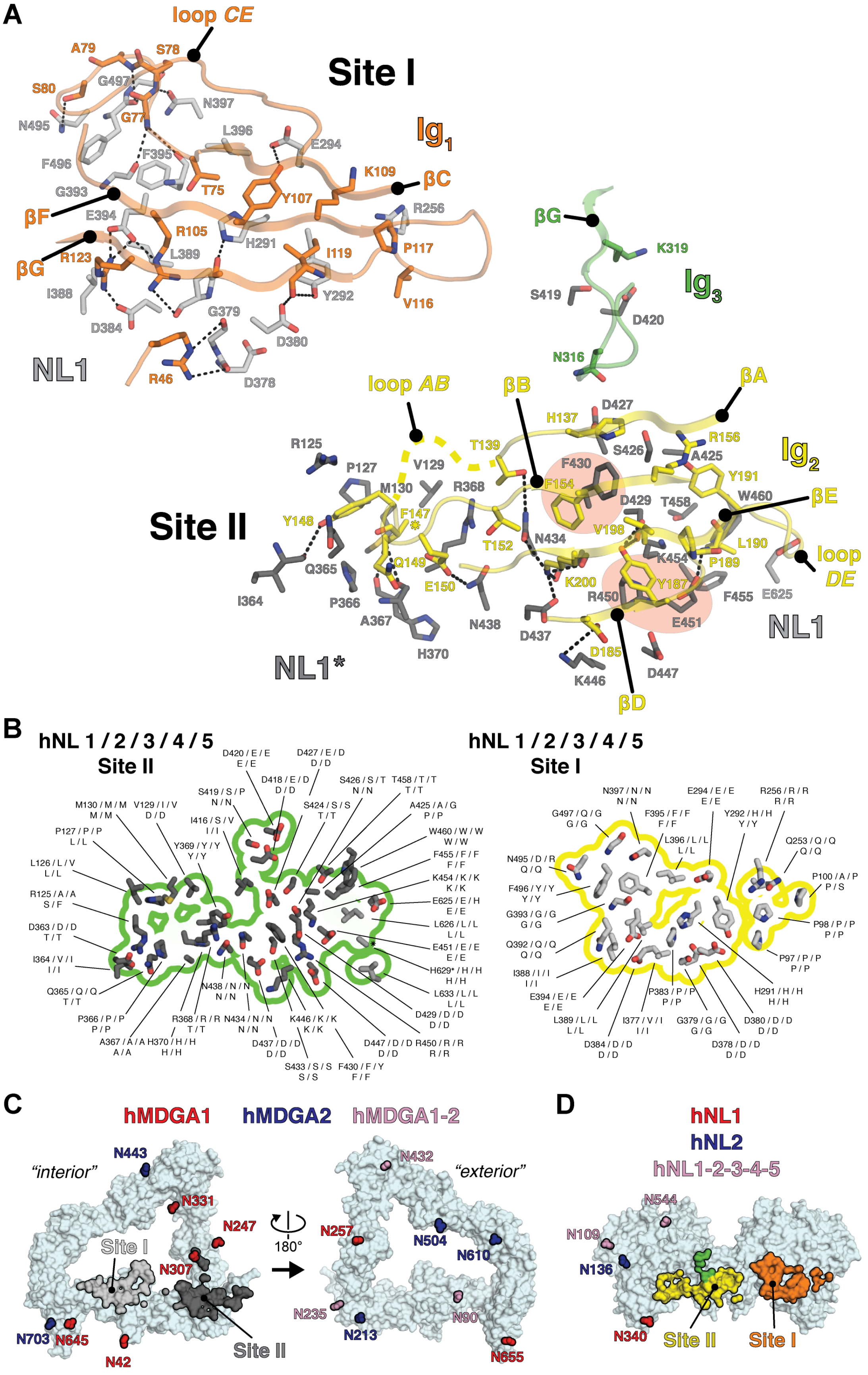

A
loop CE
Site I
A79
S78
S80
G497
N397
N495
G77
L396
Ig1
F395
E294
F496
K109
βC
G393
T75
Y107
βF
E394
R256
R105
H291
βG
R123
L389
I119
P117
βG
K319
I388
Y292
V116
S419
Ig3
D384
G379
D420
D380
NL1
N316
R46
D378
loop AB
βB
βA
H137
D427
R125
T139
R156
S426
A425
Ig2
P127
V129
F430
Y191
F154
R368
M130
W460
D429
T458
βE
Site II
Y148
F147
*
T152
N434
V198
L190
loop DE
K454
P189
Q149
Q365
E150
K200
Y187
I364
R450
F455
E625
P366
N438
A367
E451
NL1
D437
H370
D185
NL1*
D447
K446
βD
B
hNL 1 / 2 / 3 / 4 / 5
Site II
hNL 1 / 2 / 3 / 4 / 5
Site I
D420 / E / E
E / E
D427 / E / D
D / D
D418 / E / D
D / D
S426 / S / T
N / N
T458 / T / T
T / T
S419 / S / P
N / N
S424 / S / S
T / T
A425 / A / G
P / P
M130 / M / M
M / M
V129 / I / V
D / D
I416 / S / V
I / I
W460 / W / W
W / W
P127 / P / P
L / L
Y369 / Y / Y
Y / Y
F455 / F / F
F / F
L126 / L / V
L / L
K454 / K / K
K / K
R125 / A / A
S / F
E625 / E / H
E / E
D363 / D / D
T / T
L626 / L / L
L / L
I364 / V / I
I / I
E451 / E / E
E / E
Q365 / Q / Q
T / T
H629* / H / H
H / H
N438 / N / N
N / N
L633 / L / L
L / L
D429 / D / D
D / D
P366 / P / P
P / P
R368 / R / R
T / T
N434 / N / N
N / N
K446 / K / K
K / K
A367 / A / A
A / A
H370 / H / H
H / H
D437 / D / D
D / D
D447 / D / D
D / D
R450 / R / R
R / R
S433 / S / S
S / S
F430 / F / Y
F / F
N397 / N / N
N / N
E294 / E / E
E / E
R256 / R / R
R / R
G497 / Q / G
G / G
F395 / F / F
F / F
Y292 / H / H
Y / Y
L396 / L / L
L / L
Q253 / Q / Q
Q / Q
N495 / D / R
Q / Q
P100 / A / P
P / S
F496 / Y / Y
Y / Y
G393 / G / G
G / G
P98 / P / P
P / P
Q392 / Q / Q
Q / Q
P97 / P / P
P / P
I388 / I / I
I / I
H291 / H / H
H / H
E394 / E / E
E / E
P383 / P / P
P / P
L389 / L / L
L / L
I377 / V / I
I / I
G379 / G / G
G / G
D380 / D / D
D / D
D384 / D / D
D / D
D378 / D / D
D / D
C
hMDGA1
hMDGA2
hMDGA1-2
"interior"
"exterior"
N443
N432
N331
N247
180°
N257
N504
Site I
N610
N307
N235
N90
N703
N645
N213
Site II
N655
N42
D
hNL1
hNL2
hNL1-2-3-4-5
N544
N109
N136
N340
Site II
Site I

Figure S4. Details and Conservation of the NL–MDGA Site I and Site II Interfaces. Mapping of Human MDGA1 and MDGA2 Glycosylation Sites. Related to Figure 3.

(A) Atomic details of the NL1–MDGA1 Site I and Site II interfaces. Putative hydrogen bonds and hydrophilic interactions are indicated with black dashed lines. Star symbols (*) indicate residues for which side chain electron density was not clearly discernable. The $Phe_{NL1}430$-$Phe_{MDGA1}154$ π-π sandwich stacking interaction, as well as $Arg_{NL1}450$ and $Glu_{NL1}451$ of the NL1 LRE motif, are highlighted with shaded ovals.

(B) View of the NL1 interaction interface. Site I and Site II interfaces are outlined by yellow and green lines, respectively. Per residue position, equivalent residues in human NL1, -2, -3, -4(X) and -5(=4(Y)) are annotated to highlight overall sequence conservation of the interaction interfaces. Star symbols (*) indicate residues for which side chain electron density was not clearly discernable.

(C) N-linked glycosylation sites common to human MDGA1 and -2 (pink), or unique to human MDGA1 (red) or human MDGA2 (blue) are annotated onto the $cMDGA1_{ECTO}$ structure. Site I and Site II interfaces are shown in surface representation. The hMDGA1-specific Asn307 (N307) is proximal to the edge of the Site II interface.

(D) N-linked glycosylation sites common to human NL1-2-3-4-5 (pink), or unique to human NL1 (red) or human NL2 (blue) are annotated onto the $hNL1(\text{–A–B})_{ECTO}$ structure. Site I and Site II interfaces are shown in surface representation as in Figure 3A.

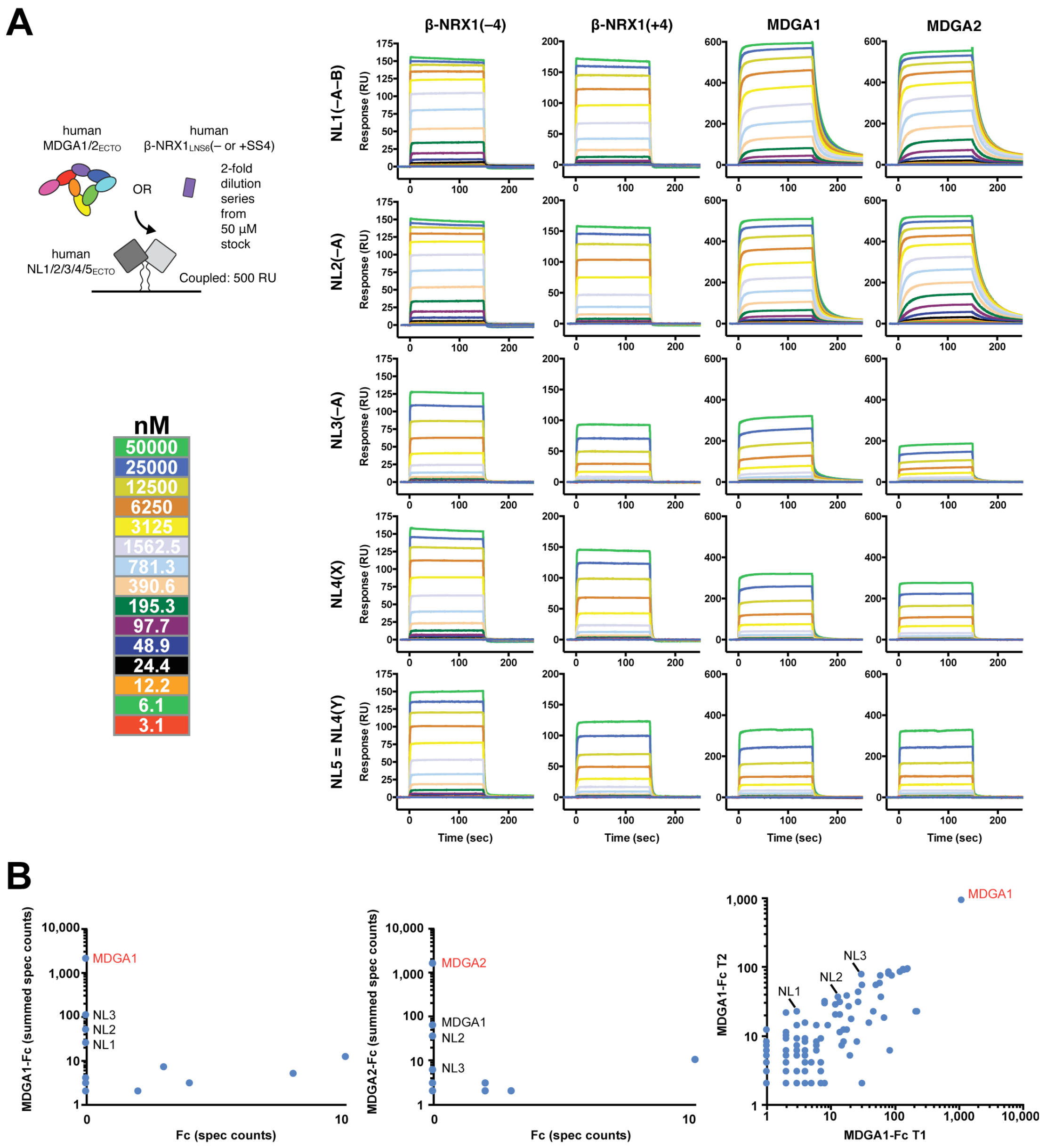

Figure S5. NL–NRX and NL–MDGA SPR Equilibrium Experiments. MDGA1 and -2 Pulldown Experiments. Related to Figure 5.

(A) Schematic representation of the SPR setup, and sensorgrams for the interaction of the human NL1, NL2, NL3, NL4 and NL5 cholinesterase domains, lacking splice inserts, with human β-NRX1$_{LNS6}$ lacking and containing spliced sequence 4 (β-NRX1$_{LNS6}$(–4) and β-NRX1$_{LNS6}$(+4), respectively), and with human MDGA1$_{ECTO}$ and MDGA2$_{ECTO}$. The corresponding binding isotherms and K_D values are presented in Figure 5A.

(B) The graphs show the summed spectra count (spec count) for all surface proteins identified in two independent MDGA1-Fc or MDGA2-Fc pulldown experiments from rat brain synaptosome extracts, each compared to two negative control Fc experiments. NL1-3 are the main surface proteins specifically identified by MDGA1-Fc. MDGA2-Fc bait protein identifies NL2 and NL3 as interactors.

A

	DNA amount (μg) / plasmid ratio vs. NL1/2/3/4$_{FL}$		
	LOW	MEDIUM	HIGH
MDGA1$_{FL}$	1.1 / 2.2	1.75 / 3.5	2.5 / 5.0
MDGA2$_{FL}$	1.6 / 3.2	2.5 / 5.0	3.6 / 7.2
CD4	1.1 / 2.2	1.75 / 3.5	2.5 / 5.0
NL1/2/3/4$_{FL}$	0.5	0.5	0.5

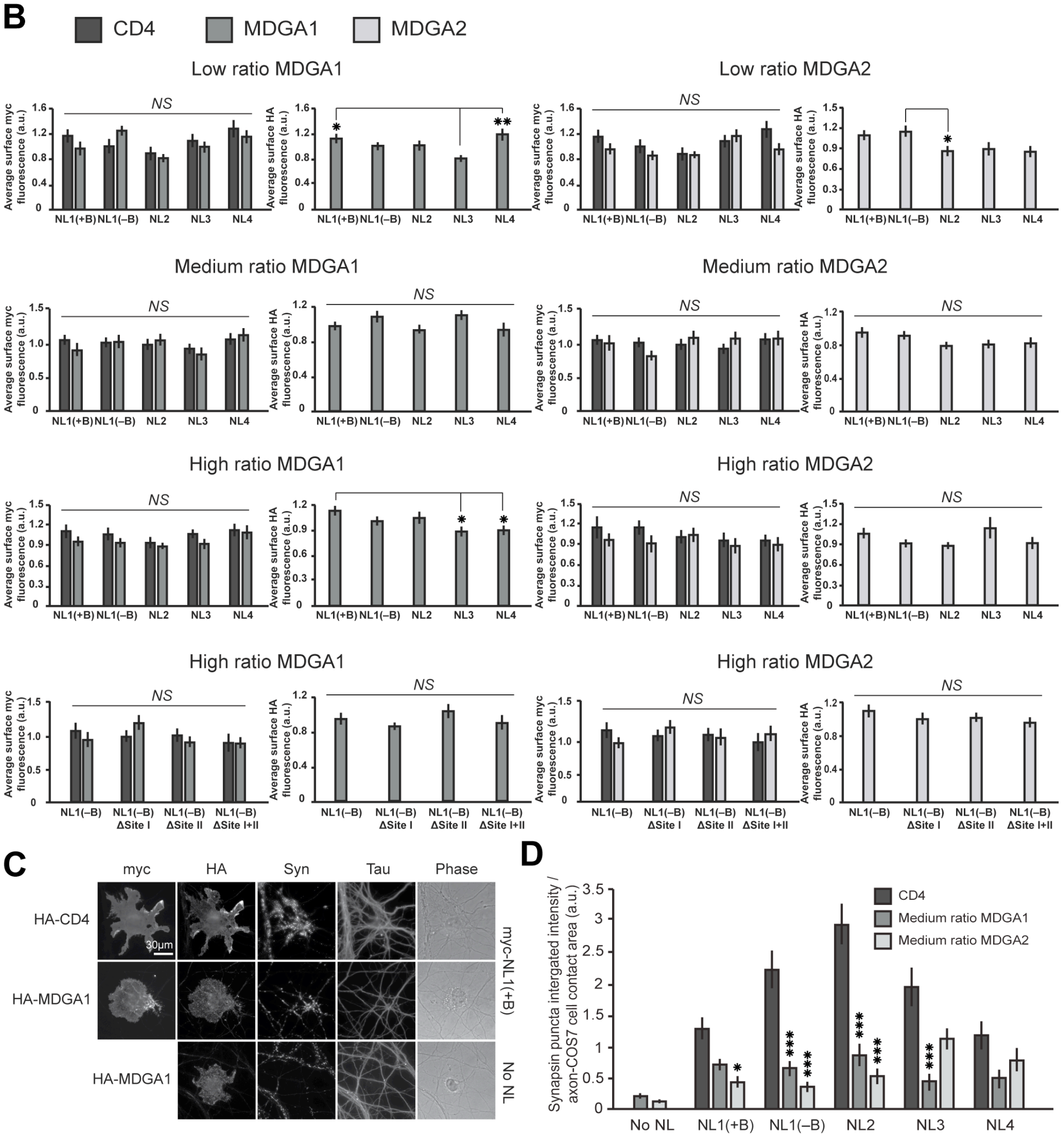

Figure S6. Comparable Levels of Surface NLs And MDGAs in the Hemi-synapse Formation Assays. Related to Figure 5, Figure 6, and Figure 8.

(A) Summary of MDGA:NL plasmid DNA ratios.

(B) COS-7 cells chosen for analysis displayed similar levels of myc-NL1-4 and HA-MDGA1-2 in each co-culture experiment. For each condition (low ratio MDGA1, medium ratio MDGA1,

high ratio MDGA1, low ratio MDGA2, medium ratio MDGA2, and high ratio MDGA2) the mean myc signal (for surface NLs) and mean HA signal (for surface MDGAs) was measured and cells that did not have similar levels of each protein were excluded from analysis. One-way ANOVA with Bonferonni *post hoc* comparison was used to determine statistical significance. Error bars represent the SEM. *; $p < 0.05$, **; $p < 0.01$.

(C) Examples are shown for all the channels imaged for myc-NL1(–A+B) co-transfected with HA-CD4 or HA-MDGA1, and for the HA-MDGA1 only (no NL) negative control. Scale bar is 30 μm.

(D) The bar graph shows the hemi-synapse formation assay data for MDGA1 and MDGA2 at medium ratio without normalization. Each NL co-transfected with CD4 shows a different baseline level of synapsin clustering, with NL2 being the most potent and NL4 being the least potent at hemi-synapse induction. Error bars represent the SEM. *; $p < 0.05$, ***; $p < 0.001$.

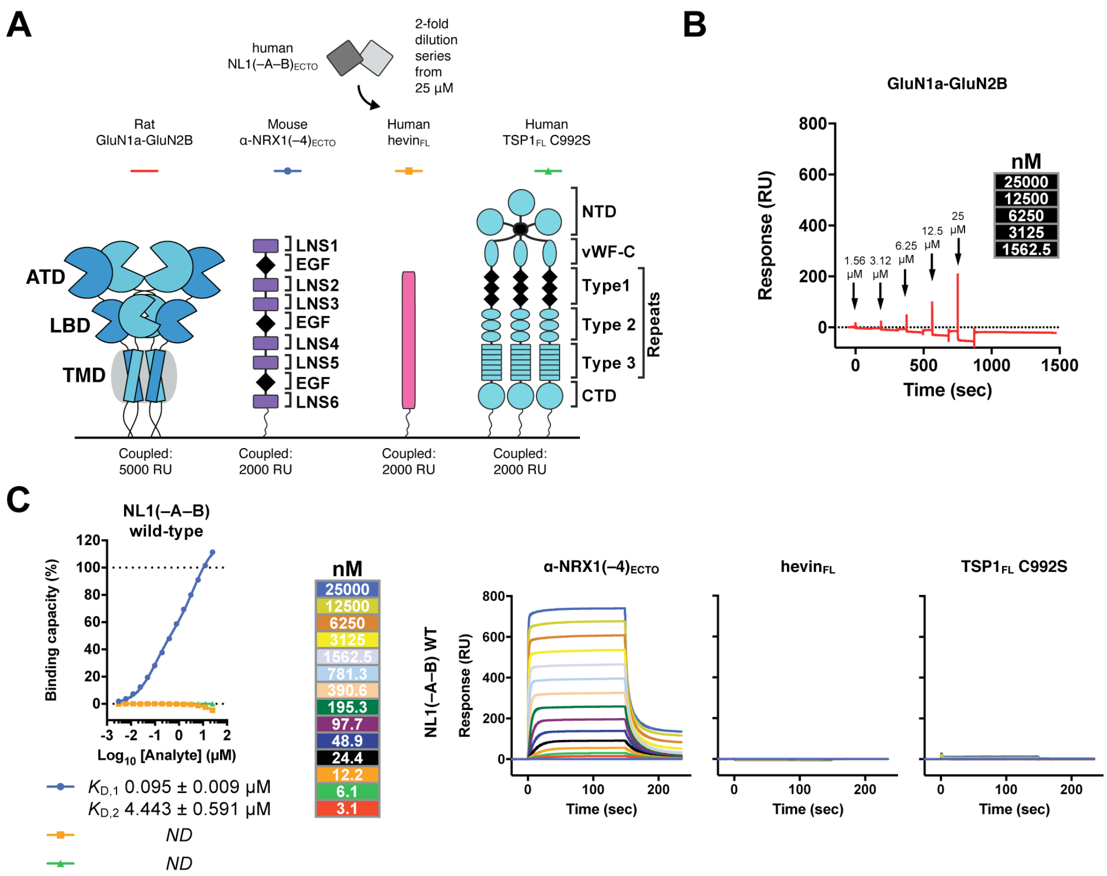

Figure S7. Assessment of Binding of NL1 with Hevin, Thrombospondin-1 and the NMDA Receptor. Related to Figure 5.

(A) Schematic representation of the SPR setup.

(B) Single-cycle kinetics (SCK) sensorgram of the interaction of NL1(–A–B)$_{ECTO}$ with the GluN2a–GluN2B NMDA receptor.

(C) Binding isotherms and sensorgrams for the interaction of the human NL1(–A–B)$_{ECTO}$ cholinesterase domains with mouse α-NRX1$_{ECTO}$(–4), full-length (FL) human hevin (hevin$_{FL}$) and human TSP1$_{FL}$.

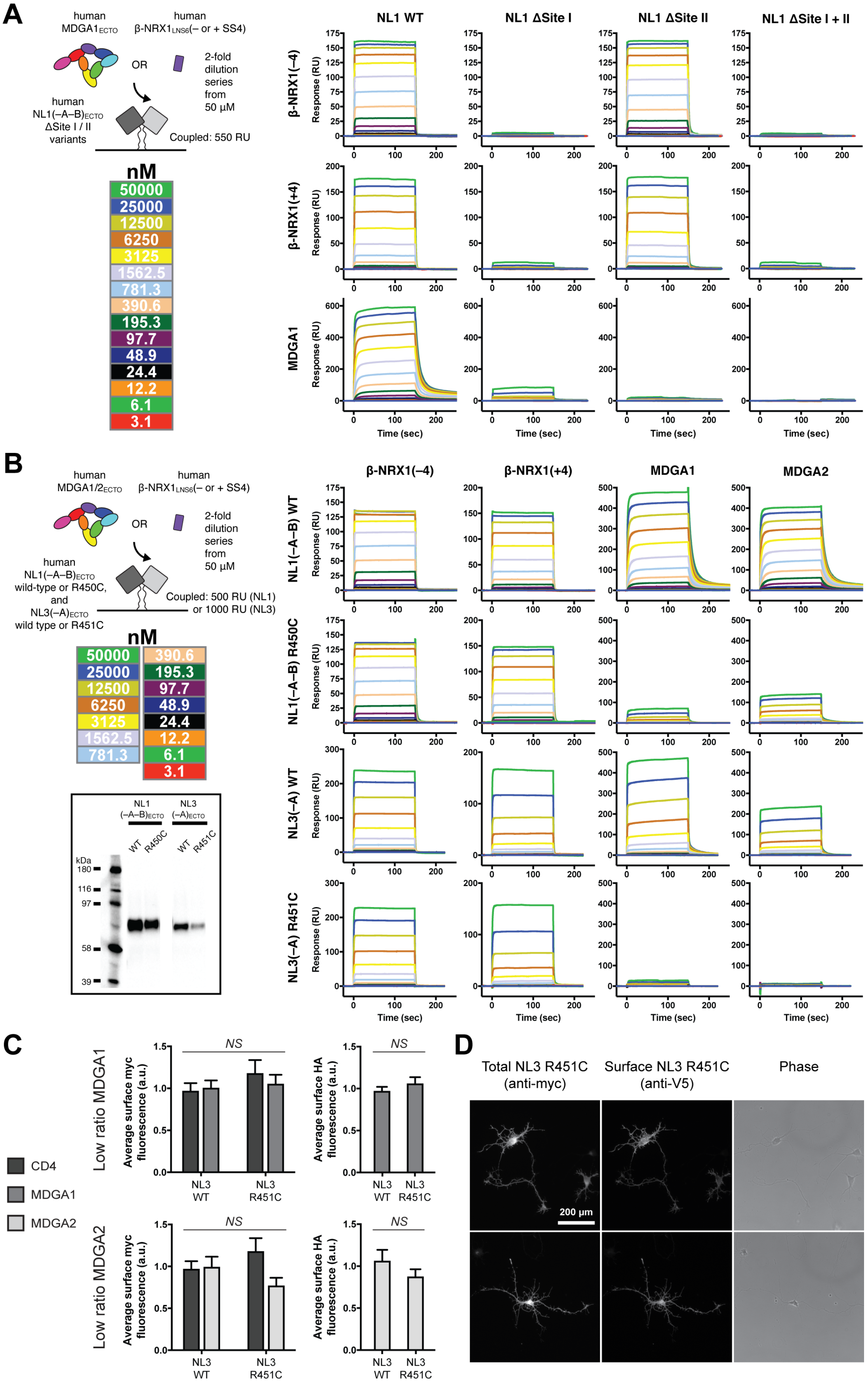
A
human MDGA1ECTO
human β-NRX1LNS6(– or + SS4)
OR
2-fold dilution series from 50 µM
human NL1(–A–B)ECTO ΔSite I / II variants
Coupled: 550 RU
nM
50000
25000
12500
6250
3125
1562.5
781.3
390.6
195.3
97.7
48.9
24.4
12.2
6.1
3.1
NL1 WT
NL1 ΔSite I
NL1 ΔSite II
NL1 ΔSite I + II
β-NRX1(–4)
β-NRX1(+4)
MDGA1
Response (RU)
Time (sec)
B
human MDGA1/2ECTO
human β-NRX1LNS6(– or + SS4)
OR
2-fold dilution series from 50 µM
human NL1(–A–B)ECTO wild-type or R450C, and NL3(–A)ECTO wild type or R451C
Coupled: 500 RU (NL1) or 1000 RU (NL3)
nM
β-NRX1(–4)
β-NRX1(+4)
MDGA1
MDGA2
NL1(–A–B) WT
NL1(–A–B) R450C
NL3(–A) WT
NL3(–A) R451C
Response (RU)
Time (sec)
NL1 (–A–B)ECTO
NL3 (–A)ECTO
WT
R450C
WT
R451C
kDa
180
116
97
58
39
C
CD4
MDGA1
MDGA2
Low ratio MDGA1
Low ratio MDGA2
Average surface myc fluorescence (a.u.)
Average surface HA fluorescence (a.u.)
NS
NL3 WT
NL3 R451C
D
Total NL3 R451C (anti-myc)
Surface NL3 R451C (anti-V5)
Phase
200 µm

Figure S8. Mutational Analysis of the NL1 Site I and Site II Interfaces. Effect of the ASD-linked NL3 Mutation Arg451Cys on the Interaction of NL3 with MDGA1 and MDGA2. Related to Figure 6 and Figure 7.

(A) Schematic representation of the SPR setup, and sensorgrams for the interaction of wild-type human NL1(–A–B)$_{ECTO}$ and the human NL1(–A–B)$_{ECTO}$ Site I (ΔSite I), Site II (ΔSite II) and Site I+II (ΔSite I+II) core interface mutants, with human β-NRX1$_{LNS6}$ lacking and containing spliced sequence 4 (β-NRX1$_{LNS6}$(–4) and β-NRX1$_{LNS6}$(+4), respectively), and with human MDGA1$_{ECTO}$. Consistent with the respective crystal structures (Figure 4A), ΔSite II abolishes MDGA1 binding, but does not affect β-NRX1 binding. The corresponding binding isotherms and K_D values are presented in Figure 6B.

(B) Schematic representation of the SPR setup, and sensorgrams for the interaction of the human NL1, NL1 Arg450Cys, NL3 and NL3 Arg451Cys cholinesterase domains, lacking splice inserts, with human β-NRX1$_{LNS6}$ lacking and containing spliced sequence 4 (β-NRX1$_{LNS6}$(–4) and β-NRX1$_{LNS6}$(+4), respectively), and with human MDGA1$_{ECTO}$ and MDGA2$_{ECTO}$. The corresponding binding isotherms and K_D values are presented in Figure 7C. The inset shows a Western blot of the NL1(–A–B)$_{ECTO}$-WT, NL1(–A–B)$_{ECTO}$-R450C, NL3(–A)$_{ECTO}$-WT and NL3(–A)$_{ECTO}$-R451C proteins secreted by HEK293T cells into the growth medium. Detection of the biotinylated Avitag was performed using streptavidin-HRP conjugate (1:50,000 dilution, Sigma Aldrich).

(C) Comparable levels of surface NL3 and MDGAs in the hemi-synapse formation assay. COS-7 cells chosen for analysis displayed similar levels of myc-NL3 and HA-MDGA1-2 in each co-culture experiment. The mean myc signal (for surface NL3s) and mean HA signal (for surface MDGAs) was measured and cells that did not have similar levels of each protein were excluded from analysis. One-way ANOVA with Bonferonni *post hoc* comparison was used to determine statistical significance. Error bars represent the SEM.

(D) Expression of full-length human Myc-V5-NL3-R451C on the cell surface of rat hippocampal neurons. Representative images of neurons immunostained for total myc-NL3 or for surface V5-NL3 are shown. Scale bar is 200 μm.

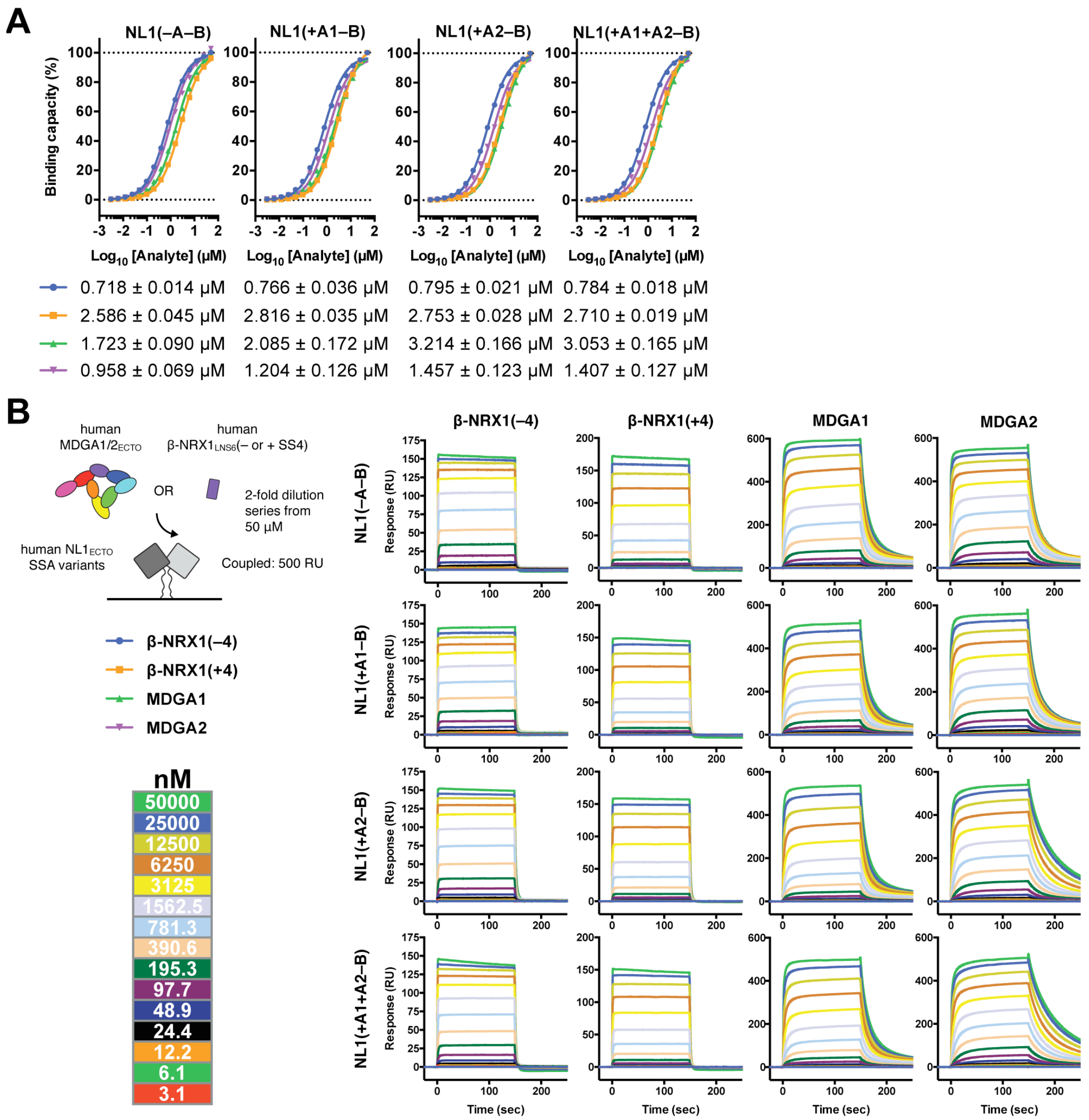

Figure S9. NL1 Spliced Sequence A (SSA) Does Not Modulate NL1–NRX or NL1–MDGA Interactions. Related to Figure 8.

(A) Binding isotherms and summary of K_D values for the interaction of the human NL1(±A1±A2–B)ECTO cholinesterase domains with human β-NRX1LNS6 lacking and containing spliced sequence 4 (β-NRX1LNS6(–4) and β-NRX1LNS6(+4), respectively), and with human MDGA1ECTO and MDGA2ECTO. Data for NL1(–A–B)ECTO have been repeated here from Figures 5A and S5A for clearer comparison.

(B) Schematic representation of the SPR setup and sensorgrams corresponding to (A).

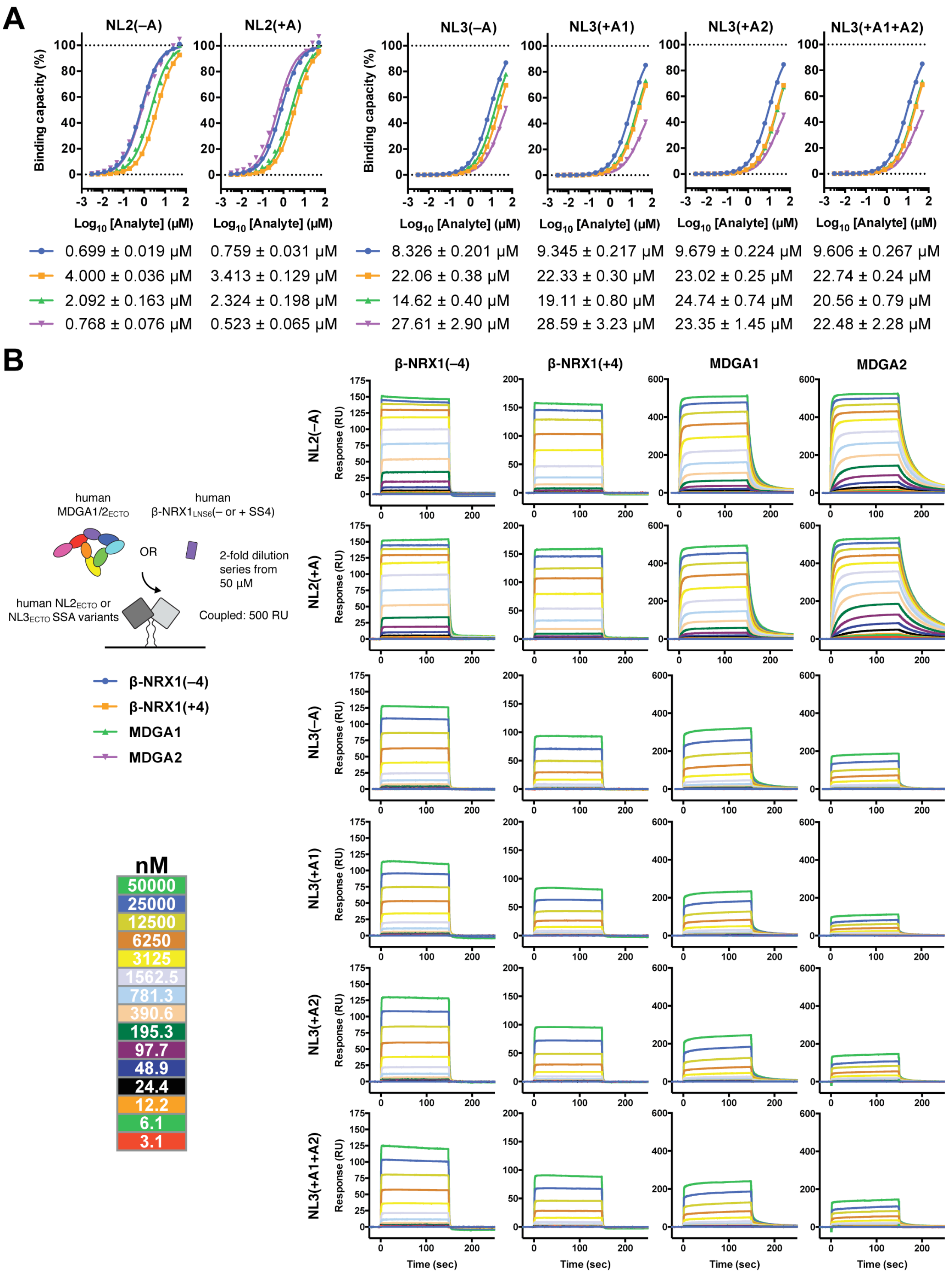

Figure S10. NL2 or NL3 Spliced Sequences A (SSA) Do Not Modulate NL2/3–NRX or NL2/3–MDGA Interactions. Related to Figure 8.

(A) Binding isotherms and summary of K_D values for the interaction of the human NL2(±A)$_{ECTO}$ or NL3(±A1±A2)$_{ECTO}$ cholinesterase domains with human β-NRX1$_{LNS6}$ lacking and containing

spliced sequence 4 (β-NRX1$_{LNS6}$(–4) and β-NRX1$_{LNS6}$(+4), respectively), and with human MDGA1$_{ECTO}$ and MDGA2$_{ECTO}$. Data for NL2(–A)$_{ECTO}$ and NL3(–A)$_{ECTO}$ have been repeated here from Figures 5A and S5A for clearer comparison.

(B) Schematic representation of the SPR setup and sensorgrams corresponding to (A)**.**

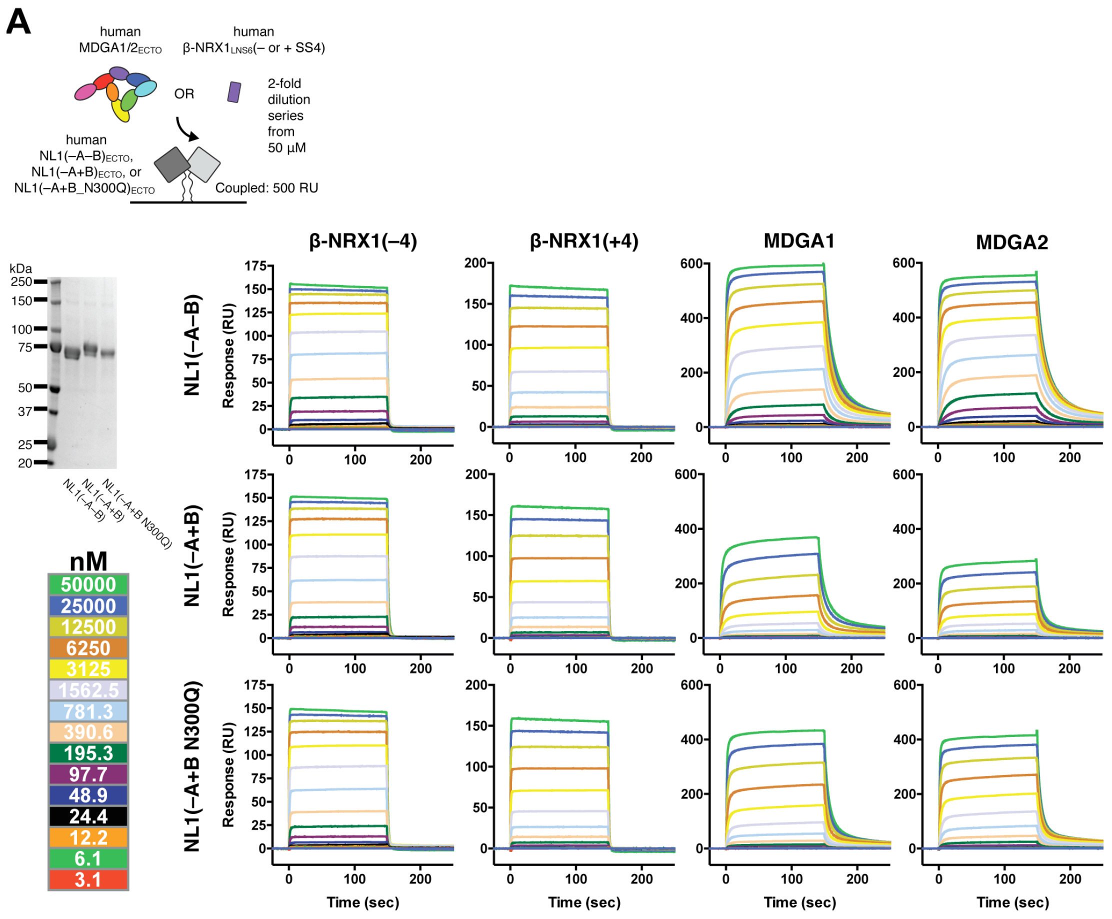

Figure S11. NL1 Spliced Sequence B (SSB) Differentially Modulates NL1–NRX and NL1–MDGA Interactions. Related to Figure 8.

(A) Schematic representation of the SPR setup, and sensorgrams for the interaction of human NL1(–A–B)$_{ECTO}$, NL1(–A+B)$_{ECTO}$ and NL1(–A+B_Asn300Gln)$_{ECTO}$ with human β-NRX1$_{LNS6}$ lacking and containing spliced sequence 4 (β-NRX1$_{LNS6}$(–4) and β-NRX1$_{LNS6}$(+4), respectively), and with human MDGA1$_{ECTO}$ and MDGA2$_{ECTO}$. The SDS-PAGE gel shows NL1(–A–B)$_{ECTO}$, NL1(–A+B)$_{ECTO}$ and NL1(–A+B_Asn300Gln)$_{ECTO}$, expressed in HEK 293T cells, indicating their relative differences in apparent molecular weight due to their different glycosylation states. Data for NL1(–A–B)$_{ECTO}$ have been repeated here from Figures 5A and S5A for clearer comparison. The corresponding binding isotherms and K_D values are presented in Figure 8C.

Table S1. Crystallographic Data Collection and Refinement Statistics. Related to Figure 1, Figure 2, and Figure 8.

	**hNL1 (–A+B) $_{ECTO}$**	**cMDGA1$_{ECTO}$ SeMet-labeled**	**hNL1(–A–B)$_{ECTO}$–cMDGA1$_{ECTO}$**
PDB code	5OJK	5OJ2	5OJ6
**DATA COLLECTION**			
Source	DLS I24	DLS I03	DLS I04-1
Wavelength λ (Å)	0.96860	0.97938 (peak)	0.92000
No. of crystals	1	1	1
Resolution (Å)	51.70-2.55 (2.62-2.55)	96.65-3.20 (3.28-3.20)	92.07-3.30 (3.39-3.30)
Space group	$P22_12_1$	$P2_12_12_1$	$P2_12_12$
Cell dimensions; a, b, c (Å)	49.57, 118.05, 214.34	103.18, 109.04, 208.77	109.42, 184.14, 96.47
Unique reflections	40826 (3077)	39616 (2874)	29576 (2080)
Multiplicity	7.5 (7.3)	11.0 (10.9)	4.5 (4.6)
Completeness (%)	97.3 (98.8)	100.0 (99.8)	98.7 (95.4)
R_{MERGE} (%)	17.9 (126.6)	14.8 (134.8)	7.5 (84.0)
R_{MEAS} (%)	19.3 (136.2)	15.5 (141.5)	9.5 (106.3)
R_{PIM} (%)	6.8 (48.4)	4.6 (42.3)	5.8 (64.1)
$CC_{1/2}$ (%)	99.6 (54.1)	99.8 (62.2)	99.9 (63.8)
CC* (%)	99.9 (83.8)	99.9 (87.6)	99.9 (88.3)
Average $I/\sigma(I)$	8.4 (1.5)	13.6 (1.8)	15.6 (1.6)
**REFINEMENT**			
Resolution (Å)	51.70-2.55 (2.61-2.55)	96.65-3.20 (3.24-3.20)	72.38-3.30 (3.40-3.30)
Reflections (WORK / FREE set)	38734 / 2005	35762 / 3851	28098 / 1474
R_{WORK} / R_{FREE} (%)	0.2360 / 0.2780 (0.3331 / 0.3658)	0.2144 / 0.2514 (0.3767 / 0.3808)	0.2269 / 0.2771 (0.3181 / 0.3838)
CC_{WORK} / CC_{FREE} in highest resolution shell (%)	0.732 / 0.629	0.579 / 0.563	0.814 / 0.676
No. of atoms (NL1 / MDGA1 / NAG-BMA-MAN / PGE / water)	8406 / 0 / 14 / 20 / 109	0 / 10788 / 299 / 0 / 0	4278 / 5381 / 151 / 0 / 0
B factors (Å^2) (NL1 / MDGA1 / NAG-BMA-MAN / PGE / water)	50.8 / - / 71.3 / 52.9 / 44.6	- / 105.2 / 146.7 / - / -	92.5 / 129.6 / 158.0 / - / -
R.m.s.d. Bonds (Å)	0.003	0.004	0.003
R.m.s.d. angles (°)	0.782	0.986	0.939
Ramachandran			
Favored (%)	94.79	92.37	92.78
Allowed (%)	5.21	7.56	7.14
Outliers (%)	0.00	0.07	0.08
Molprobity score / percentile	1.58 / 99th	1.82 / 100th	1.99 / 100th

Numbers in parentheses refer to the highest resolution shell.

R.m.s.d.: root mean square deviation from ideal geometry.

R_{PIM}: precision-indicating merging R-factor.

R_{MEAS}: multiplicity-corrected R_{SYM}.

$CC_{1/2}$, CC^*: correlation coefficients between random half data sets.

CC_{WORK} / CC_{FREE}: standard and cross-validated correlations of the experimental intensities with the intensities calculated from the refined molecular model.

NAG: N-Acetyl-D-Glucosamine.

BMA: Beta-D-mannose.

MAN: Alpha-D-mannose.

PGE: Triethylene glycol.

Table S2. Mass Spectrometry Summary Data File. Related to Figure S5.

Mass spectrometry summary data file for MDGA1 (two independent experiments), MDGA2 (two independent experiments), and MDGA1ΔIg1-3 (one experiment). Column headers containing spectra counts are highlighted in bold. Protein descriptions are listed in the far-right column. Data are sorted by MDGA1-Fc spectra count in descending order.

Table S3. One-way Analysis of Variance of the Effect of Co-expression of NL with MDGA1 or MDGA2 at Low, Medium and High Plasmid Ratios. Related to Figure 5 and Figure 8.

ANOVA	F value			*p* value
MDGA1 : NL1-4 low	9.87			< 0.0001
Bonferroni post hoc comparison	Mean difference	t value	Significant at $p < 0.05$	95% confidence interval of mean difference
NL1(+B): CD4 vs. MDGA1	0.1009	0.4912	No	-0.5770 to 0.7789
NL1(–B): CD4 vs. MDGA1	0.1761	0.8487	No	-0.5085 to 0.8607
NL2: CD4 vs. MDGA1	1.526	6.791	Yes	0.7849 to 2.268
NL3: CD4 vs. MDGA1	0.9278	4.497	Yes	0.2471 to 1.608
NL4: CD4 vs. MDGA1	0.02018	0.0918	No	-0.7054 to 0.7458

ANOVA	F value			*p* value
MDGA1 : NL1-4 medium	15.18			< 0.0001
Bonferroni post hoc comparison	Mean difference	t value	Significant at $p < 0.05$	95% confidence interval of mean difference
NL1(+B): CD4 vs. MDGA1	0.5515	1.892	No	-0.4076 to 1.511
NL1(–B): CD4 vs. MDGA1	1.541	5.076	Yes	0.5423 to 2.540
NL2: CD4 vs. MDGA1	2.028	6.794	Yes	1.046 to 3.010
NL3: CD4 vs. MDGA1	1.484	4.861	Yes	0.4795 to 2.488
NL4: CD4 vs. MDGA1	0.6552	2.026	No	-0.4087 to 1.719

ANOVA	F value			*p* value
MDGA1 : NL1-4 high	31.63			< 0.0001
Bonferroni post hoc comparison	Mean difference	t value	Significant at $p < 0.05$	95% confidence interval of mean difference
NL1(+B): CD4 vs. MDGA1	0.9434	4.373	Yes	0.2338 to 1.653
NL1(–B): CD4 vs. MDGA1	2.165	10.35	Yes	1.477 to 2.853
NL2: CD4 vs. MDGA1	1.856	8.241	Yes	1.115 to 2.596
NL3: CD4 vs. MDGA1	1.457	6.674	Yes	0.7390 to 2.175
NL4: CD4 vs. MDGA1	0.4879	2.106	No	-0.2741 to 1.250

ANOVA	F value			*p* value
MDGA2 : NL1-4 low	14.83			< 0.0001
Bonferroni post hoc comparison	Mean difference	t value	Significant at $p < 0.05$	95% confidence interval of mean difference
NL1(+B): CD4 vs. MDGA2	0.7083	3.642	Yes	0.06660 to 1.350

NL1(–B): CD4 vs. MDGA2	1.273	6.726	Yes	0.6488 to 1.898
NL2: CD4 vs. MDGA2	1.653	7.840	Yes	0.9573 to 2.349
NL3: CD4 vs. MDGA2	0.4456	2.302	No	-0.1929 to 1.084
NL4: CD4 vs. MDGA2	-0.06702	0.3139	No	-0.7716 to 0.6375

ANOVA	F value			*p* value
MDGA2 : NL1-4 medium	14.08			< 0.0001
Bonferroni post hoc comparison	Mean difference	t value	Significant at $p < 0.05$	95% confidence interval of mean difference
NL1(+B): CD4 vs. MDGA2	1.031	3.334	Yes	0.01342 to 2.048
NL1(–B): CD4 vs. MDGA2	1.825	5.805	Yes	0.7903 to 2.859
NL2: CD4 vs. MDGA2	2.376	7.688	Yes	1.359 to 3.393
NL3: CD4 vs. MDGA2	0.7810	2.354	No	-0.3110 to 1.873
NL4: CD4 vs. MDGA2	0.3797	1.125	No	-0.7313 to 1.491

ANOVA	F value			*p* value
MDGA2 : NL1-4 high	13.61			< 0.0001
Bonferroni post hoc comparison	Mean difference	t value	Significant at $p < 0.05$	95% confidence interval of mean difference
NL1(+B): CD4 vs. MDGA2	0.8524	3.362	Yes	0.01736 to 1.687
NL1(–B): CD4 vs. MDGA2	0.9596	3.702	Yes	0.1059 to 1.813
NL2: CD4 vs. MDGA2	1.717	6.998	Yes	0.9088 to 2.524
NL3: CD4 vs. MDGA2	0.5741	2.304	No	-0.2463 to 1.395
NL4: CD4 vs. MDGA2	0.6755	2.762	No	-0.1299 to 1.481

Table S4. One-way Analysis of Variance of the Effect of NL(–B) ΔSite I, ΔSite II and ΔSite I+II Mutant Co-expression with MDGA1 and MDGA2 at High Plasmid Ratio. Related to Figure 6.

ANOVA	F value			p value
MDGA : NL1(–B) ΔI ΔII high	24.16			< 0.0001
Bonferroni post hoc comparison	Mean difference	t value	Significant at $p < 0.05$	95% confidence interval of mean difference
NL1(–B) WT: CD4 vs. MDGA1	2.526	8.856	Yes	1.531 to 3.520
NL1(–B) ΔI: CD4 vs. MDGA1	-0.1027	0.3800	No	-1.045 to 0.8395
NL1(–B) ΔII: CD4 vs. MDGA1	-0.5891	2.029	No	-1.601 to 0.4232
NL1(–B) ΔI+II: CD4 vs. MDGA1	-0.06621	0.2213	No	-1.109 to 0.9770
NL1(–B) WT: CD4 vs. MDGA2	2.217	7.486	Yes	1.184 to 3.250
NL1(–B) ΔI: CD4 vs. MDGA2	-0.01825	0.06645	No	-0.9757 to 0.9392
NL1(–B) ΔII: CD4 vs. MDGA2	-0.7561	2.655	No	-1.749 to 0.2369
NL1(–B) ΔI+II: CD4 vs. MDGA2	0.05128	0.1676	No	-1.015 to 1.118

Table S5. One-way Analysis of Variance of the Effect of NL3 R451C Mutant Co-expression with MDGA1 and MDGA2 at Low Plasmid Ratio. Related to Figure 7.

ANOVA	F value			p value
MDGA : NL3 low	14.78			< 0.0001
Bonferroni post hoc comparison	Mean difference	t value	Significant at $p < 0.05$	95% confidence interval of mean difference
NL3 WT: CD4 vs. MDGA1	1.077	4.845	Yes	0.3727 to 1.782
NL3 WT: CD4 vs. MDGA2	-0.03604	0.1495	No	-0.7999 to 0.7279
NL3 R451C: CD4 vs. MDGA1	0.2690	1.052	No	-0.5415 to 1.079
NL3 R451C: CD4 vs. MDGA2	0.1859	0.6730	No	-0.6895 to 1.061
