# Supplementary material for: Structural Mechanism for Modulation of Synaptic Neuroligin-Neurexin Signaling by MDGA Proteins
Source: Neuron. 2017 Aug 16;95(4):896–913.e10. doi: 10.1016/j.neuron.2017.07.040 (PMC5563082; doi:10.1016/j.neuron.2017.07.040)
Supplement: Document S2. Article plus Supplemental Information [file mmc3.pdf]

Neuron

Article

Structural Mechanism for Modulation of Synaptic Neuroligin-Neurexin Signaling by MDGA Proteins

Highlights

- The MDGA1 extracellular region has an unusual triangular multi-domain arrangement
- The NL1-MDGA1 complex structure reveals how MDGA proteins block neurexin binding
- MDGA1 and MDGA2 bind all NL isoforms, a process fine-tuned by alternative splicing
- MDGA1 and MDGA2 suppress NL synaptogenic activity in a concentration-dependent manner

Authors

Jonathan Elegheert,
Vedrana Cvetkovska,
Amber J. Clayton, ..., Jo Begbie,
Ann Marie Craig, A. Radu Aricescu

Correspondence

jelegheert@strubi.ox.ac.uk (J.E.),
acraig@mail.ubc.ca (A.M.C.),
radu@mrc-lmb.cam.ac.uk (A.R.A.)

In Brief

Elegheert et al. present the crystal structure of the autism-linked post-synaptic protein MDGA in complex with the synapse organizer neuroligin, providing a structural and mechanistic basis for potentially brain-wide modulation of synaptic neuroligin-neurexin signaling by MDGA proteins.

Elegheert et al., 2017, Neuron 95, 896–913
August 16, 2017 © 2017 MRC Laboratory of Molecular Biology.
Published by Elsevier Inc.
http://dx.doi.org/10.1016/j.neuron.2017.07.040

CellPress

Neuron
Article

Structural Mechanism for Modulation of Synaptic Neuroligin-Neurexin Signaling by MDGA Proteins

Jonathan Elegheert,[1,*] Vedrana Cvetkovska,[2] Amber J. Clayton,[1,9] Christina Heroven,[1,3] Kristel M. Vennekens,[4,5] Samuel N. Smukowski,[6] Michael C. Regan,[7] Wanyi Jia,[1,10] Alexandra C. Smith,[8] Hiro Furukawa,[7] Jeffrey N. Savas,[6] Joris de Wit,[4,5] Jo Begbie,[8] Ann Marie Craig,[2,*] and A. Radu Aricescu[1,3,11,*]
[1]Division of Structural Biology, Wellcome Trust Centre for Human Genetics, University of Oxford, Roosevelt Drive, Oxford OX3 7BN, UK
[2]Djavad Mowafaghian Centre for Brain Health and Department of Psychiatry, University of British Columbia, Vancouver, BC V6T 2B5, Canada
[3]MRC Laboratory of Molecular Biology, Francis Crick Avenue, Cambridge Biomedical Campus, Cambridge CB2 0QH, UK
[4]VIB Center for Brain and Disease Research, Herestraat 49, B-3000 Leuven, Belgium
[5]Department of Neurosciences, KU Leuven, Herestraat 49, B-3000 Leuven, Belgium
[6]Department of Neurology, Feinberg School of Medicine, Northwestern University, Chicago, IL 60611, USA
[7]Keck Structural Biology Laboratory, Cold Spring Harbor Laboratory, Cold Spring Harbor, New York, USA
[8]Department of Physiology, Anatomy and Genetics, University of Oxford, South Parks Road, Oxford OX1 3QX, UK
[9]Present Address: Adaptimmune, 60 Jubilee Avenue, Milton Park, Abingdon OX14 4RX, UK
[10]Present Address: Wellcome Trust-Medical Research Council Cambridge Stem Cell Institute, University of Cambridge, Cambridge, UK
[11]Lead Contact
*Correspondence: jelegheert@strubi.ox.ac.uk (J.E.), acraig@mail.ubc.ca (A.M.C.), radu@mrc-lmb.cam.ac.uk (A.R.A.)
http://dx.doi.org/10.1016/j.neuron.2017.07.040

SUMMARY

Neuroligin-neurexin (NL-NRX) complexes are fundamental synaptic organizers in the central nervous system. An accurate spatial and temporal control of NL-NRX signaling is crucial to balance excitatory and inhibitory neurotransmission, and perturbations are linked with neurodevelopmental and psychiatric disorders. MDGA proteins bind NLs and control their function and interaction with NRXs via unknown mechanisms. Here, we report crystal structures of MDGA1, the NL1-MDGA1 complex, and a spliced NL1 isoform. Two large, multi-domain MDGA molecules fold into rigid triangular structures, cradling a dimeric NL to prevent NRX binding. Structural analyses guided the discovery of a broad, splicing-modulated interaction network between MDGA and NL family members and helped rationalize the impact of autism-linked mutations. We demonstrate that expression levels largely determine whether MDGAs act selectively or suppress the synapse organizing function of multiple NLs. These results illustrate a potentially brain-wide regulatory mechanism for NL-NRX signaling modulation.

INTRODUCTION

Cell-surface synaptic organizing proteins play a central role in the assembly, maturation, stabilization, and plasticity of neuronal synapses (Siddiqui and Craig, 2011). Members of the presynaptic neurexin (NRX) and postsynaptic neuroligin (NL) transmembrane protein families form the axis of a signaling pathway that is crucial for the formation and function of excitatory and inhibitory synapses throughout the brain (Südhof, 2008). The NL-NRX complexes promote synaptic cell adhesion via direct extracellular interactions and recruit the molecular machinery for neurotransmitter release and reception. NLs recruit ionotropic glutamate and $GABA_A$ receptors through direct interactions or using DLG (Discs large) family or gephyrin and collybistin accessory proteins, respectively (Bemben et al., 2015). NRXs interact intracellularly with CASK and Mint PDZ domain proteins and the synaptic vesicle protein synaptotagmin; α-NRXs also functionally link to presynaptic voltage-gated Ca^{2+} channels (Reissner et al., 2013).

NLs are generated from five genes in humans or four genes in mice, and further diversified by two sites of alternative splicing: spliced sequences A (SSA) and B (SSB). Mammalian NRXs show even greater diversity: over a thousand variants are generated from three genes, two promoters (α and β), and six sites of alternative splicing (SS1–6) (Schreiner et al., 2014; Ullrich et al., 1995). The extracellular region of the NLs contains a cholinesterase-like domain that forms a stable interaction with the α/β-NRX1-3 LNS6 (laminin, NRX, sex-hormone-binding globulin) domain (Araç et al., 2007; Chen et al., 2008; Fabrichny et al., 2007). NL1(+B) binds only β-NRXs (Boucard et al., 2005) and functions at glutamatergic synapses (Song et al., 1999), while NL2 binds all NRXs and functions at GABAergic synapses (Graf et al., 2004; Varoqueaux et al., 2004).

Besides NLs, the various NRXs bind a multitude of postsynaptic protein families to organize synapses: leucine-rich repeat transmembrane proteins (LRRTMs), calsyntenin 3, dystroglycan, latrophilin 1, cerebellins (reviewed in de Wit and Ghosh, 2016), and recently, C1q-like proteins (Matsuda et al., 2016). Molecular interactions are controlled by NRX promoter usage and splicing. For example, introduction of the 30-residue SS4 into β-NRX1 substantially weakens the NL-NRX1 interaction (Koehnke et al., 2010), abolishes the LRRTM1-2-NRX1 interaction (Siddiqui et al., 2010), and directs β-NRX1 into the cerebellin pathway (Elegheert et al., 2016; Uemura et al., 2010). Likewise, alternative binding partners for NL have been recognized. Thrombospondin

 © 2017 MRC Laboratory of Molecular Biology. Published by Elsevier Inc.
This is an open access article under the CC BY license (http://creativecommons.org/licenses/by/4.0/).

1 (TSP1) (Xu et al., 2010) and the NMDA receptor (NMDAR) (Budreck et al., 2013) both bind NL1, and the astrocyte-secreted protein hevin bridges NL1 and α-NRX (Singh et al., 2016) to promote glutamatergic synaptogenesis.

In contrast to all these positive effectors and modulators, the discovery of the Ig superfamily (IgSF) MDGA (meprin, A-5 protein, and receptor protein-tyrosine phosphatase mu [MAM] domain-containing glycosylphosphatidylinositol anchor) proteins as negative modulators of NL is remarkable. MDGA1 was found to block the interaction of NL2 with NRX and suppress inhibitory synapse development in cultured neurons (Pettem et al., 2013), while MDGA2 blocks the interaction of NL1 and NL2 with NRX and can suppress excitatory and inhibitory synapse development (Connor et al., 2016). MDGA proteins are attached to the postsynaptic membrane via a C-terminal GPI anchor, and their large (~900 amino acids) extracellular domain consists of six immunoglobulin-like domains ($Ig_{1\text{-}6}$), a fibronectin type III-like ($FnIII_7$) domain, and a memprin, A5, mu (MAM_8) domain.

Aberrant signaling in the NL-NRX pathway is strongly linked to autism spectrum disorders (ASDs) and schizophrenia (Südhof, 2008). Similarly, intronic SNPs in *MDGA1* are linked to schizophrenia (Kähler et al., 2008; Li et al., 2011), and *MDGA2* loss-of-function truncations were found in unrelated cases of ASD (Bucan et al., 2009). Single-allele knockout of the *Mdga2* gene in mice elevated both excitatory neurotransmission and functional connectivity and produced behavioral phenotypes related to ASD (Connor et al., 2016). *Mdga2* haploinsufficiency phenotypes were associated with elevated levels of NL1 and DLG family proteins and proposed to be due to diminished block of NL1-NRX signaling (Connor et al., 2016). However, based on a novel synaptic cleft tagging strategy in cell culture, another recent study proposed a role for MDGA2 selectively at inhibitory synapses and MDGA1 at excitatory synapses (Loh et al., 2016), raising controversy about the precise functions of MDGAs and revealing a need for more in-depth comprehensive analyses.

Despite the recent focus on mapping the complex molecular landscape of NL-NRX signaling modulators, a structural and mechanistic understanding of these processes is still lacking. In this study, we present the crystal structure of the near-complete MDGA1 extracellular domain and that of its prototypical complex with NL1, providing detailed insight into the structural basis of the modulation of NL-NRX signaling by MDGA proteins. We show that human MDGA1 and MDGA2 have the ability to interact with human NL1–5, thereby extending the previously proposed restricted, binary NL-MDGA interaction code (Connor et al., 2016; Lee et al., 2013; Pettem et al., 2013). Furthermore, we demonstrate that MDGA1 and MDGA2 are able to broadly block NL synaptogenic activity in a concentration- and splice insert-dependent fashion. Given the broad distribution of MDGA and NL-NRX complexes, our work provides a framework for understanding potential brain-wide modulation of NL-NRX signaling by MDGA proteins.

RESULTS

Crystal and Solution Structure of MDGA1

As a first step toward solving the structure of an NL-MDGA complex, we targeted the full-length apo MDGA1 extracellular domain for crystallization. Following an extensive screen of constructs from various species, we obtained diffraction-quality crystals and solved the structure of the complete chicken MDGA1 extracellular region ($cMDGA1_{ECTO}$; Ig_1-Mam_8; Gln19-Lys919; 79.5% sequence identity and 88.4% sequence similarity with human $MDGA1_{ECTO}$; Figure S1) using selenomethionine single-wavelength anomalous diffraction (Se-SAD) at 3.20 Å (Figures 1A and 1B; Table S1). $cMDGA1_{ECTO}$ was treated with endoglycosidase F1 (Endo F1) prior to crystallization, leaving a single N-linked *N*-acetylglucosamine monosaccharide on glycosylated Asn residues after enzymatic cleavage. Seven domains ($Ig_{1\text{-}6}$ to $FnIII_7$) could be unequivocally resolved in the electron density maps; however, the C-terminal MAM_8 domain was not visible and most likely highly mobile and accommodated in the solvent channels of the crystal. The $cMDGA1_{ECTO}$ $Ig_{1\text{-}6}$-$FnIII_7$ domains form a surprisingly compact, folded structure that is ~120 Å wide, ~110 Å high, and ~50 Å deep, fitting comfortably within the typical height of the synaptic cleft (~20–25 nm). Its approximately triangular shape, unique among the cell-surface receptors crystallized to date, is a consequence of sharp-angled Ig_2-Ig_3, Ig_4-Ig_5, and Ig_6-$FnIII_7$ inter-domain linkers that are stabilized by numerous inter-domain contacts.

The Ig_2-Ig_3 domain contacts (341 $Å^2$ buried surface area [BSA]) are formed between (1) the Ig_2 β strands βA and βG and (2) the loop structure connecting Ig_2 and Ig_3, and also Ig_3 loops *BC* and *FG*. The Ig_4-Ig_5 domain contacts (598 $Å^2$ BSA) are formed between (1) the Ig_4 β strand βA and loop *AB* and (2) Ig_5 loops *BC* and *FG*. The Ig_6-$FnIII_7$ domain contacts (396 $Å^2$ BSA) are formed between (1) the Ig6 β strand βA′ and loops *A′B* and *EF* and (2) the loop connecting Ig_6 and $FnIII_7$ and $FnIII_7$ loops *BC* and *FG*. Finally, the Ig_1-$FnIII_7$ domain contacts (395 $Å^2$ BSA) close the $cMDGA1_{ECTO}$ triangle and are formed between (1) the Ig_1 N-terminal stretch (Gln19-Tyr22) and loop *BC* and (2) $FnIII_7$ loops *AA′* and *C′E*, and β strands βA and βB (Figure 1C). The linear orientation of Ig_1 and Ig_2 is stabilized by a disulfide bond, distinct from the core Ig domain disulfide bonds, between Cys36 located on Ig_1 loop *AA′* and Cys222 located on Ig_2 loop *FG* (Figures 1A and 1B).

In the crystal, two MDGA molecules form an unexpected intertwined dimeric arrangement with individual C-terminal ends pointing in opposite directions (Figure S2A). Homophilic interfaces are formed between domain pairs Ig_1-Ig_5*, Ig_2-Ig_2*, and Ig_6-$FnIII_7$* (where * denotes contributions from the second MDGA monomer); their combined BSA is 2,666 $Å^2$, suggesting a stable association. Interestingly, this arrangement is compatible with both a potential *cis*- or *trans*-homophilic interaction and might indicate formation of an adhesive or self-inhibitory complex (Figure S2B). Recombinantly expressed MDGA1 targets to axons and dendrites and partially co-localizes with inhibitory and excitatory postsynaptic markers in cultured hippocampal rodent neurons (Loh et al., 2016; Pettem et al., 2013). Native MDGA1 and MDGA2 were observed in axon tracts in chicken (Fujimura et al., 2006) and zebrafish (Ingold et al., 2015), and a putative *trans*-homophilic interaction of MDGA2 was proposed to function in directed axonal growth (Joset et al., 2011).

To investigate the dimerization potential of the MDGA1 extracellular region in solution, we pursued multiple experimental avenues. First, we determined the $cMDGA1_{ECTO}$ solution structure

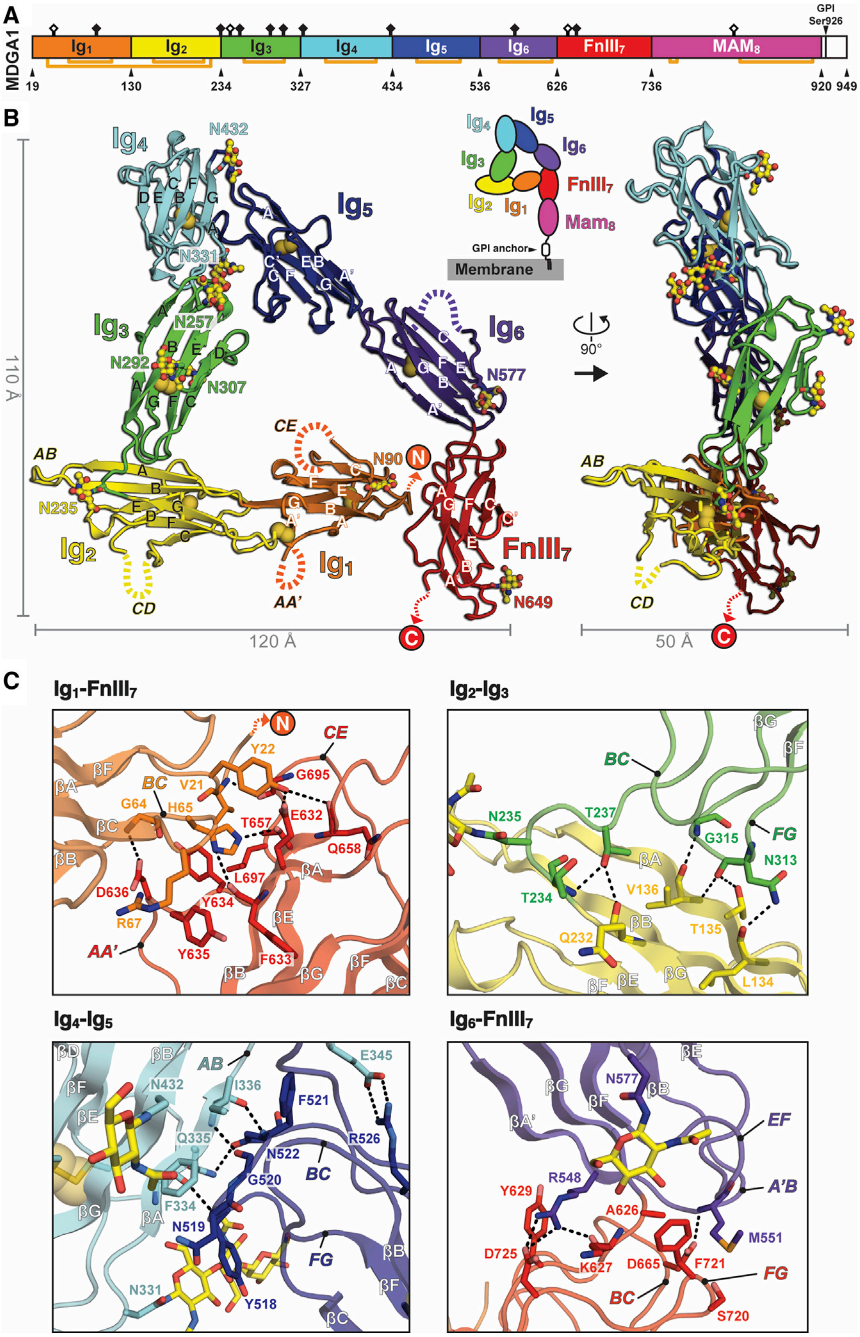

Figure 1. Crystal Structure of MDGA1

(A) Schematic representation of the chicken MDGA1 (cMDGA1) domain structure. Gln19-Lys919, spanning Ig_1-Mam_8, was used for structure determination. Black diamonds indicate Asn residues with crystallographically confirmed N-linked glycosylation (nine positions). Open diamonds indicate Asn residues with predicted but crystallographically unconfirmed N-linked glycosylation (four positions). Orange lines connect cysteine residues engaged in disulfide bonds.

(B) Crystal structure of $cMDGA1_{ECTO}$. Disulfide bridges are shown as yellow spheres. Glycan moieties visible in the electron density maps are shown in ball and stick representation. N and C termini, β strands, and selected Ig_{1-2} loop structures are annotated to the structure. The MAM_8 domain was not visible in the electron density maps, probably due to a flexible $FnIII_7$-MAM_8 linker.

(C) Details of the $cMDGA1_{ECTO}$ Ig_1-$FnIII_7$, Ig_2-Ig_3, Ig_4-Ig_5, and Ig_6-$FnIII_7$ domain contacts. Putative hydrogen bonds and hydrophilic interactions are indicated with black dashed lines.

See also Figures S1 and S2.

using small-angle X-ray scattering (SAXS) at a concentration of 30 μM. The scattering data were unambiguously incompatible with a dimeric MDGA1 molecule but were instead accurately (χ^2 = 1.17) modeled as a limited ensemble of monomeric conformers with pronounced flexibility at the $FnIII_7$-Mam_8 domain linkage (Figure S2C). In accordance with our SAXS data, we determined using analytical ultracentrifugation (AUC) that human $MDGA1_{ECTO}$ is monomeric at a concentration of 60 μM (Figures S2D and S2E). Finally, to probe whether potential MDGA1 self-association might instead be transient, we performed surface plasmon resonance (SPR) experiments in which wild-type $cMDGA1_{ECTO}$ was compared with a negative control mutant that contained three N-linked glycans inserted at distinct homophilic interfaces (Arg156Asn in Ig_2, Ser502Asn in Ig_5, and Arg680Asn in $FnIII_7$) for binding to wild-type $cMDGA1_{ECTO}$. Both $cMDGA1_{ECTO}$ variants failed to interact with wild-type $cMDGA1_{ECTO}$ up to a concentration of 100 μM (Figure S2F), indicating that no homophilic $cMDGA1_{ECTO}$ interactions occurred. Together, our results provide no biochemical evidence for an MDGA1 *cis*- or *trans*-homophilic dimer, and we propose that opening of the triangular $cMDGA1_{ECTO}$ structure by transient disruption of the limited Ig_1-$FnIII_7$ interface allowed formation of the dimeric arrangement in the crystal lattice.

Crystal Structure of an NL-MDGA Complex

We performed an extensive crystallization screening of the NL-MDGA complexes formed between $MDGA1\text{-}2_{ECTO}$ and $NL1\text{-}2_{ECTO}$ constructs from various species, and succeeded in generating diffraction-quality crystals and determining the structure of the Endo F1-treated complex formed between $cMDGA1_{ECTO}$ and the human NL1 cholinesterase domain lacking splice inserts ($hNL1_{ECTO}$; Gln46-Asp635; Figure S3) at 3.30 Å (Figures 2A and 2B; Table S1). The $hNL1_{ECTO}$-$cMDGA1_{ECTO}$ complex has a 2:2 stoichiometry and overall dimensions of ~180 Å wide, ~110 Å high, and ~120 Å deep. Two MDGA1 monomers flank the NL1 dimer to form a 2-fold symmetric complex. Remarkably, the overall root-mean-square deviation (RMSD)

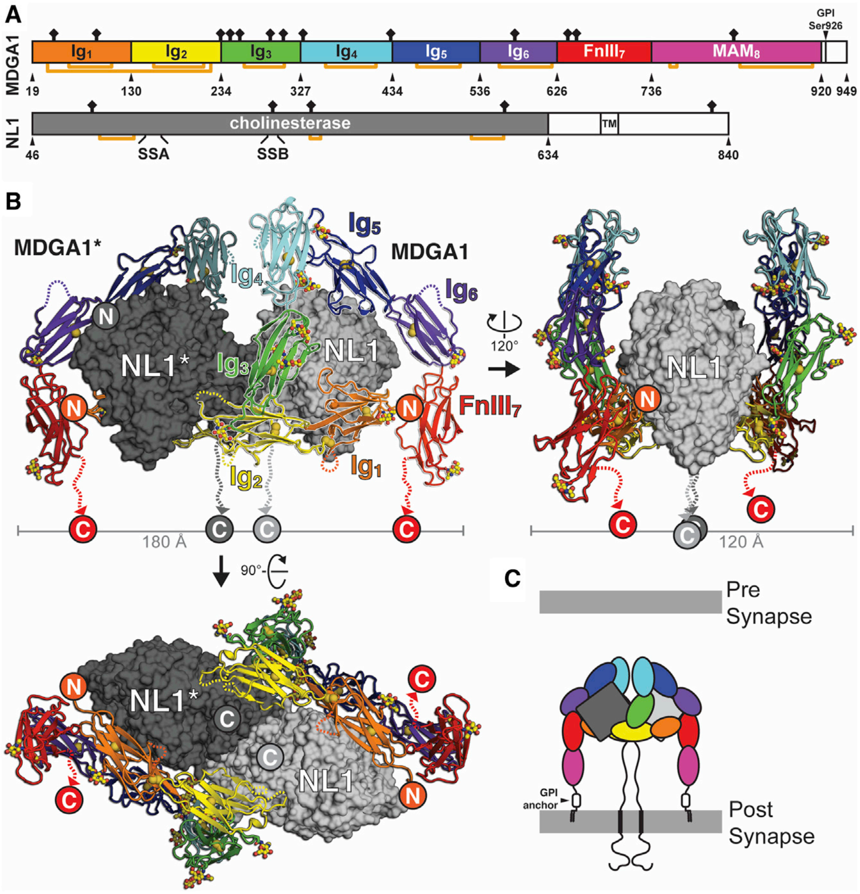

Figure 2. Crystal Structure of an NL-MDGA Complex

(A) Schematic representation of the constructs used for co-crystallization of the hNL1(–A–B)$_{ECTO}$-cMDGA1$_{ECTO}$ complex. Orange lines connect cysteine residues engaged in disulfide bonds. SSA and SSB depict the position of spliced sequences A and B on NL1, respectively. The MDGA1 Mam$_8$ domain was included in the crystallization construct but was not observed in the electron density, similar to the free cMDGA1$_{ECTO}$ structure.

(B) Front, 120° rotated side, and 90° rotated bottom views of the hNL1(–A–B)$_{ECTO}$-cMDGA1$_{ECTO}$ complex, shown in surface (NL1) and cartoon (MDGA1) representation. Disulfide bridges are shown as yellow spheres. Glycan moieties visible in the electron density maps are shown in ball and stick representation. The C termini of MDGA1 and NL1 point in the same direction, suggesting a complex formed in *cis*, located on the postsynaptic membrane.

(C) Schematic representation of the postsynaptic NL1-MDGA1 *cis* complex.

See also Figure S3.

between apo and NL1-bound cMDGA1$_{ECTO}$ structures is only 1.5 Å over 647 Cα atoms, underlining the stability and importance of this unusual multi-domain architecture. The NL1 and MDGA1 C termini point in the same direction and thus confirm an interaction in *cis*, situated on the postsynaptic membrane (Figures 2B and 2C). Each MDGA1 molecule spans the NL1 dimer using two large, separate interaction sites located on both NL1 monomers (Sites I and II) (Figure 3A). The Ig$_{1-3}$ domains mediate all MDGA1 contacts, consistent with previous domain-deletion experiments (Pettem et al., 2013). In contrast with the NL-NRX complex (Araç et al., 2007; Chen et al., 2008; Fabrichny et al., 2007), there was no evidence for the presence of coordinated calcium atoms at either Site I or II interfaces.

The numbering scheme employed in all following structural analyses is based on UniProt: P58400 (human β-NRX1), Q0WYX8 (chicken MDGA1), and Q8N2Q7 (human NL1). Annotation of secondary structural elements follows the acetylcholinesterase (AChE) nomenclature (Fabrichny et al., 2007).

The smaller Site I (859 Å^2 BSA) is formed between residues from (1) MDGA1$_{Ig1}$ β strands C, F, and G and loop *CE* and (2) NL1 loops Leu289-Gln307, Ile377-Asp385 (part of "loop L1"), Gln392-Tyr398, and Phe496-Pro499 and helices α2(4,5) and α4(6,7) (Figure 3B). His$_{NL1}$291, Tyr$_{NL1}$292, Asp$_{NL1}$384, and Glu$_{NL1}$394 are at the core of Site I. His$_{NL1}$291 and Tyr$_{NL1}$292 make Van der Waals (VdW) contacts and form putative hydrogen bonds with multiple MDGA1$_{Ig1}$ residues. Asp$_{NL1}$384 and Glu$_{NL1}$394 form putative salt bridges with Arg$_{MDGA1}$105 and Arg$_{MDGA1}$123 (Figure S4A).

The larger Site II (1,000 Å^2 BSA) is formed between residues from (2) MDGA1$_{Ig2}$ β strands A, B, D, and E and NL1 α helices α2(7,8) and α3(7,8); (2) MDGA1$_{Ig2}$ loop AB_{Ig2} and NL1 loops Ala110-Pro132 ("Cys loop") and Asp361-Asp385 ("loop L1"); and (3) peripheral interactions contributed by MDGA1$_{Ig3}$ to NL1 α helix α2(7,8) and loop Val417-Ser424 (Figure 3B). Notably, MDGA1 loops AB_{Ig2} and DE_{Ig2} form long protrusions that give Ig$_2$ a concave shape to accommodate the NL1 α helix α2(7,8) (Figure 3B). The Phe$_{NL1}$430-Phe$_{MDGA1}$154 π-π sandwich stacking interaction is central to this interface and is lined by multiple hydrogen-bonding and charged interactions. The tip of MDGA1 loop AB_{Ig2} extends into a pocket lined predominantly by hydrophobic NL1 residues. Part of loop AB_{Ig2} (Ile140-Ser146 stretch) could not be resolved in the complex electron density map (Figure S4A).

The NL1 "Cys loop" (part of loop Ala110-Pro132) and "loop L1" (part of loop Asp361-Asp385) occlude the "gorge" that, in AChE, leads to the enzyme active site. Interestingly, these loop structures form an integral part of the NL-MDGA interface. In this sense, MDGA resembles the snake toxin fasciculin (Fas) for binding to AChE (Bourne et al., 1995; Harel et al., 1995). There are, however, no indications that Fas might bind NL and interfere with MDGA binding.

The function of the NL Leu449-Arg450-Glu451 (LRE) adhesion motif, conserved in all NLs and located in the α3(7,8) helix (Figures 7B and S3C), is not clear. The LRE motif was first identified in the extracellular matrix protein laminin β2, where it is involved in binding the Ca$_V$2.2 voltage-gated

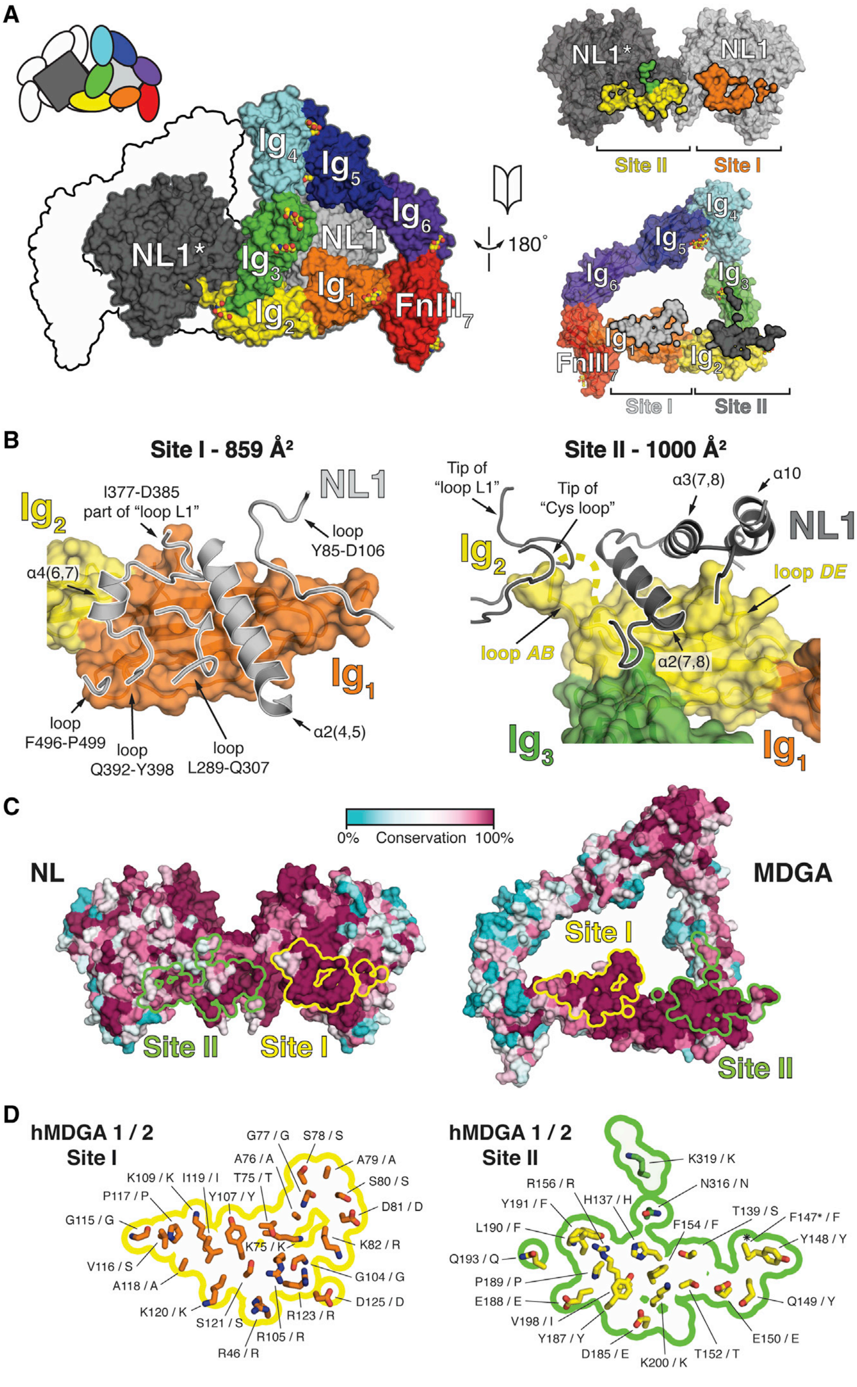

Figure 3. Details and Conservation of the NL-MDGA Site I and II Interfaces

(A) 180° rotated open book view of the NL1-MDGA1 Site I and Site II interaction interfaces. Site I (859 Å^2 buried surface area [BSA]) and Site II (859 Å^2 BSA) group interactions contributed by MDGA1 Ig_1 and Ig_2-Ig_3, respectively.

(B) Overview of the NL1 secondary structure elements contacted by $MDGA_{Ig1}$ to form Site I, and $MDGA_{Ig2-3}$ to form Site II.

(C) View of the NL1 and MDGA1 interaction interfaces, color-coded by sequence conservation in vertebrate NL1, NL2, NL3, NL4, and NL5 (1,046 total sequences), and vertebrate MDGA1 and MDGA2 (420 total sequences).

(D) View of the MDGA1 interaction interface. Site I and Site II interfaces are outlined by yellow and green lines, respectively. Per residue position, equivalent residues in human MDGA1 and MDGA2 are annotated to highlight overall sequence conservation of the interaction interfaces. Star symbols (*) indicate residues for which side chain electron density was not clearly discernable.

See also Figure S4.

calcium channel. Furthermore, the LRE motif is present in the majority of mammalian AChEs, and besides in NL, it is also observed in the cholinesterase-like adhesion molecules neurotactin and glutactin (Johnson and Moore, 2013). Both Arg450 and Glu451 form an integral part of the NL-MDGA interface and interact with Tyr187 and Leu190, respectively, on MDGA1 loop DE_{Ig2} (Figures 7A and S4A), offering a first functional role for this LRE-tripeptide in NLs.

Sequence conservation analysis indicated that both Site I and Site II interfaces are highly conserved in vertebrate MDGAs and NLs (Figures 3C, 3D, and S4B); this observation strongly points toward a common binding mode between all MDGA and NL family members.

We mapped all predicted N-glycosylation sites for human MDGA1-2 and NL1-5 (NLs lacking splice inserts) on the $cMDGA1_{ECTO}$ and $hNL1_{ECTO}$ structures (Figures S4C and S4D). The MDGA1-specific N-glycan at Asn307, experimentally confirmed by identifying the corresponding *N*-acetylglucosamine monosaccharide in the $hNL1_{ECTO}$-$cMDGA1_{ECTO}$ electron density map, is the only glycan that is proximal to the binding interface and is situated in Ig_3 at the edge of Site II. Analysis of the complex structure, however, indicated that all putative N-linked glycans can project into the solvent, thereby avoiding interference with complex formation. Proteins for subsequent biophysical and cellular experiments were expressed in HEK293T and COS-7 cells, respectively, and were not deglycosylated.

MDGA and NRX Share Binding Interfaces on NL

We compared our NL1-MDGA1 structure with previously reported NL1-β-NRX1 complexes (Araç et al., 2007; Chen et al., 2008; Fabrichny et al., 2007). Using the highest resolution NL1-β-NRX1 structure available (PDB: 3B3Q; 2.4 Å; Chen et al., 2008), both complexes align with an RMSD of 0.292 Å over

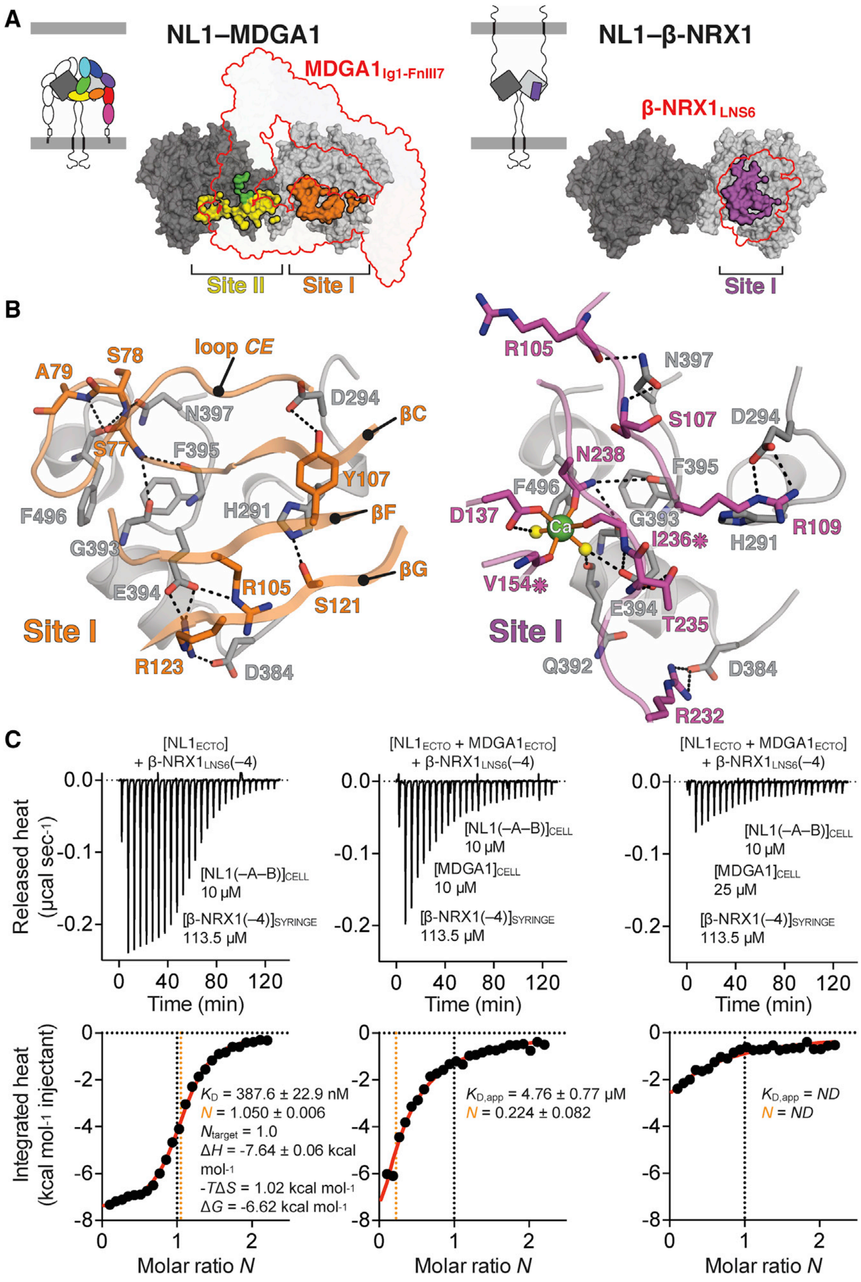

Figure 4. MDGA and NRX Compete for Binding to the NL Site I Interface

(A) Comparison of the NL1-MDGA1 and NL1-β-NRX1 complex binding modes. The NL1-MDGA1 and NL1-β-NRX1 (based on PDB: 3B3Q; Chen et al., 2008) interfaces are oriented similarly, based on structural alignment of one NL1 monomer (0.292 Å RMSD over 453 NL1 Cα positions). The respective molecular footprints of MDGA1 and β-NRX1 are outlined with a red stroke. The NL1-MDGA1 Site I and Site II interfaces, and the NL1-β-NRX1 Site I interface, are shown in surface representation.

(B) Detailed comparison of the core NL1 residues shared between NL1-MDGA1 and NL1-β-NRX1 Site I interfaces. Putative hydrogen bonds and hydrophilic interactions are indicated with black dashed lines. The hexadentate coordination shell of the NL1-β-NRX1 interface calcium (Ca) atom is indicated with solid orange lines.

(C) Summary of the calorimetric competition assay binding isotherms, indicating that $MDGA1_{ECTO}$ can compete with β-$NRX1_{LNS6}$(–4) for binding to $NL1_{ECTO}$ in a concentration-dependent fashion. In each case, the experimental geometry is "[cell contents] + syringe contents." For calculation of the stoichiometry, NL1, MDGA1, and β-NRX1(–4) were considered in their monomeric state. Thermodynamic binding parameters are annotated. *ND*, not determined. An ~2.5-fold molar excess of MDGA1 was required to fully block binding of β-$NRX1_{LNS6}$(–4) to $NL1_{ECTO}$.

453 NL1 Cα positions. Strikingly, Site I overlaps nearly completely with the NL1-β-NRX1 interface, suggesting that MDGA prevents the NL-NRX interaction via steric hindrance (Figure 4A). Core NL1 residues shared between NL1-MDGA1 and NL1-β-NRX1 interfaces are His291, Asp294, Asp384, Gly393-Asn397, Phe496, and Gly497. $Arg_{MDGA1}123$ mimics $Arg_{\beta\text{-}NRX1}232$ for binding to $Asp_{NL1}384$. $Arg_{MDGA1}123$ and $Arg_{MDGA1}105$ engage $Glu_{NL1}394$ in ionic interactions, whereas in NL1-β-NRX1, the latter residue contacts $Thr_{\beta\text{-}NRX1}235$ and is part of the hexadentate coordination shell of the obligate interface calcium atom. $Asp_{NL1}294$ forms a hydrogen bond with $Tyr_{MDGA1}107$, whereas it forms a bifurcated ionic interaction with $Arg_{\beta\text{-}NRX1}109$ in NL1-β-NRX1. Finally, NL1 residues Gly393, Phe395, Phe496, and Asn397 are contacted by $MDGA1_{Ig1}$ loop *CE*, preventing their network of hydrogen-bonded interactions with β-NRX1 residues (Figure 4B).

We set up an isothermal titration calorimetry (ITC) assay to investigate whether $MDGA1_{ECTO}$ competes with β-$NRX1_{LNS6}$ lacking SS4 (β-$NRX1_{LNS6}$(–4)) for binding to $NL1_{ECTO}$. Titration of β-$NRX1_{LNS6}$(–4) into $NL1_{ECTO}$ alone revealed a strong exothermic interaction and a K_D of ~390 nM. Application of an equimolar amount of $MDGA1_{ECTO}$ to $NL1_{ECTO}$ in the titration cell did not fully block the $NL1_{ECTO}$-β-$NRX1_{LNS6}$(–4) interaction, but decreased its apparent K_D ($K_{D,app}$) ~12-fold to 4.76 μM. Application of a 2.5-fold molar excess of $MDGA1_{ECTO}$ over $NL1_{ECTO}$ was required to fully block binding of β-$NRX1_{LNS6}$(–4) to $NL1_{ECTO}$ (Figure 4C). These results are consistent with the notion that MDGA is not an ultra-high-affinity decoy receptor, and that by varying the levels of MDGA, the level of NL-NRX complex formation can be tuned.

MDGA1 and MDGA2 Bind All NL Isoforms

We hypothesized that the interactions between human NLs and MDGAs are not limited to certain pairs of isoforms, given the high level of conservation of the Site I and Site II interface residues

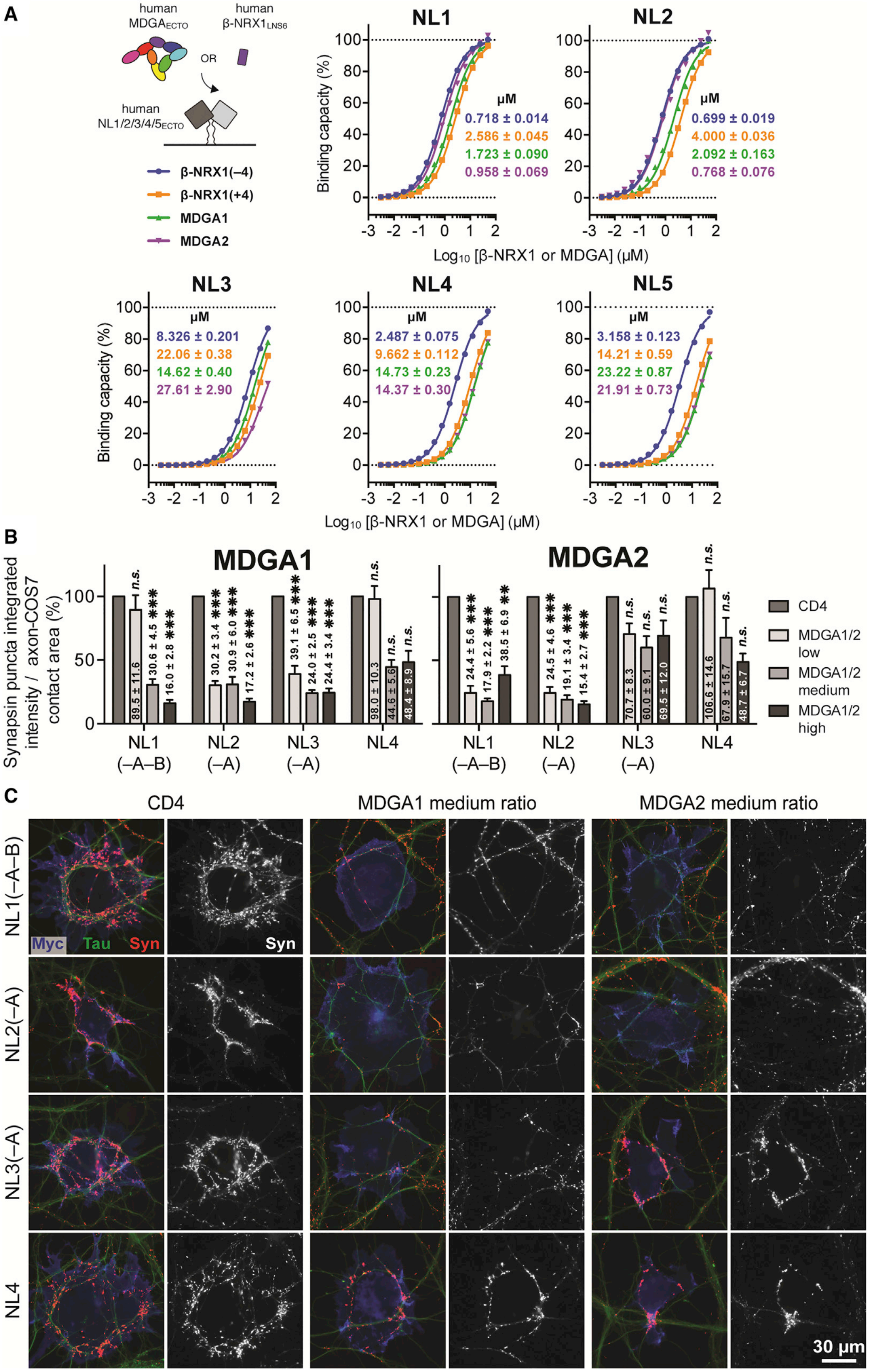

(legend on next page)

among human NL1–5 and MDGA1-2 (Figures 3C and S4B). To test this, we determined the binding strengths of all pairwise NL-MDGA ectodomain interactions using SPR. We initially focused on the unspliced NL variants for these interaction studies. As a control, we measured the pairwise interactions between NL1–5_{ECTO} and β-$NRX1_{LNS6}$ with and without SS4 (β-$NRX1_{LNS6}$(±4)). The reference interaction of $NL1_{ECTO}$ with β-$NRX1_{LNS6}$(–4) showed an approximately 2-fold higher equilibrium dissociation constant (K_D) than the one derived from ITC (K_D of 718 ± 14 nM versus 388 ± 23 nM, respectively; Figures 4C and 5A).

Overall, our measurements revealed K_Ds for NL-MDGA in the high nanomolar (nM) to low micromolar (μM) range, similar to NL-β-NRX1(±4) (Figure 5A). Accordingly, MDGA does not appear to be an ultra-high-affinity decoy receptor for NL. MDGA1 and MDGA2 interacted most strongly with NL1 and NL2, and MDGA2 binds NL1 and NL2 2-fold stronger than MDGA1 (K_D of ~1 and ~2 μM, respectively). Interaction affinities of $MDGA2_{ECTO}$ and β-$NRX1_{LNS6}$(–4) for NL1–2_{ECTO} are nearly identical. Interestingly, both MDGA1 and MDGA2 interacted ~10- to ~20-fold weaker with NL3, NL4, and NL5 (K_D of ~15–25 μM). Whereas $NL3_{ECTO}$ also binds β-$NRX1_{LNS6}$(–4) with low affinity (K_D of ~8.5 μM), $NL4_{ECTO}$ and $NL5_{ECTO}$ still bind β-$NRX1_{LNS6}$ (–4) relatively strongly (K_D of ~2.5–3 μM), meaning that for NL4 and NL5, a larger discrepancy between binding strengths of β-NRX1(–4) and MDGA1-2 exists (Figure 5A).

Taken together, these experiments show (1) that MDGA1 and MDGA2 have the ability to interact with NLs that localize to excitatory glutamatergic (NL1 and NL3) (Budreck and Scheiffele, 2007; Song et al., 1999), inhibitory GABAergic (NL2 and NL3) (Budreck and Scheiffele, 2007; Graf et al., 2004; Varoqueaux et al., 2004), and inhibitory glycinergic (NL2 and NL4) (Hoon et al., 2011; Varoqueaux et al., 2004) synapses, and (2) that the subtle divergences in NL and MDGA amino acid composition (Figures 3D and S4B) may contribute to subtype preferences. Thus, our results extend the restricted, binary NL-MDGA code that was previously proposed (Connor et al., 2016; Lee et al., 2013; Pettem et al., 2013).

We sought to validate the interaction of MDGA1 and MDGA2 with multiple NLs. To this end, we fused the rat MDGA1 and MDGA2 ectodomains to the Fc region of human IgG. MDGA1- and MDGA2-Fc proteins were then used as bait to identify NLs in postnatal day 21 (P21) rat brain synaptosome extracts, using affinity chromatography coupled with mass spectrometry and bioinformatics analysis (Savas et al., 2014). For extraction, we used the detergent Triton X-100 at 1% w/v concentration. In two independent MDGA1-Fc pull-down experiments, we identified NL3, NL2, and NL1, ranked by spectral count (Figure S5B; Table S2). No peptides for NLs were detected in control experiments using Fc alone or using MDGA lacking Ig_{1-3} (MDGA1ΔIg1–3) (Table S2), demonstrating specificity in the assay. In two independent MDGA2-Fc pull-down experiments, we identified NL2 and, to a lesser extent, NL3 (Figure S5B). In the pull-downs, no NL4 or NL5 was detected; NL4 is of very low abundance (e.g., only ~3% of the total NL in mouse brain; Varoqueaux et al., 2006) and NL5 is restricted to humans. The pull-down results are consistent with our SPR data that indicated a stronger binding of NL3 to MDGA1 than to MDGA2 (Figure 5A).

MDGA1 and MDGA2 Modulate NL-Induced Recruitment of Hippocampal Synaptic Terminals

To assess whether MDGA1 and MDGA2 are able to broadly modulate NL-NRX-induced synapse formation, we set up a cellular hemi-synapse formation assay in which COS-7 cells co-expressing full-length (FL) N-terminally myc-tagged NL1–4 (myc-NL1–4_{FL}) and full-length N-terminally HA-tagged MDGA1-2 (HA-MDGA1-2_{FL}) variants were co-cultured with rat hippocampal neurons (Figures 5B, 5C, S6C, and S6D). These neurons express the –SS4 and +SS4 forms of all three α- and β-NRXs (α/β-NRX1-2-3) (Aoto et al., 2013). Accordingly, this assay integrates signals from multiple NRX isoforms, in contrast with our SPR or ITC assays, which only used β-NRX1(±4) as reference interactions (Figures 4C and 5A). To test our hypothesis that by varying the expression levels of MDGA1-2, the extent of NL-NRX complex formation and hence recruitment of synaptic terminals can be influenced, we tested three different plasmid ratios of MDGA1 and MDGA2. For MDGA1, low, medium, and high plasmid ratios designate a 2.2-, 3.5-, and 5.0-fold excess of plasmid DNA over NL, respectively. For MDGA2, these ratios were chosen to be 1.5-fold higher to achieve similar surface protein levels as MDGA1 (Figure S6A). The low ratios used here were similar to the ratios used in our previous co-culture assays of rodent MDGA1-2 with NL1 and NL2 (Connor et al., 2016; Pettem et al., 2013). Similar results were found here for human MDGA1-2 with NL1-2 (see Figures 5B and 8D, low ratio results). However, these earlier studies did not assess the effects of NL alternative splicing, varying ratios of MDGA to NL, or MDGA on NL3-4.

We observed here that MDGA1 and MDGA2 appeared to reduce the ability of all NLs to recruit presynaptic terminals, but with different potency. MDGA1 and MDGA2 both blocked NL1-induced recruitment of synaptic terminals, although a

Figure 5. MDGA1 and MDGA2 Bind All NL Isoforms and Suppress NL-Induced Recruitment of Synaptic Terminals in Co-culture

(A) Schematic representation of the SPR setup, summary of K_D values, and binding isotherms for the interaction of NL1–5_{ECTO} with MDGA1-2_{ECTO} and β-$NRX1_{LNS6}$(±4).

(B) COS-7 cells expressing myc-NL1–4 were co-transfected with HA-CD4 control, HA-MDGA1, or HA-MDGA2 and co-cultured with hippocampal neurons. The ability of the co-transfected cells to induce synapsin clustering was measured and normalized to the area of tau-positive axon contact. The bar graphs represent the mean of three independent experiments for low, medium, and high plasmid ratios (Figure S6A) of human HA-MDGA1-2:myc-NL1–4 (n > 24 total cells for each condition) with the CD4:myc-NL1–4 co-transfected controls normalized to 100% to show the relative change of synapsin integrated intensity at each ratio. Significance is shown for CD4 control versus MDGAs for each NL (one-way ANOVA with Bonferroni post hoc comparison). Error bars represent the SEM. *p < 0.05, **p < 0.01, ***p < 0.001; n.s., not significant. A detailed statistical quantification can be found in Table S3.

(C) Representative images of co-cultures immunostained for surface myc-NL (blue), surface HA-MDGA or CD4 control (data not shown), synapsin (red), and tau axonal marker (green). The isolated synapsin signal (white) is shown next to each color image. Scale bar, 30 μm.

See also Figures S5 and S6.

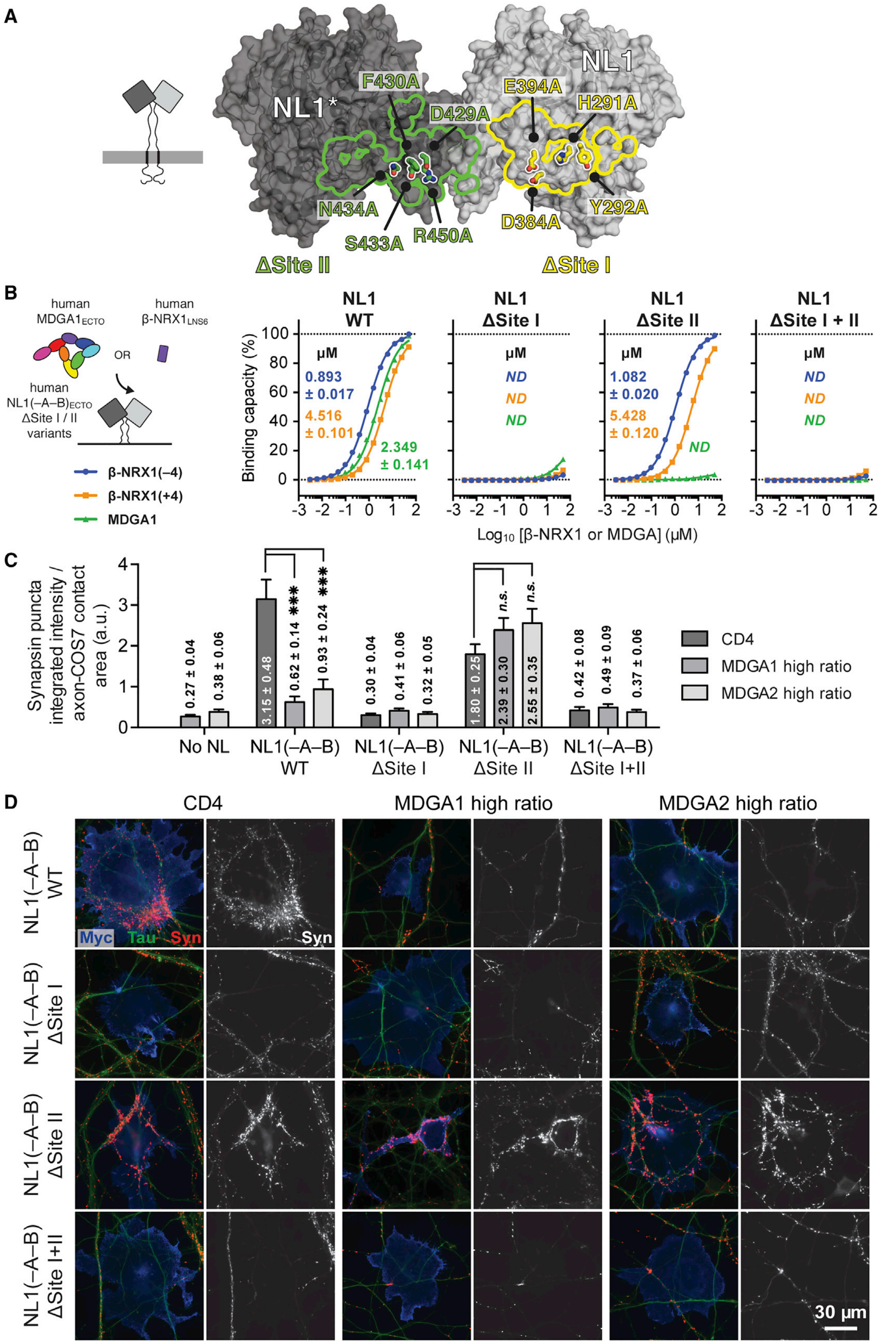

(legend on next page)

higher ratio was needed to obtain this effect for MDGA1 than for MDGA2 (Figure 5B; Table S3). Both MDGA1 and MDGA2 potently blocked NL2-induced recruitment of synaptic terminals. Thus, there was a weaker effect of MDGA1 on NL1 relative to NL2 activity in this neuron culture-based assay in comparison with similar binding seen with purified proteins in our equilibrium SPR experiments (Figure 5A). Differential effects in the co-culture were not due to any differences in surface levels of MDGAs or NLs (Figure S6B). We observed a stronger differential effect when evaluating NL3-induced synapse formation. Whereas MDGA1 was able to block recruitment of synaptic terminals at all ratios, MDGA2 was not. This is consistent with our SPR analysis, which derived lower responses and corresponding lower interaction affinities for the NL3-MDGA2 interaction (Figures 5A and S5A). Finally, both MDGA1 and MDGA2 were unable to significantly block NL4-induced synapse formation, although there was a trend toward suppression; this agrees with our SPR analysis that indicated that β-NRX1(–4) binds NL4 ~6-fold stronger than MDGA1-2. We suggest that even higher MDGA:NL plasmid ratios would be needed to fully block NRX binding. However, these conditions were not experimentally accessible in the assay format used, which imposed limits on the total amount of plasmid DNA that can be reliably transfected.

Overall, our results confirm that MDGA1 and MDGA2 can interfere with a broad range of NL-NRX interactions to modulate presynaptic differentiation. The functional outcome will ultimately be influenced by the relative abundances of all molecular players.

Assessment of Binding of NL1 with Hevin, Thrombospondin-1, and the NMDAR

Given that the interactions of thrombospondin 1 (TSP1) (Xu et al., 2010), hevin (Singh et al., 2016), and the NMDAR (Budreck et al., 2013) with NL1 are all dependent on the coupling of their respective extracellular domains, we hypothesized that MDGA might have the potential to also block binding of these proteins to NL, thereby assigning a more general inhibitory function to MDGA. To test this, we first set out to reproduce the interactions of NL1 with recombinant hevin, TSP1, and NMDAR using SPR. In our setup, secreted human hevin and TSP1 and detergent-solubilized rat NMDAR (GluN1a-GluN2B heterotetramer) (Karakas and Furukawa, 2014) were immobilized on the chip surface (Figure S7A). We found that, in contrast to the reference interaction of NL1(–A–B)$_{ECTO}$ with mouse α-NRX1$_{ECTO}$(–4), all three proteins failed to interact with NL1(–A–B)$_{ECTO}$ up to a concentration of 25 μM (Figures S7B and S7C).

Uncoupling of MDGA and NRX Binding to NL

Given that the NL-MDGA crystal structure revealed a composite Site I-II interface, whereas NL-NRX uses only Site I (Figure 4A), we hypothesized that NRX and MDGA binding can be uncoupled, i.e., NL can be rendered insensitive for modulation by MDGA by mutating the Site II interface. We introduced four core interface mutations into the NL1 Site I interface (NL1$^{\Delta Site\ I}$: His291Ala, Tyr292Ala, Asp384Ala, and Glu394Ala) and five into the Site II interface (NL1$^{\Delta Site\ II}$: Asp429Ala, Phe430Ala, Ser433Ala, Asn434Ala, and Arg450Ala) (Figure 6A). We opted to combine multiple mutations of key interface residues instead of using single-position alanine mutants to maximize our chances of obtaining a clear binding differential and cellular phenotype.

Consistent with both NL-MDGA and NL-β-NRX1 complex structures, we found using SPR that the ΔSite II mutant blocked MDGA1 binding but maintained binding of β-NRX1, whereas the ΔSite I and combined ΔSite I+II mutants fully abolished both β-NRX1 and MDGA1 interactions (Figures 6B and S8A).

Using the co-culture assay, we tested the impact of the ΔSite I and ΔSite II mutations on the recruitment of synaptic terminals by full-length NL1. Consistent with our SPR analysis, introduction of the NL1$^{\Delta Site\ I}$ and NL1$^{\Delta Site\ I+II}$ mutations, but not the NL1$^{\Delta Site\ II}$ mutations, prevented NL-NRX-induced synapse formation (Figures 6C and 6D; Table S4). Simultaneously, co-expression at high plasmid ratio of MDGA1 or MDGA2 with NL1 carrying the ΔSite II mutations did not lead to diminished recruitment of synaptic terminals (Figures 6C and 6D; Table S4). We concluded that the NL ΔSite II mutant selectively uncoupled NL-NRX binding and recruitment of synaptic terminals from inhibition by MDGA.

The ASD-Linked NL3 Mutation Arg451Cys Prevents Suppression of Synapse Formation by MDGA1

The well-characterized NL3 mutation Arg451Cys (R451C) leads to a number of ASD-linked phenotypes in mice (Tabuchi et al., 2007). In this knockin mouse model, R451C acts as a gain-of-function mutation by actually increasing inhibitory synaptic transmission, a result that is seemingly at odds with the severe reduction of NL3 in these mutant mice (Tabuchi et al., 2007). Indeed, complete knockout of NL3 has no such effect (Tabuchi et al., 2007).

Figure 6. Uncoupling of MDGA and NRX Binding to NL

(A) Annotation of the NL1 Site I (ΔSite I: H291A, Y292A, D384A, and E394A) and Site II (ΔSite II: D429A, F430A, S433A, N434A, and R450A) mutations.

(B) Schematic representation of the SPR setup, summary of K_D values, and binding isotherms for the interaction of wild-type and mutant human NL1(–A–B)$_{ECTO}$ with MDGA1-2$_{ECTO}$ and β-NRX1$_{LNS6}$(±4).

(C) COS-7 cells expressing myc-NLs were co-transfected with HA-CD4 control, HA-MDGA1, or HA-MDGA2 and co-cultured with hippocampal neurons. The ability of the co-transfected cells to induce synapsin clustering was measured and normalized to the area of tau-positive axon contact. The bar graphs represent the mean of three independent experiments for high plasmid ratios (Figure S6A) of human HA-MDGA1-2:myc-NL1 ($n > 22$ total cells for each ratio). Significance is shown for CD4 control versus MDGA1-2 for each NL1 variant (one-way ANOVA with Bonferroni post hoc comparison). Error bars represent the SEM. ***$p < 0.001$; n.s., not significant. Mutation of Site II renders NL1(–A–B) insensitive to suppression of synapse formation by MDGA1 and MDGA2. A detailed statistical quantification can be found in Table S4.

(D) Representative images of co-cultures immunostained for surface myc-NL (blue), surface HA-MDGA or CD4 control (data not shown), synapsin (red), and tau axonal marker (green). The isolated synapsin signal (white) is shown next to each color image. Scale bar, 30 μm.

See also Figure S8.

Our hNL1$_{ECTO}$-cMDGA1$_{ECTO}$ complex crystal structure shows that NL1 Arg450, which is equivalent to NL3 Arg451 and part of the NL1 Leu449-Arg450-Glu451 (LRE) motif (Figures 7B and S3C), is an integral part of the Site II interface (Figure 7A). We introduced the Arg450Cys (R450C) and Arg451Cys (R451C) mutations into NL1(–A–B)$_{ECTO}$ and NL3(–A)$_{ECTO}$, respectively. We observed diminished secretion for the mutants as compared to wild-type proteins (Figure S8B), consistent with reported trafficking defects and protein destabilization (Chih et al., 2004; Comoletti et al., 2004; Tabuchi et al., 2007). Using SPR, we then measured the interaction of β-NRX1(±4) and MDGA1-2 with these mutant proteins and compared them to the wild-type interactions. Our measurements revealed that for both NL1 and NL3, introduction of the R450/451C mutation nearly completely abolished binding of both MDGA1 and MDGA2, while leaving the binding of β-NRX1(±4) unaffected (Figures 7C and S8B). This is consistent with the fact that the R450/451C mutation is situated in the MDGA-specific Site II interface. In this sense, the mutation thus phenocopies our NL1 ΔSite II mutant (Figure 6B).

Using the co-culture assay, we tested the impact of the R451C mutation on the recruitment of synaptic terminals by full-length NL3. Importantly, although impaired relative to wild-type NL3, the R451C mutant can traffic to the surface of transfected COS-7 cells (Figure S8C) and rat hippocampal neurons (Figure S8D; consistent with Chih et al., 2004). Thus, for the co-culture analysis, we again selected COS-7 cells that displayed equal amounts of surface NL to ensure meaningful readout of synapse formation (Figure S8C). Consistent with our SPR analysis, introduction of the R451C mutation had no impact on NL-NRX-induced synapse formation when compared to wild-type NL3 (Figure 7D). Then, co-expression at low plasmid ratio of MDGA1, but not MDGA2, with NL3 wild-type led to diminished recruitment of synaptic terminals. This result closely reproduces our earlier observation (Figure 5B). Introduction of R451C, however, prevented the diminished recruitment of synaptic terminals mediated by MDGA1 (Figures 7D and 7E; Table S5). We concluded that R451C selectively uncoupled NL3-NRX binding and recruitment of synaptic terminals from inhibition by MDGA1.

Tuning of the NL-MDGA Interaction by NL SSA and SSB

Alternative splicing leads to insertion of SSA and SSB onto the NL cholinesterase scaffold. SSB is restricted to NL1, whereas distinct SSA sequences are present in NL1, NL2, and NL3. In NL1 and NL3, the two possible SSA sequences (A1 and A2) can also occur in tandem (denoted as A1A2) (Figure S3B). Whereas NL1 mRNAs containing and lacking splice insert A are detected at similar levels at hippocampal, cortical, and cerebellar excitatory synapses, mRNA coding for NL1(+B) is more abundant than for NL1(–B) (Chih et al., 2006). Simultaneously, the insertion point for SSB in NL1 is in close proximity to the Site I interface (Koehnke et al., 2010), suggesting that presence of SSB might affect MDGA binding. These observations prompted us to investigate the effect of insertion of SSA and SSB on the NL-MDGA complex formation. First, we mapped SSA, derived from a published NL1(+A1) crystal structure (PDB: 3VKF; Tanaka et al., 2012), onto the NL1-MDGA1 (0.399 Å RMSD over 477 NL1 Cα positions) and NL1-NRX1 (0.375 Å RMSD over 453 NL1 Cα positions) structures (Figure 8B).

Interestingly, although SSA is spatially distant from both Site I and Site II binding interfaces, it is in close proximity to the MDGA Ig$_5$ and Ig$_6$ domains (Figure 8B). As such, SSA might have the potential to either clash with Ig$_5$-Ig$_6$ or, conversely, provide an additional binding site for MDGA. We tested using SPR whether insertion of the distinct SSA sequences into NL1, NL2, or NL3 had an effect on the NL-MDGA or NL-NRX interactions. We were unable to detect a robust or meaningful impact of the SSA sequences on the binding strength of any NL1-3$_{ECTO}$-MDGA1-2$_{ECTO}$ or NL1-3$_{ECTO}$-β-NRX1$_{LNS6}$(±4) pair (Figures S9A, S9B, S10A, and S10B), suggesting that SSA possesses sufficient conformational freedom to not perturb the core NL-MDGA interaction. Accordingly, we suggest that SSA is not involved in modulating the NL-MDGA interaction.

Next, we determined the crystal structure of human NL1 containing SSB (hNL1(+B)) at 2.55 Å (Figure 8A; Table S1). The nine-residue SSB (NRWSNSTKG), inserted between Gly295 and Leu305, was clearly visible in the electron density; the N-linked glycan at Asn300, a modulator of the NL-NRX interaction (Chih et al., 2006; Comoletti et al., 2003), was, however, not fully resolved due to conformational flexibility (Figure 8A). Superposition of NL1(+B) and NL1-MDGA1 (0.308 Å RMSD over 434 NL1 Cα positions) or NL1-NRX1 (0.306 Å RMSD over 428 NL1 Cα positions) structures revealed that SSB is spatially immediately adjacent to both Site I interfaces (Figure 8B). We found, using SPR, that insertion of SSB weakened the NL1-MDGA1-2 interaction ~7-fold, while reducing the NL1-β-NRX1(±4) interaction less than 2-fold (Figure 8C). We propose that this differential effect is due to the much larger molecular footprint of MDGA and the resulting close proximity of the MDGA Ig$_5$ and Ig$_6$ domains to the N-linked glycan at Asn300, suggesting that SSB reduces the NL-MDGA interaction due to steric hindrance (Figure 8B). Indeed, removal of the N-linked glycan (Asn300Gln mutant) partially recovered the NL1-MDGA1-2 interaction affinity, whereas it had almost no effect on the NL1-β-NRX1(±4) interaction (Figure 8C).

Using the co-culture assay, we tested the effect of the presence of SSB on the ability of MDGA1-2 to block recruitment of synaptic terminals by full-length NL1(–B) and NL1(+B). Co-expression of MDGA1-2 at low, medium, and high plasmid ratios (as previously defined; Figure S6A) led to a decreased recruitment of terminals by both NL1(–B) and NL1(+B) (Figures 8D and 8E; Table S3); however, we found a concentration-dependent decrease for MDGA1. At the medium ratio, MDGA1 significantly blocked recruitment of terminals by NL1(–B), but not NL1(+B), consistent with the difference in binding observed in the SPR assay (Figure 8C). Taken together, our results suggest that, despite the proximity of SSB to the Site I interface, its presence does not eliminate the ability of MDGA to block NL-NRX signaling. Rather, SSB provides a way to fine-tune the NL1-MDGA1-2 interaction at excitatory synapses.

DISCUSSION

In this work, we present the structure of the near-complete MDGA1 extracellular domain and its complex with NL1, establishing the general recognition paradigm between these synaptic organizing molecules. Simultaneously, our structural analyses

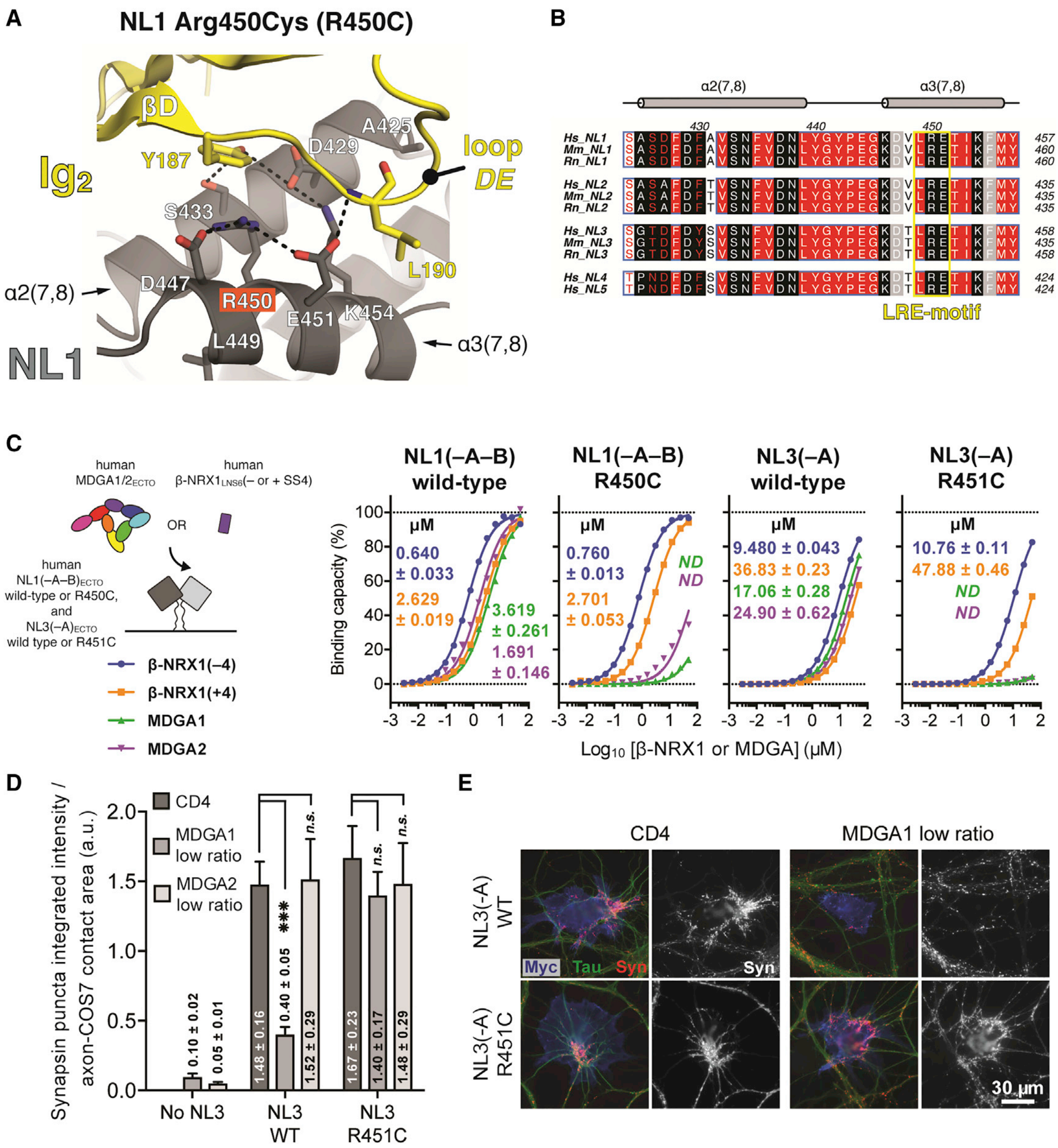

Figure 7. The ASD-Linked NL3 Mutation Arg451Cys Prevents Suppression of Synapse Formation by MDGA1

(A) NL1 Arg450, equivalent to NL3 Arg451, is engaged in π-stacking interactions with Tyr_{MDGA1}187 and its side chain is oriented by charged interactions with Asp_{NL1}447 and Glu_{NL1}451.

(B) Sequence alignment of human, mouse, and rat NL1–5. Helices α2(7,8) and α3(7,8) of *Hs*_NL1 are annotated above the alignment. NL residues unique to the "core" and "rim" of the NL-MDGA interface are highlighted in black and gray, respectively. The Leu-Arg-Glu (LRE) motif, conserved in all NLs and located in the α3(7,8) helix, is boxed in yellow. The equivalent NL1 Arg450 and NL3 Arg451 residues are part of the Site II interface and central to the LRE motif. *Hs*; *Homo sapiens*, *Mm*; *Mus musculus*, *Rn*; *Rattus norvegicus*.

(C) Schematic representation of the SPR setup, summary of K_D values, and binding isotherms for the interaction of NL1, NL1 Arg450Cys, NL3, and NL3 Arg451Cys with $MDGA1\text{-}2_{ECTO}$ and $\beta\text{-}NRX1_{LNS6}(\pm 4)$.

(D) COS-7 cells expressing myc-NL3 wild-type or myc-NL3 Arg451Cys were co-transfected with HA-CD4 control, HA-MDGA1, or HA-MDGA2 and co-cultured with hippocampal neurons. The ability of the co-transfected cells to induce synapsin clustering was measured and normalized to the area of tau-positive axon contact. The bar graphs represent the mean of three independent experiments for the low plasmid ratio (Figure S6A) of human HA-MDGA1-2:myc-NL3 (n > 21 total cells for each condition). Significance is shown for CD4 control versus MDGAs (one-way ANOVA with Bonferroni post hoc comparison). Error bars represent the SEM. ***$p < 0.001$; n.s., not significant. A detailed statistical quantification can be found in Table S5.

(E) Representative images of co-cultures immunostained for surface myc-NL3 (blue), surface HA-MDGA or CD4 control (data not shown), synapsin (red), and tau axonal marker (green). The isolated synapsin signal (white) is shown next to each color image. Scale bar, 30 μm.

See also Figure S8.

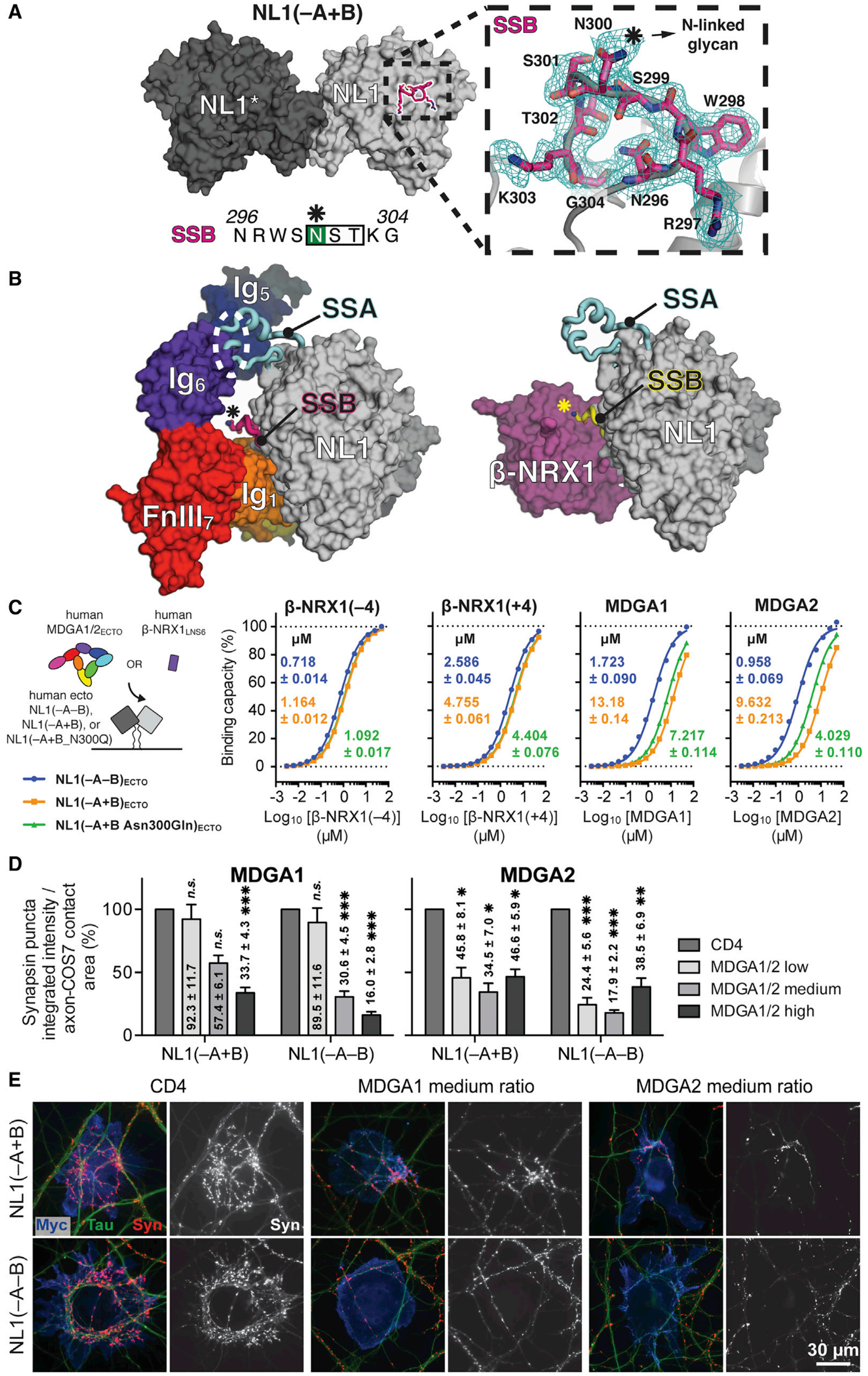

(legend on next page)

guided the discovery of a broad splicing-modulated interaction network between all MDGA and NL isoforms that is able to block NL-NRX complex formation and modulate NL-induced recruitment of synaptic terminals.

Two large, triangular MDGA1 molecules cradle dimeric NL to shield it from interacting with NRX. We tested whether this arrangement also has the potential to negatively influence the interaction of NL with the astrocyte-secreted proteins TSP1 (Xu et al., 2010) and hevin (Singh et al., 2016) and with the NMDAR (Budreck et al., 2013). However, we failed to reproduce these interactions using SPR. Our results suggest that, at least using isolated recombinantly produced proteins and in an SPR setup with defined components and buffer conditions, these interactions are very weak, require the membrane environment, or are mediated through as-yet-unidentified auxiliary proteins or small-molecule ligands. Future studies will have to identify the exact molecular components required for these interactions.

The structure of the NL1-MDGA1 complex uncovers Site II, a hitherto unrecognized interaction site on NL that is distinct from the canonical NL-NRX Site I interface, highlighting the ability of the NL cholinesterase fold to accommodate a diverse array of ligand interaction modes. Furthermore, the NL ΔSite II mutant is a useful molecular tool to selectively uncouple NL-NRX complex formation from inhibition by MDGA, or from other proteins that would utilize Site II.

MDGA Ig domains 1–3 mediate all contacts with NL (Figure 3A). We speculate that MDGA might have more binding partners besides NL. Indeed, the MDGA1 MAM domain binds a receptor on axons (Fujimura et al., 2006) and enhances cell motility and adhesion to non-MDGA1-expressing cells (Díaz-López et al., 2010). The Ig domains 4–6 are reported to play a role in determining synaptic localization of MDGA1 and MDGA2 (Loh et al., 2016). Adhesive interactions of MDGA with as-yet-unidentified partners may be responsible for the MDGA-dependent aggregation of basal progenitor cells in the subventricular zone (Perez-Garcia and O'Leary, 2016), radial migration of cortical neurons (Takeuchi and O'Leary, 2006), and directed axon outgrowth (Ingold et al., 2015; Joset et al., 2011). The widespread expression of NLs (Varoqueaux et al., 2006) and NRXs (Brown et al., 2011; Górecki et al., 1999) from early postnatal ages also raises the interesting possibility that MDGAs may function to shield NLs at the stage of process outgrowth to prevent premature axon-dendrite adhesion and synaptogenesis.

Given the similar interaction affinities of MDGA1-2 and NRX with NL, the balance between NL-NRX and NL-MDGA complex formation will be determined by their relative abundances and binding availability at each synapse in vivo. The net effect of MDGA on synaptic NL-NRX signaling may be influenced by the presence of other protein partners of MDGA, NRX (LRRTMs, calsyntenin 3, dystroglycan, latrophilin 1, cerebellins, and C1q-like proteins), and NL (hevin, thrombospondin, and NMDARs). The complexity of NL-NRX signaling is compounded even further by the existence of postsynaptic *cis* NL-NRX silencing complexes (Taniguchi et al., 2007) and by the recent report of MDGA-like functions for γ-protocadherins (Molumby et al., 2017).

The capacity of NLs to form heterodimers (Poulopoulos et al., 2012) will differentially affect MDGA and NRX binding since the MDGA interface spans both NL monomers, whereas the NRX interface does not. For example, NL1/3, the most prevalent NL heterodimer located at excitatory synapses (Budreck and Scheiffele, 2007; Poulopoulos et al., 2012), would harbor an asymmetric set of Site I-II interfaces: Site II on one side of the dimer will come from NL3, while Site I will be donated by NL1. At the other side of the dimer, this will be inverted. Since NL3 interacts $\sim$10-fold more weakly with MDGA than NL1(–B) (Figure 5A), a composite interface will likely lead to an intermediate strength binding event. Insertion of SSB into NL1 near Site I, however, brings the affinity of NL1 for MDGA in the range of that of NL3 (Figure 8C).

The direct interaction affinities with NL1 and NL2 do not seem to account for selectivity of MDGAs to suppress excitatory or inhibitory synapses. Consistent with the role of MDGA2 to suppress excitatory synapses in vivo (Connor et al., 2016), MDGA2, but not MDGA1, suppressed the synaptogenic activity of the major NL at excitatory synapses, NL1(+B), in co-culture experiments at low-medium ratios (Figure 8D). Yet MDGA2 showed $\sim$12-fold and MDGA1 $\sim$6-fold greater affinity for the major NL at inhibitory synapses, NL2, than for NL1(+B) (Figures 5A and 8C). Factors other than direct MDGA-NL1-2 binding affinities that may contribute include differential glycosylation, although we could find no indication for such (Figures S4C and S4D); additional interacting proteins; or differential cell-type

Figure 8. NL1 SSB Differentially Modulates NL1-NRX and NL1-MDGA Complex Formation

(A) Crystal structure of human NL1(+B). The inset shows 2*m*Fo-*D*Fc electron density contoured at 1.0σ (cyan mesh) for spliced sequence B (SSB). The star symbol indicates the position of the N-linked glycan at Asn300. The glycan tree itself was not visible in the electron density due to structural flexibility.

(B) Structural mapping of spliced sequences A (SSA) and B (SSB) onto NL1. The NL1-MDGA1 and NL1-β-NRX1 interfaces are oriented similarly, based on structural alignment of one NL1 monomer (0.292 Å RMSD over 453 NL1 Cα positions). The position of SSB is derived from the crystal structure of NL1(+B), and the position of SSA is derived from a published crystal structure of rat NL1(+A1) (PDB: 3VKF; Tanaka et al., 2012).

(C) Schematic representation of the SPR setup, summary of K_D values, and binding isotherms for the interaction of human NL1(–A–B)$_{ECTO}$, NL1(–A+B)$_{ECTO}$, and NL1(–A+B_N300Q)$_{ECTO}$ with MDGA1-2$_{ECTO}$ and β-NRX1$_{LNS6}$(±4).

(D) COS-7 cells expressing myc-NLs were co-transfected with HA-CD4 control, HA-MDGA1, or HA-MDGA2 and co-cultured with hippocampal neurons. The ability of the co-transfected cells to induce synapsin clustering was measured and normalized to the area of tau-positive axon contact. The bar graphs represent the mean of three independent experiments for low, medium, and high plasmid ratios (Figure S6A) of human HA-MDGA1-2:myc-NL1(–A ± B) (n > 24 total cells for each ratio) with the CD4:myc-NL1(–A ± B) co-transfected controls normalized to 100% to show the relative change of synapsin integrated intensity at each ratio. Significance is shown for CD4 control versus MDGAs for each NL1 (one-way ANOVA with Bonferroni post hoc comparison). Error bars represent the SEM. *p < 0.05, **p < 0.01, ***p < 0.001; n.s., not significant. A detailed statistical quantification can be found in Table S3.

(E) Representative images of co-cultures immunostained for surface myc-NL (blue), surface HA-MDGA or CD4 control (data not shown), synapsin (red), and tau axonal marker (green). The isolated synapsin signal (white) is shown next to each color image. Scale bar, 30 μm.

See also Figures S9–S11.

expression and subcellular targeting in the brain. As summarized in the introduction, there are conflicting reports on the roles of MDGAs at excitatory versus inhibitory synapses, perhaps related to the use of different model systems, reinforcing the need to consider the native abundance of each molecular player. The newly discovered interaction of MDGAs with NL3 and NL4, particularly the strong association of MDGA1 with NL3 in the pull-down assay and functional modulation of NL3 by MDGA1 in co-culture, may help in better understanding the roles of MDGAs in specific circuits in vivo.

In the rat and mouse brain, MDGA1 and MDGA2 are widely expressed by neuronal populations in both the central and peripheral nervous systems. These include neurons of the basilar pons, inferior olivary nucleus, cerebellum, cerebral cortex, olfactory bulb, spinal cord, dorsal root and trigeminal ganglia, and hippocampus (Connor et al., 2016; Lee et al., 2013; Litwack et al., 2004; Takeuchi et al., 2007). There are regional differences: for example, MDGA1 is more abundant in superficial cortical layers and MDGA2 in deep layers. NL and NRX are also very widely expressed in the mouse brain, such that most neurons likely express NL1–4 and NRX1–3 at varying levels (Hoon et al., 2011; Ullrich et al., 1995; Varoqueaux et al., 2006). We propose that the structural mechanism we described here will be representative for the full range of CNS synapses at which NL, NRX, and MDGA family members are present. Through NL2 and NL4, the range of synapses modulated by MDGA is likely to include glycinergic synapses, not just GABAergic and glutamatergic synapses as shown previously. The differential affinities of specific MDGA and NL isoforms as well as isoform selective interactions of NL with NRX, interactions with other partners regulating bioavailability, and cell-type expression patterns of all molecular players will serve to fine-tune MDGA modulation of synapse development and function.

An important finding of this study is the discovery that both MDGAs interact with and regulate NL3 and NL4. This is of particular interest since rare mutations in NLs, particularly NL3 and NL4, have been associated with ASD and schizophrenia in human genetic studies (Südhof, 2008; Simons Foundation Autism Research Initiative database, https://gene.sfari.org). Interestingly, two mutations in the MDGA interaction-selective Site II of NL3 have been reported in patients with ASD: Arg451Cys (R451C; corresponding to NL1 residue Arg450 and part of the Leu449-Arg450-Glu451 LRE motif) and Gly426Ser (G426S; corresponding to NL1 residue Ala425) (Jamain et al., 2003; Xu et al., 2014) (Figures 7A and 7B). This raises the possibility that selective modulation of MDGA binding to NLs in patients carrying mutations in Site II could contribute to the development of ASD. R451C was characterized as an NL3 gain-of-function mutation in mice, leading to both increased inhibitory synaptic transmission in the somatosensory cortex (Tabuchi et al., 2007) and increased excitatory synaptic transmission in the hippocampus (Etherton et al., 2011), despite resulting in trafficking defects and protein destabilization (Chih et al., 2004; Comoletti et al., 2004). Nonetheless, we observed surface expression of the mutant in both transfected COS-7 cells (Figure S8C) and rat hippocampal neurons (Figure S8D). The latter observation agrees with a report showing cell-surface expression of NL3 R451C in a subset of transfected hippocampal neurons with high expression level (Chih et al., 2004). This also led to an increase in the number of contacting presynaptic terminals, suggesting that the NL3 R451C that trafficked to the surface is functional (Chih et al., 2004). Importantly, we found that, similarly to the NL ΔSite II mutant, the NL3 R451C mutation selectively uncoupled NL3-NRX binding and recruitment of synaptic terminals from inhibition by MDGA1 (Figure 7D), suggesting that the R451C gain-of-function phenotype is achieved by preventing the inhibition of NL3 by MDGA1, thereby leading to disruption of the overall balance of excitatory/inhibitory (E/I) synaptic transmission.

An E/I imbalance surpassing the capacity of neuronal populations and circuits to regulate synaptic homeostasis is a proposed hallmark of ASD (Nelson and Valakh, 2015; Rubenstein and Merzenich, 2003). Disruptions in the regulatory NL-MDGA network we report here contribute to ASD based on human genetics (Bucan et al., 2009; Kähler et al., 2008; Li et al., 2011; Südhof, 2008) and can generate such an E/I imbalance in animal models (e.g., Connor et al., 2016; Tabuchi et al., 2007). Our findings considerably broadened this interaction network beyond that previously envisioned. Moreover, our structural studies constitute an essential guide toward the generation of directed therapies targeting these gene products to restore E/I balance.

STAR★METHODS

Detailed methods are provided in the online version of this paper and include the following:

- KEY RESOURCES TABLE
- CONTACT FOR REAGENT AND RESOURCE SHARING
- METHOD DETAILS
  - Expression and purification of recombinant proteins
  - Expression and purification of recombinant NMDA receptor
  - Gene splicing and site-directed mutagenesis
  - Protein crystallization
  - Crystallographic data collection and structure determination
  - Crystallographic refinement and model analysis
  - Sequence alignments and conservation analysis
  - Small-angle X-ray scattering (SAXS)
  - Surface plasmon resonance (SPR) with soluble proteins
  - Surface plasmon resonance (SPR) with the NMDA receptor
  - Isothermal titration calorimetry (ITC)
  - Analytical ultracentrifugation (AUC)
  - Co-culture and immunocytochemistry
  - Image acquisition and analysis
  - Surface expression of the NL3(–A) Arg451Cys mutant
  - Pulldown and mass spectrometry
- DATA AND SOFTWARE AVAILABILITY

SUPPLEMENTAL INFORMATION

Supplemental Information includes eleven figures and five tables and can be found with this article online at http://dx.doi.org/10.1016/j.neuron.2017.07.040.

AUTHOR CONTRIBUTIONS

All authors contributed to experimental design. J.E., A.J.C., C.H., W.J., and A.C.S. produced constructs and recombinant proteins. A.J.C. and J.E. performed crystallization experiments. J.E., A.J.C., and A.R.A. collected X-ray data and solved the crystal structures. J.E. performed the biophysical experiments. V.C. performed the co-culture and immunocytochemistry experiments. K.M.V., S.N.S., J.N.S., and J.d.W. performed mass spectrometry and pull-down experiments. M.C.R. and H.F. produced the NMDAR samples. J.B., A.M.C., and A.R.A. directed research. J.E. wrote the manuscript, with input from V.C., A.M.C., and A.R.A. All authors provided feedback on the final manuscript version.

ACKNOWLEDGMENTS

We thank the staff at Diamond Light Source (DLS; beamlines I03, I04-1, and I24), and the European Synchrotron Radiation Facility (ESRF; beamline BM29) for support. We thank T. Walter and K. Harlos for crystallization technical support, E. Behiels for assistance with SPR data analysis, and X. Zhou for assistance with neuron cultures. This work was funded by the UK Medical Research Council (MRC; G0700232, L009609, and MC_UP_1201/15 to A.R.A.), Canadian Institutes of Health Research (CIHR; FDN-143206 to A.M.C.), and ERA-NET NEURON 2015 Cofund Program under Horizon 2020 (to J.d.W and via CIHR NDD-144222 to A.M.C.). J.N.S. is supported by National Institutes of Health (NIH) grant R00 DC013805. J.E. was supported by European Molecular Biology Organization (EMBO; ALTF 1116-2012) and Marie-Curie (FP7-328531) long-term postdoctoral fellowships. V.C. was supported by CIHR (338646) and Michael Smith Foundation for Health Research (16005) postdoctoral fellowships. A.J.C. was partly supported by Cancer Research UK (CRUK; grant C375/A10976 to E.Y. Jones). C.H. is a Nuffield Department of Medicine (NDM) Prize Student. H.F. and M.C.R. were supported by the NIH (MH085926 and GM105730 to H.F. and F32NS093753 to M.C.R.). A.M.C. is a Canada Research Chair. A.R.A. is an MRC senior research fellow. The Wellcome Trust Centre for Human Genetics is supported by Wellcome Trust grant 090532/Z/09/Z.

Received: November 19, 2016
Revised: June 22, 2017
Accepted: July 28, 2017
Published: August 16, 2017

REFERENCES

Adams, P.D., Afonine, P.V., Bunkóczi, G., Chen, V.B., Davis, I.W., Echols, N., Headd, J.J., Hung, L.W., Kapral, G.J., Grosse-Kunstleve, R.W., et al. (2010). PHENIX: a comprehensive Python-based system for macromolecular structure solution. Acta Crystallogr. D Biol. Crystallogr. *66*, 213–221.

Altschul, S.F., Gish, W., Miller, W., Myers, E.W., and Lipman, D.J. (1990). Basic local alignment search tool. J. Mol. Biol. *215*, 403–410.

Aoto, J., Martinelli, D.C., Malenka, R.C., Tabuchi, K., and Südhof, T.C. (2013). Presynaptic neurexin-3 alternative splicing trans-synaptically controls postsynaptic AMPA receptor trafficking. Cell *154*, 75–88.

Araç, D., Boucard, A.A., Ozkan, E., Strop, P., Newell, E., Südhof, T.C., and Brunger, A.T. (2007). Structures of neuroligin-1 and the neuroligin-1/neurexin-1 beta complex reveal specific protein-protein and protein-Ca2+ interactions. Neuron *56*, 992–1003.

Aricescu, A.R., Hon, W.C., Siebold, C., Lu, W., van der Merwe, P.A., and Jones, E.Y. (2006a). Molecular analysis of receptor protein tyrosine phosphatase mu-mediated cell adhesion. EMBO J. *25*, 701–712.

Aricescu, A.R., Lu, W., and Jones, E.Y. (2006b). A time- and cost-efficient system for high-level protein production in mammalian cells. Acta Crystallogr. D Biol. Crystallogr. *62*, 1243–1250.

Ashkenazy, H., Erez, E., Martz, E., Pupko, T., and Ben-Tal, N. (2010). ConSurf 2010: calculating evolutionary conservation in sequence and structure of proteins and nucleic acids. Nucleic Acids Res. *38*, W529-33.

Bemben, M.A., Shipman, S.L., Nicoll, R.A., and Roche, K.W. (2015). The cellular and molecular landscape of neuroligins. Trends Neurosci. *38*, 496–505.

Biasini, M., Bienert, S., Waterhouse, A., Arnold, K., Studer, G., Schmidt, T., Kiefer, F., Gallo Cassarino, T., Bertoni, M., Bordoli, L., and Schwede, T. (2014). SWISS-MODEL: modelling protein tertiary and quaternary structure using evolutionary information. Nucleic Acids Res. *42*, W252-8.

Bond, C.S., and Schüttelkopf, A.W. (2009). ALINE: a WYSIWYG protein-sequence alignment editor for publication-quality alignments. Acta Crystallogr. D Biol. Crystallogr. *65*, 510–512.

Boucard, A.A., Chubykin, A.A., Comoletti, D., Taylor, P., and Südhof, T.C. (2005). A splice code for trans-synaptic cell adhesion mediated by binding of neuroligin 1 to alpha- and beta-neurexins. Neuron *48*, 229–236.

Bourne, Y., Taylor, P., and Marchot, P. (1995). Acetylcholinesterase inhibition by fasciculin: crystal structure of the complex. Cell *83*, 503–512.

Brautigam, C.A. (2015). Calculations and publication-quality illustrations for analytical ultracentrifugation data. Methods Enzymol. *562*, 109–133.

Brown, P.H., and Schuck, P. (2006). Macromolecular size-and-shape distributions by sedimentation velocity analytical ultracentrifugation. Biophys. J. *90*, 4651–4661.

Brown, S.M., Clapcote, S.J., Millar, J.K., Torrance, H.S., Anderson, S.M., Walker, R., Rampino, A., Roder, J.C., Thomson, P.A., Porteous, D.J., and Evans, K.L. (2011). Synaptic modulators Nrxn1 and Nrxn3 are disregulated in a Disc1 mouse model of schizophrenia. Mol. Psychiatry *16*, 585–587.

Bucan, M., Abrahams, B.S., Wang, K., Glessner, J.T., Herman, E.I., Sonnenblick, L.I., Alvarez Retuerto, A.I., Imielinski, M., Hadley, D., Bradfield, J.P., et al. (2009). Genome-wide analyses of exonic copy number variants in a family-based study point to novel autism susceptibility genes. PLoS Genet. *5*, e1000536.

Budreck, E.C., and Scheiffele, P. (2007). Neuroligin-3 is a neuronal adhesion protein at GABAergic and glutamatergic synapses. Eur. J. Neurosci. *26*, 1738–1748.

Budreck, E.C., Kwon, O.B., Jung, J.H., Baudouin, S., Thommen, A., Kim, H.S., Fukazawa, Y., Harada, H., Tabuchi, K., Shigemoto, R., et al. (2013). Neuroligin-1 controls synaptic abundance of NMDA-type glutamate receptors through extracellular coupling. Proc. Natl. Acad. Sci. USA *110*, 725–730.

Chen, X., Liu, H., Shim, A.H., Focia, P.J., and He, X. (2008). Structural basis for synaptic adhesion mediated by neuroligin-neurexin interactions. Nat. Struct. Mol. Biol. *15*, 50–56.

Chen, V.B., Arendall, W.B., 3rd, Headd, J.J., Keedy, D.A., Immormino, R.M., Kapral, G.J., Murray, L.W., Richardson, J.S., and Richardson, D.C. (2010). MolProbity: all-atom structure validation for macromolecular crystallography. Acta Crystallogr. D Biol. Crystallogr. *66*, 12–21.

Chih, B., Afridi, S.K., Clark, L., and Scheiffele, P. (2004). Disorder-associated mutations lead to functional inactivation of neuroligins. Hum. Mol. Genet. *13*, 1471–1477.

Chih, B., Gollan, L., and Scheiffele, P. (2006). Alternative splicing controls selective trans-synaptic interactions of the neuroligin-neurexin complex. Neuron *51*, 171–178.

Comoletti, D., Flynn, R., Jennings, L.L., Chubykin, A., Matsumura, T., Hasegawa, H., Südhof, T.C., and Taylor, P. (2003). Characterization of the interaction of a recombinant soluble neuroligin-1 with neurexin-1beta. J. Biol. Chem. *278*, 50497–50505.

Comoletti, D., De Jaco, A., Jennings, L.L., Flynn, R.E., Gaietta, G., Tsigelny, I., Ellisman, M.H., and Taylor, P. (2004). The Arg451Cys-neuroligin-3 mutation associated with autism reveals a defect in protein processing. J. Neurosci. *24*, 4889–4893.

Connor, S.A., Ammendrup-Johnsen, I., Chan, A.W., Kishimoto, Y., Murayama, C., Kurihara, N., Tada, A., Ge, Y., Lu, H., Yan, R., et al. (2016). Altered cortical dynamics and cognitive function upon haploinsufficiency of the autism-linked excitatory synaptic suppressor MDGA2. Neuron *91*, 1052–1068.

de Wit, J., and Ghosh, A. (2016). Specification of synaptic connectivity by cell surface interactions. Nat. Rev. Neurosci. *17*, 22–35.

Díaz-López, A., Iniesta, P., Morán, A., Ortega, P., Fernández-Marcelo, T., Sánchez-Pernaute, A., Torres, A.J., Benito, M., and De Juan, C. (2010). Expression of human MDGA1 increases cell motility and cell-cell adhesion and reduces adhesion to extracellular matrix proteins in MDCK cells. Cancer Microenviron. *4*, 23–32.

Edgar, R.C. (2004). MUSCLE: multiple sequence alignment with high accuracy and high throughput. Nucleic Acids Res. *32*, 1792–1797.

Elegheert, J., Kakegawa, W., Clay, J.E., Shanks, N.F., Behiels, E., Matsuda, K., Kohda, K., Miura, E., Rossmann, M., Mitakidis, N., et al. (2016). Structural basis for integration of GluD receptors within synaptic organizer complexes. Science *353*, 295–299.

Emsley, P., Lohkamp, B., Scott, W.G., and Cowtan, K. (2010). Features and development of Coot. Acta Crystallogr. D Biol. Crystallogr. *66*, 486–501.

Etherton, M., Földy, C., Sharma, M., Tabuchi, K., Liu, X., Shamloo, M., Malenka, R.C., and Südhof, T.C. (2011). Autism-linked neuroligin-3 R451C mutation differentially alters hippocampal and cortical synaptic function. Proc. Natl. Acad. Sci. USA *108*, 13764–13769.

Evans, P. (2006). Scaling and assessment of data quality. Acta Crystallogr. D Biol. Crystallogr. *62*, 72–82.

Evans, P.R. (2011). An introduction to data reduction: space-group determination, scaling and intensity statistics. Acta Crystallogr. D Biol. Crystallogr. *67*, 282–292.

Fabrichny, I.P., Leone, P., Sulzenbacher, G., Comoletti, D., Miller, M.T., Taylor, P., Bourne, Y., and Marchot, P. (2007). Structural analysis of the synaptic protein neuroligin and its beta-neurexin complex: determinants for folding and cell adhesion. Neuron *56*, 979–991.

Fujimura, Y., Iwashita, M., Matsuzaki, F., and Yamamoto, T. (2006). MDGA1, an IgSF molecule containing a MAM domain, heterophilically associates with axon- and muscle-associated binding partners through distinct structural domains. Brain Res. *1101*, 12–19.

Górecki, D.C., Szklarczyk, A., Lukasiuk, K., Kaczmarek, L., and Simons, J.P. (1999). Differential seizure-induced and developmental changes of neurexin expression. Mol. Cell. Neurosci. *13*, 218–227.

Graf, E.R., Zhang, X., Jin, S.X., Linhoff, M.W., and Craig, A.M. (2004). Neurexins induce differentiation of GABA and glutamate postsynaptic specializations via neuroligins. Cell *119*, 1013–1026.

Guttman, M., Weinkam, P., Sali, A., and Lee, K.K. (2013). All-atom ensemble modeling to analyze small-angle x-ray scattering of glycosylated proteins. Structure *21*, 321–331.

Hammel, M. (2012). Validation of macromolecular flexibility in solution by small-angle X-ray scattering (SAXS). Eur. Biophys. J. *41*, 789–799.

Harel, M., Kleywegt, G.J., Ravelli, R.B., Silman, I., and Sussman, J.L. (1995). Crystal structure of an acetylcholinesterase-fasciculin complex: interaction of a three-fingered toxin from snake venom with its target. Structure *3*, 1355–1366.

Heckman, K.L., and Pease, L.R. (2007). Gene splicing and mutagenesis by PCR-driven overlap extension. Nat. Protoc. *2*, 924–932.

Hoon, M., Soykan, T., Falkenburger, B., Hammer, M., Patrizi, A., Schmidt, K.F., Sassoè-Pognetto, M., Löwel, S., Moser, T., Taschenberger, H., et al. (2011). Neuroligin-4 is localized to glycinergic postsynapses and regulates inhibition in the retina. Proc. Natl. Acad. Sci. USA *108*, 3053–3058.

Howarth, M., Liu, W., Puthenveetil, S., Zheng, Y., Marshall, L.F., Schmidt, M.M., Wittrup, K.D., Bawendi, M.G., and Ting, A.Y. (2008). Monovalent, reduced-size quantum dots for imaging receptors on living cells. Nat. Methods *5*, 397–399.

Ingold, E., Vom Berg-Maurer, C.M., Burckhardt, C.J., Lehnherr, A., Rieder, P., Keller, P.J., Stelzer, E.H., Greber, U.F., Neuhauss, S.C., and Gesemann, M. (2015). Proper migration and axon outgrowth of zebrafish cranial motoneuron subpopulations require the cell adhesion molecule MDGA2A. Biol. Open *4*, 146–154.

Jamain, S., Quach, H., Betancur, C., Råstam, M., Colineaux, C., Gillberg, I.C., Soderstrom, H., Giros, B., Leboyer, M., Gillberg, C., and Bourgeron, T.; Paris Autism Research International Sibpair Study (2003). Mutations of the X-linked genes encoding neuroligins NLGN3 and NLGN4 are associated with autism. Nat. Genet. *34*, 27–29.

Johnson, G., and Moore, S.W. (2013). The Leu-Arg-Glu (LRE) adhesion motif in proteins of the neuromuscular junction with special reference to proteins of the carboxylesterase/cholinesterase family. Comp. Biochem. Physiol. Part D Genomics Proteomics *8*, 231–243.

Joset, P., Wacker, A., Babey, R., Ingold, E.A., Andermatt, I., Stoeckli, E.T., and Gesemann, M. (2011). Rostral growth of commissural axons requires the cell adhesion molecule MDGA2. Neural Dev. *6*, 22.

Kabsch, W. (2010). Xds. Acta Crystallogr. D Biol. Crystallogr. *66*, 125–132.

Kaech, S., and Banker, G. (2006). Culturing hippocampal neurons. Nat. Protoc. *1*, 2406–2415.

Kähler, A.K., Djurovic, S., Kulle, B., Jönsson, E.G., Agartz, I., Hall, H., Opjordsmoen, S., Jakobsen, K.D., Hansen, T., Melle, I., et al. (2008). Association analysis of schizophrenia on 18 genes involved in neuronal migration: MDGA1 as a new susceptibility gene. Am. J. Med. Genet. B. Neuropsychiatr. Genet. *147B*, 1089–1100.

Karakas, E., and Furukawa, H. (2014). Crystal structure of a heterotetrameric NMDA receptor ion channel. Science *344*, 992–997.

Koehnke, J., Katsamba, P.S., Ahlsen, G., Bahna, F., Vendome, J., Honig, B., Shapiro, L., and Jin, X. (2010). Splice form dependence of beta-neurexin/neuroligin binding interactions. Neuron *67*, 61–74.

Krissinel, E., and Henrick, K. (2007). Inference of macromolecular assemblies from crystalline state. J. Mol. Biol. *372*, 774–797.

Kvansakul, M., Adams, J.C., and Hohenester, E. (2004). Structure of a thrombospondin C-terminal fragment reveals a novel calcium core in the type 3 repeats. EMBO J. *23*, 1223–1233.

Lee, K., Kim, Y., Lee, S.J., Qiang, Y., Lee, D., Lee, H.W., Kim, H., Je, H.S., Südhof, T.C., and Ko, J. (2013). MDGAs interact selectively with neuroligin-2 but not other neuroligins to regulate inhibitory synapse development. Proc. Natl. Acad. Sci. USA *110*, 336–341.

Li, J., Liu, J., Feng, G., Li, T., Zhao, Q., Li, Y., Hu, Z., Zheng, L., Zeng, Z., He, L., et al. (2011). The MDGA1 gene confers risk to schizophrenia and bipolar disorder. Schizophr. Res. *125*, 194–200.

Litwack, E.D., Babey, R., Buser, R., Gesemann, M., and O'Leary, D.D. (2004). Identification and characterization of two novel brain-derived immunoglobulin superfamily members with a unique structural organization. Mol. Cell. Neurosci. *25*, 263–274.

Loh, K.H., Stawski, P.S., Draycott, A.S., Udeshi, N.D., Lehrman, E.K., Wilton, D.K., Svinkina, T., Deerinck, T.J., Ellisman, M.H., Stevens, B., et al. (2016). Proteomic analysis of unbounded cellular compartments: synaptic clefts. Cell *166*, 1295–1307.e21.

Loriot, S., and Cazals, F. (2010). Modeling macro-molecular interfaces with Intervor. Bioinformatics *26*, 964–965.

Matsuda, K., Budisantoso, T., Mitakidis, N., Sugaya, Y., Miura, E., Kakegawa, W., Yamasaki, M., Konno, K., Uchigashima, M., Abe, M., et al. (2016). Transsynaptic modulation of kainate receptor functions by C1q-like proteins. Neuron *90*, 752–767.

McCoy, A.J., Grosse-Kunstleve, R.W., Adams, P.D., Winn, M.D., Storoni, L.C., and Read, R.J. (2007). Phaser crystallographic software. J. Appl. Cryst. *40*, 658–674.

Molumby, M.J., Anderson, R.M., Newbold, D.J., Koblesky, N.K., Garrett, A.M., Schreiner, D., Radley, J.J., and Weiner, J.A. (2017). γ-Protocadherins interact with neuroligin-1 and negatively regulate dendritic spine morphogenesis. Cell Rep. *18*, 2702–2714.

Murshudov, G.N., Skubák, P., Lebedev, A.A., Pannu, N.S., Steiner, R.A., Nicholls, R.A., Winn, M.D., Long, F., and Vagin, A.A. (2011). REFMAC5 for the refinement of macromolecular crystal structures. Acta Crystallogr. D Biol. Crystallogr. *67*, 355–367.

Myszka, D.G. (1999). Improving biosensor analysis. J. Mol. Recognit. *12*, 279–284.

Nelson, S.B., and Valakh, V. (2015). Excitatory/inhibitory balance and circuit homeostasis in autism spectrum disorders. Neuron *87*, 684–698.

Perez-Garcia, C.G., and O'Leary, D.D. (2016). Formation of the cortical subventricular zone requires MDGA1-mediated aggregation of basal progenitors. Cell Rep. *14*, 560–571.

Pernot, P., Round, A., Barrett, R., De Maria Antolinos, A., Gobbo, A., Gordon, E., Huet, J., Kieffer, J., Lentini, M., Mattenet, M., et al. (2013). Upgraded ESRF BM29 beamline for SAXS on macromolecules in solution. J. Synchrotron Radiat. *20*, 660–664.

Petoukhov, M.V., Franke, D., Shkumatov, A.V., Tria, G., Kikhney, A.G., Gajda, M., Gorba, C., Mertens, H.D.T., Konarev, P.V., and Svergun, D.I. (2012). New developments in the ATSAS program package for small-angle scattering data analysis. J. Appl. Cryst. *45*, 342–350.

Pettem, K.L., Yokomaku, D., Takahashi, H., Ge, Y., and Craig, A.M. (2013). Interaction between autism-linked MDGAs and neuroligins suppresses inhibitory synapse development. J. Cell Biol. *200*, 321–336.

Pettersen, E.F., Goddard, T.D., Huang, C.C., Couch, G.S., Greenblatt, D.M., Meng, E.C., and Ferrin, T.E. (2004). UCSF Chimera—a visualization system for exploratory research and analysis. J. Comput. Chem. *25*, 1605–1612.

Poulopoulos, A., Soykan, T., Tuffy, L.P., Hammer, M., Varoqueaux, F., and Brose, N. (2012). Homodimerization and isoform-specific heterodimerization of neuroligins. Biochem. J. *446*, 321–330.

Rambo, R.P., and Tainer, J.A. (2013). Accurate assessment of mass, models and resolution by small-angle scattering. Nature *496*, 477–481.

Reeves, P.J., Callewaert, N., Contreras, R., and Khorana, H.G. (2002). Structure and function in rhodopsin: high-level expression of rhodopsin with restricted and homogeneous N-glycosylation by a tetracycline-inducible N-acetylglucosaminyltransferase I-negative HEK293S stable mammalian cell line. Proc. Natl. Acad. Sci. USA *99*, 13419–13424.

Reissner, C., Runkel, F., and Missler, M. (2013). Neurexins. Genome Biol. *14*, 213.

Rubenstein, J.L., and Merzenich, M.M. (2003). Model of autism: increased ratio of excitation/inhibition in key neural systems. Genes Brain Behav. *2*, 255–267.

Sauter, N.K., Grosse-Kunstleve, R.W., and Adams, P.D. (2004). Robust indexing for automatic data collection. J. Appl. Cryst. *37*, 399–409.

Savas, J.N., De Wit, J., Comoletti, D., Zemla, R., Ghosh, A., and Yates, J.R., 3rd (2014). Ecto-Fc MS identifies ligand-receptor interactions through extracellular domain Fc fusion protein baits and shotgun proteomic analysis. Nat. Protoc. *9*, 2061–2074.

Schneider, T.R., and Sheldrick, G.M. (2002). Substructure solution with SHELXD. Acta Crystallogr. D Biol. Crystallogr. *58*, 1772–1779.

Schneider, C.A., Rasband, W.S., and Eliceiri, K.W. (2012). NIH Image to ImageJ: 25 years of image analysis. Nat. Methods *9*, 671–675.

Schneidman-Duhovny, D., Hammel, M., Tainer, J.A., and Sali, A. (2013). Accurate SAXS profile computation and its assessment by contrast variation experiments. Biophys. J. *105*, 962–974.

Schreiner, D., Nguyen, T.M., Russo, G., Heber, S., Patrignani, A., Ahrné, E., and Scheiffele, P. (2014). Targeted combinatorial alternative splicing generates brain region-specific repertoires of neurexins. Neuron *84*, 386–398.

Schrodinger, L.L.C. (2010). The PyMOL Molecular Graphics System. https://www.pymol.org/.

Siddiqui, T.J., and Craig, A.M. (2011). Synaptic organizing complexes. Curr. Opin. Neurobiol. *21*, 132–143.

Siddiqui, T.J., Pancaroglu, R., Kang, Y., Rooyakkers, A., and Craig, A.M. (2010). LRRTMs and neuroligins bind neurexins with a differential code to cooperate in glutamate synapse development. J. Neurosci. *30*, 7495–7506.

Singh, S.K., Stogsdill, J.A., Pulimood, N.S., Dingsdale, H., Kim, Y.H., Pilaz, L.J., Kim, I.H., Manhaes, A.C., Rodrigues, W.S., Jr., Pamukcu, A., et al. (2016). Astrocytes assemble thalamocortical synapses by bridging NRX1α and NL1 via hevin. Cell *164*, 183–196.

Song, J.Y., Ichtchenko, K., Südhof, T.C., and Brose, N. (1999). Neuroligin 1 is a postsynaptic cell-adhesion molecule of excitatory synapses. Proc. Natl. Acad. Sci. USA *96*, 1100–1105.

Südhof, T.C. (2008). Neuroligins and neurexins link synaptic function to cognitive disease. Nature *455*, 903–911.

Tabuchi, K., Blundell, J., Etherton, M.R., Hammer, R.E., Liu, X., Powell, C.M., and Südhof, T.C. (2007). A neuroligin-3 mutation implicated in autism increases inhibitory synaptic transmission in mice. Science *318*, 71–76.

Takeuchi, A., and O'Leary, D.D. (2006). Radial migration of superficial layer cortical neurons controlled by novel Ig cell adhesion molecule MDGA1. J. Neurosci. *26*, 4460–4464.

Takeuchi, A., Hamasaki, T., Litwack, E.D., and O'Leary, D.D. (2007). Novel IgCAM, MDGA1, expressed in unique cortical area- and layer-specific patterns and transiently by distinct forebrain populations of Cajal-Retzius neurons. Cereb. Cortex *17*, 1531–1541.

Tanaka, H., Miyazaki, N., Matoba, K., Nogi, T., Iwasaki, K., and Takagi, J. (2012). Higher-order architecture of cell adhesion mediated by polymorphic synaptic adhesion molecules neurexin and neuroligin. Cell Rep. *2*, 101–110.

Taniguchi, H., Gollan, L., Scholl, F.G., Mahadomrongkul, V., Dobler, E., Limthong, N., Peck, M., Aoki, C., and Scheiffele, P. (2007). Silencing of neuroligin function by postsynaptic neurexins. J. Neurosci. *27*, 2815–2824.

Terwilliger, T.C., Adams, P.D., Read, R.J., McCoy, A.J., Moriarty, N.W., Grosse-Kunstleve, R.W., Afonine, P.V., Zwart, P.H., and Hung, L.W. (2009). Decision-making in structure solution using Bayesian estimates of map quality: the PHENIX AutoSol wizard. Acta Crystallogr. D Biol. Crystallogr. *65*, 582–601.

Uemura, T., Lee, S.J., Yasumura, M., Takeuchi, T., Yoshida, T., Ra, M., Taguchi, R., Sakimura, K., and Mishina, M. (2010). Trans-synaptic interaction of GluRdelta2 and neurexin through Cbln1 mediates synapse formation in the cerebellum. Cell *141*, 1068–1079.

Ullrich, B., Ushkaryov, Y.A., and Südhof, T.C. (1995). Cartography of neurexins: more than 1000 isoforms generated by alternative splicing and expressed in distinct subsets of neurons. Neuron *14*, 497–507.

Varoqueaux, F., Jamain, S., and Brose, N. (2004). Neuroligin 2 is exclusively localized to inhibitory synapses. Eur. J. Cell Biol. *83*, 449–456.

Varoqueaux, F., Aramuni, G., Rawson, R.L., Mohrmann, R., Missler, M., Gottmann, K., Zhang, W., Südhof, T.C., and Brose, N. (2006). Neuroligins determine synapse maturation and function. Neuron *51*, 741–754.

Walter, T.S., Diprose, J.M., Mayo, C.J., Siebold, C., Pickford, M.G., Carter, L., Sutton, G.C., Berrow, N.S., Brown, J., Berry, I.M., et al. (2005). A procedure for setting up high-throughput nanolitre crystallization experiments. Crystallization workflow for initial screening, automated storage, imaging and optimization. Acta Crystallogr. D Biol. Crystallogr. *61*, 651–657.

Walter, T.S., Mancini, E.J., Kadlec, J., Graham, S.C., Assenberg, R., Ren, J., Sainsbury, S., Owens, R.J., Stuart, D.I., Grimes, J.M., and Harlos, K. (2008). Semi-automated microseeding of nanolitre crystallization experiments. Acta Crystallogr. Sect. F Struct. Biol. Cryst. Commun. *64*, 14–18.

Webb, B., and Sali, A. (2014). Comparative protein structure modeling using MODELLER. Curr. Protoc. Bioinformatics *47*, 1–32.

Weinkam, P., Pons, J., and Sali, A. (2012). Structure-based model of allostery predicts coupling between distant sites. Proc. Natl. Acad. Sci. USA *109*, 4875–4880.

Winter, G., Lobley, C.M., and Prince, S.M. (2013). Decision making in xia2. Acta Crystallogr. D Biol. Crystallogr. *69*, 1260–1273.

Xu, J., Xiao, N., and Xia, J. (2010). Thrombospondin 1 accelerates synaptogenesis in hippocampal neurons through neuroligin 1. Nat. Neurosci. *13*, 22–24.

Xu, X., Xiong, Z., Zhang, L., Liu, Y., Lu, L., Peng, Y., Guo, H., Zhao, J., Xia, K., and Hu, Z. (2014). Variations analysis of NLGN3 and NLGN4X gene in Chinese autism patients. Mol. Biol. Rep. *41*, 4133–4140.

STAR★METHODS

KEY RESOURCES TABLE

REAGENT or RESOURCE	SOURCE	IDENTIFIER
Antibodies		
Anti-c-myc, rabbit polyclonal	Sigma-Aldrich	Cat# C3956; RRID: AB_439680
Anti-HA, mouse monoclonal IgG2b	Roche	Cat# 11583816001; RRID: AB_514505
Anti-synapsin1, mouse monoclonal IgG1	Synaptic Systems	Cat # 106011
Anti-tau, mouse monoclonal IgG2a	Millipore	Cat# MAB3420; RRID: AB_94855
Anti-V5, mouse monoclonal IgG2a	Thermo Fisher	Cat# R960-25; RRID: AB_2556564
AMCA goat anti-rabbit IgG (H+L)	Jackson ImmunoResearch	Cat# 111-155-144; RRID: AB_2337994
Alexa Fluor 488 goat anti-rabbit IgG (H+L)	Thermo Fisher	Cat# R37116; RRID: AB_2556544
Alexa Fluor 488 goat anti-mouse IgG2b	Thermo Fisher	Cat# A-21141; RRID: AB_2535778
Alexa Fluor 568 goat anti-mouse IgG1	Thermo Fisher	Cat# A-21124; RRID: AB_2535766
Alexa Fluor 647 goat anti-mouse IgG2a	Thermo Fisher	Cat# A-21241; RRID: AB_2535810
Streptavidin-HRP conjugate	Sigma-Aldrich	Cat# GERPN1231
Chemicals, Peptides, and Recombinant Proteins		
Neurobasal Medium	Thermo Fisher	Cat# 21103049
GlutaMAX	Thermo Fisher	Cat# 35050061
B27 serum-free supplement	Thermo Fisher	Cat# 17504044
APV	Abcam	Cat# ab120271
Dulbecco's Modified Eagle Medium, high glucose	Sigma-Aldrich	Cat# D5796
Dulbecco's Modified Eagle Medium, high glucose, no L-Methionine	Sigma-Aldrich	Cat# D0422
Bovine growth serum	GE Healthcare	Cat# SH30541.03
Penicillin/streptomycin	Thermo Fisher	Cat# 15070063
TransIT-LT1 transfection reagent	Mirus Bio	Cat# MIR2305
Pyrobest DNA Polymerase	Takara	Cat# R005A
Seleno-L-Methionine	Sigma-Aldrich	Cat# S3132
D-biotin	Sigma-Aldrich	Cat# B4639
Streptavidin	Sigma-Aldrich	Cat# S4762
Bovine serum albumin	Sigma-Aldrich	Cat# A7638
Polyethylenimine, branched	Sigma-Aldrich	Cat# 408727
Ammonium bicarbonate	Sigma-Aldrich	Cat# 09830
Urea	Thermo Scientific	Cat# 29700
Tris(2-carboxyethyl)phosphine hydrochloride (TCEP)	Sigma-Aldrich	Cat# C4706
Iodoacetamide	Sigma-Aldrich	Cat# I1149
Pierce Trypsin Protease, MS Grade	Thermo Scientific	Cat# 90057
ProteaseMAX Surfactant, Trypsin Enhancer	Promega	Cat# V2072
Formic Acid Optima LC/MS	Thermo Fisher	Cat# A117
Trifluoroacetic Acid (TFA)	Thermo Fisher	Cat# O4902
Acetonitrile Optima LC/MS	Thermo Fisher	Cat# A955
Deposited Data		
hNL1(–A+B)$_{ECTO}$	This paper	PDB: 5OJK
cMDGA1$_{ECTO}$	This paper	PDB: 5OJ2
hNL1$_{ECTO}$–cMDGA1$_{ECTO}$	This paper	PDB: 5OJ6

(*Continued on next page*)

Continued

REAGENT or RESOURCE	SOURCE	IDENTIFIER
Experimental Models: Cell Lines		
COS-7	ATCC	Cat# CRL-1651; RRID: CVCL_0224
HEK293T	ATCC	Cat# CRL-3216; RRID: CVCL_0063
HEK293S GnTI$^{-/-}$	ATCC	Cat# CRL-3022; RRID: CVCL_A785
Experimental Models: Organisms/Strains		
Sprague Dawley rat, female timed pregnant d18	Charles River Canada	Strain code 400
Software and Algorithms		
MetaMorph	Molecular Devices	https://www.moleculardevices.com/systems/metamorph-research-imaging/metamorph-microscopy-automation-and-image-analysis-software
ImageJ	Schneider et al., 2012	https://imagej.nih.gov/ij/download.html
GraphPad Prism	GraphPad Software	http://www.graphpad.com/scientific-software/prism/
SHELXD	Schneider and Sheldrick, 2002	http://shelx.uni-ac.gwdg.de/SHELX/
Phenix	Adams et al., 2010	https://www.phenix-online.org/
XIA2	Winter et al., 2013	http://xds.mpimf-heidelberg.mpg.de/
PISA	Krissinel and Henrick, 2007	http://www.ebi.ac.uk/pdbe/pisa/
Intervor	Loriot and Cazals, 2010	N/A
Coot	Emsley et al., 2010	http://www2.mrc-lmb.cam.ac.uk/personal/pemsley/coot/
PyMOL	Schrodinger, 2010	https://www.pymol.org/
BLAST	Altschul et al., 1990	https://blast.ncbi.nlm.nih.gov/
MUSCLE	Edgar, 2004	http://www.ebi.ac.uk/Tools/msa/muscle/
ALINE	Bond and Schüttelkopf, 2009	http://bondxray.org/software/aline.html
Consurf	Ashkenazy et al., 2010	http://consurf.tau.ac.il/2016/
ATSAS	Petoukhov et al., 2012	https://www.embl-hamburg.de/biosaxs/software.html
ScÅtter	Rambo and Tainer, 2013	http://www.bioisis.net/
SWISS-MODEL	Biasini et al., 2014	https://swissmodel.expasy.org/
MODELER	Webb and Sali, 2014	https://salilab.org/modeller/
UCSF CHIMERA	Pettersen et al., 2004	https://www.cgl.ucsf.edu/chimera/
AllosMod-FoXS	Guttman et al., 2013	http://modbase.compbio.ucsf.edu/allosmod-foxs/
Scrubber2	BioLogic Software	http://www.biologic.com.au/
BIAevaluation	GE Healthcare	https://www.biacore.com/
Origin ITC	Malvern	https://www.malvern.com/en/
Sedfit	Brown and Schuck, 2006	http://www.analyticalultracentrifugation.com/default.htm
GUSSI	Brautigam, 2015	http://biophysics.swmed.edu/MBR/software.html
Integrated Proteomics Pipeline	Savas et al., 2014	http://www.integratedproteomics.com/
Other		
HisTrap FF	GE Healthcare	Cat# 17-5255-01
Superdex 16/60 200 PG HiLoad	GE Healthcare	Cat# 28989335
QuixStand	GE Healthcare	Cat# 56-4107-78
Biacore T200	GE Healthcare	Cat# 28975001
Sensor Chip CM5	GE Healthcare	Cat# BR100012
EASY-nLC 1000 Liquid Chromatograph	Thermo Scientific	Cat# LC120
Orbitrap Fusion Tribrid Mass Spectrometer	Thermo Scientific	Cat# IQLAAEGAAPFADBMBCX
Acclaim PepMap 100 75 um x 2 cm nanoViper	Thermo Scientific	Cat# 164946
Acclaim PepMap RSLC 75 um x 50 cm nanoViper	Thermo Scientific	Cat# 164942

CONTACT FOR REAGENT AND RESOURCE SHARING

Further information and requests for reagents may be directed to and will be fulfilled by the Lead Contact, A. Radu Aricescu (radu@mrc-lmb.cam.ac.uk).

METHOD DETAILS

Expression and purification of recombinant proteins

List of cDNAs and construct boundaries for secreted protein production: chicken MAM domain-containing glycosylphosphatidylinositol anchor protein (MDGA) 1 (MDGA1; GenBank: AB241390.1; Gln19-Lys919), human MDGA1 (GenBank: NM_153487.3; Gln19-Lys925), human MDGA2 (GenBank: AY369208.1; Gln21-Lys927), human neuroligin-1 (NL1; GenBank: NM_014932.3; Gln46-Asp635), human neuroligin-2 (NL2; GenBank: NM_020795.3; Glu38-His612), human neuroligin-3 (NL3; GenBank: NM_181303; Gln38-Asp636), human neuroligin-4 (NL4 or NL4(X); GenBank: NM_020742.3; Gln42-Glu602), human neuroligin-5 (NL5 or NL4(Y); GenBank: NM_014893.4; Gln42-Glu602), human β-neurexin-1 (GenBank: NM_138735; β-NRX1: His85-Val265), human thrombospondin-1 (TSP1; GenBank: X04665.1; Asn19-Pro1170), human hevin (or SPARC-like protein 1; GenBank: BC033721.1; Ile17-Phe664), mouse α-Neurexin-1 (GenBank: XM_006523816.3; Leu31-Val1337).

These cDNAs were fused C-terminally with a hexa-histidine (His6) tag or Avitag3, and were cloned into the pHLsec vector (Aricescu et al., 2006b). For large-scale protein production, His6-tagged proteins were expressed by transient transfection in HEK293T (for biophysical studies) or HEK293S-GnTI$^{-/-}$ (Reeves et al., 2002) (for crystallographic studies) cells. Five (HEK293T) to ten (HEK293S-GnTI$^{-/-}$) days post-transfection, the conditioned Dulbecco's Modified Eagle Medium (DMEM) medium was collected and buffer-exchanged using a QuixStand benchtop diafiltration system (GE Healthcare) and proteins were purified by immobilized metal-affinity chromatography (IMAC) using pre-packed Nickel Sepharose columns (GE Healthcare). Proteins were concentrated and further purified by size-exclusion chromatography (SEC; Superdex 200 16/60 PG HiLoad column, GE Healthcare) in 10 mM HEPES (4-(2-hydroxyethyl)-1-piperazineethanesulfonic acid) pH 7.50, 150 mM sodium chloride and 3 mM calcium chloride (HBS-C).

Expression and purification of recombinant NMDA receptor

The rat GluN1a-GluN2B heterotetrameric NMDA receptor (NMDAR) was expressed and purified as previously described (Karakas and Furukawa, 2014), with the exception that the OneStrep tag was not cleaved. The final purification buffer was 200 mM NaCl, 20 mM HEPES pH 7.4, 10 mM Glycine, 10 mM Glutamate, 0.0025% LMNG.

Gene splicing and site-directed mutagenesis

A multiple-step overlap-extension PCR (Pyrobest Polymerase, Takara Bio) was used for site-directed mutagenesis, construction of chimeric protein constructs and introduction or deletion of splice inserts (Heckman and Pease, 2007); the resulting PCR products were cloned into the pHLsec-His6, pHLsec-Avitag3, or derived vectors (Aricescu et al., 2006b).

NL1

The following internal primer pair was used for the introduction of human NL1 spliced sequence "A1" (VKRISKECARKPGKKICRKG) into human NL1(–A ± B) (UniProt: Q8N2Q7; between Asp164 and Asp182);

FP: 5′-CCAAGGAATGTGCCAGAAAGCCCGGCAAGAAAATATGTAGAAAAGGAGATATTCGGGACAGTGGGGGTCCCAAACCAG-3′
RP: 5′-CTTGCCGGGCTTTCTGGCACATTCCTTGGATATTCTTTTTACATCCTCAGTCGGGACATATATATTTAAATATAG-3′

The following internal primer pair was used for the introduction of human NL1 spliced sequence "A2" (GPLTKKQTDDLGDNDGAEDE) into human NL1(–A ± B) (between Asp164 and Asp182);

FP: 5′-GAAACAGACAGATGATTTAGGTGATAATGACGGTGCTGAAGATGAAGATATTCGGGACAGTGGGGGTCCCAAACCAG-3′
RP: 5′-CTTGCCGGGCTTTCTGGCACATTCCTTGGATATTCTTTTTACATCCTCAGTCGGGACATATATATTTAAATATAG-3′

The following internal primer pair was used for the introduction of human NL1 spliced sequence "A2" (GPLTKKQTDDLGDNDGAEDE) into human NL1(+A1 ± B) (after spliced sequence A1);

FP: 5′-GAAACAGACAGATGATTTAGGTGATAATGACGGTGCTGAAGATGAAGATATTCGGGACAGTGGGGGTCCCAAACCAG-3′
RP: 5′-CATTATCACCTAAATCATCTGTCTGTTTCTTTGTAAGGGGACCTCCTTTTCTACATATTTTCTTGCCGGGCTTTC-3′

The following internal primer pair was used for the introduction of human NL1 spliced sequence "B" (NRWSNSTKG) into human NL1(±A–B) (between Gly295 and Leu305);

FP: 5′-GTAACCGTTGGAGCAATTCAACCAAAGGACTTTTTCAACGAGCAATAGCTCAAAG-3′
RP: 5′-GTCCTTTGGTTGAATTGCTCCAACGGTTACCTTCAGAATAATGGGATAAAGTC-3′

The following internal primer pairs were used for constructing the human NL1 ΔSite I mutant (H291A-Y292A-D384A-E394A);

H291A Y292A (LTLSHYSEGL to LTLSAASEGL);
FP: 5′-GTCAACCTGCTGACTTTATCCGCTGCTTCTGAAGGTCTTTTTCAACGAG-3′
RP: 5′-CTCGTTGAAAAAGACCTTCAGAAGCAGCGGATAAAGTCAGCAGGTTGAC-3′
D384A (VIPDDPQI to VIPADPQI);
FP: 5′-GGTGATGTAATACCAGCCGACCCCCAGATATTG-3′
RP: 5′-CAATATCTGGGGGTCGGCTGGTATTACATCACC-3′

E394A (MEQGEFLNY to MEQGAFLNY);
FP: 5′-GATGGAGCAAGGAGCGTTTCTCAACTATG-3′
RP: 5′-CATAGTTGAGAAACGCTCCTTGCTCCATC-3′

The following internal primer pairs were used for constructing the human NL1 ΔSite II mutant (D429A-F430A-S433A-N434A-R450A);
D429A F430A S433A N434A (ASDFDFAVSNFVDN to ASDFAAAVAAFVDN);
FP: 5′-GCCGCTGCTGTTGCAGCTTTTGTTGATAATTTATATGGATATCCTGAAGGCAAAGATG-3′
RP: 5′-AGCTGCAACAGCAGCGGCAAAATCACTAGCTGATATACCATCATCGCTATCTAC-3′
R450A (KDVLRETIK to KDVLAETIK);
FP: 5′-GAAGGCAAAGATGTTTTGGCAGAAACCATTAAGTTCATG-3′
RP: 5′-CATGAACTTAATGGTTTCTGCCAAAACATCTTTGCCTTC-3′

The following internal primer pair was used for introducing the R450C mutation into human NL1(–A–B) (DVLRETI to DVLCETI);
FP: 5′-GGCAAAGATGTTTTGTGCGAAACCATTAAGTTC-3′
RP: 5′-GAACTTAATGGTTTCGCACAAAACATCTTTGCC-3′

The following internal primer pair was used for introducing the Asn300Gln (N300Q) mutation into human NL1 spliced sequence “B” (NRWSNSTKG to NRWSQSTKG);
FP: 5′-GGTAACCGTTGGAGCCAGTCAACCAAAGGAC-3′
RP: 5′-GTCCTTTGGTTGACTGGCTCCAACGGTTACC-3′

NL2

The following internal primer pair was used for the introduction of human NL2 spliced sequence “A” (GPLTKKRDEATLNPPDT) into human NL2(–A) (UniProt: Q8NFZ4; between Asp152 and Asp170);
FP: 5′-CACAAAAAAACGTGACGAGGCGACGCTCAATCCGCCAGACACAGATATCCGGGACCCTGGGAAGAAACCTGTC-3′
RP: 5′-GATTGAGCGTCGCCTCGTCACGTTTTTTTGTGAGCGGACCGTCCTCAGTGGGCACGTAGAGGTTGAGGTAC-3′

NL3

The following internal primer pair was used for the introduction of human NL3 spliced sequence “A1” (VKRISKECARKPNKKICRKG) into human NL3(–A) (UniProt: Q9NZ94; between Asp152 and Asp193);
FP: 5′-CCAAGGAATGCGCCCGAAAGCCCAACAAGAAAATTTGTAGGAAAGGAGACATCCGGGACAGTGGTGCTAAACCCGTC-3′
RP: 5′-GTTGGGCTTTCGGGCGCATTCCTTGGAAATCCGCTTTACATCCTCCGTCGGCACATAGACGTTCAGGTAG-3′

The following internal primer pair was used for the introduction of human NL3 spliced sequence “A2” (GSGAKKQGEDLADNDG DEDE) into human NL3(–A) (between Asp152 and Asp193);
FP: 5′-GAAACAGGGCGAGGACTTAGCGGATAATGACGGGGATGAAGATGAAGACATCCGGGACAGTGGTGCTAAACCCGTC –3′
RP: 5′-CCGCTAAGTCCTCGCCCTGTTTCTTAGCGCCGGATCCATCCTCCGTCGGCACATAGACGTTC-3′

The following internal primer pair was used for the introduction of human NL3 spliced sequence “A2” (GSGAKKQGEDLADNDG DEDE) into human NL3(+A1) (after spliced sequence A1);
FP: 5′-GAAACAGGGCGAGGACTTAGCGGATAATGACGGGGATGAAGATGAAGACATCCGGGACAGTGGTGCTAAACCCGTC-3′
RP: 5′-CATTATCCGCTAAGTCCTCGCCCTGTTTCTTAGCGCCGGATCCTCCTTTCCTACAAATTTTCTTGTTGGGCTTTC-3′

The following internal primer pair was used for introducing the R451C mutation into human NL3(–A) (DTLRETI to DTLCETI);
FP: 5′-GGTAAGGACACCCTGTGCGAGACCATCAAGTTC-3′
RP: 5′-GAACTTGATGGTCTCGCACAGGGTGTCCTTACC-3′

MDGA1

The following internal primer pair was used for introducing the R120K mutation into chicken MDGA1 (UniProt: Q0WYX8; VPAIRSIRV to VPAIKSIRV);
FP: 5′-GTTGGGGTCCCTGCCATCAAGTCCATTCGAGTAGATGTGCAG-3′
RP: 5′-CTGCACATCTACTCGAATGGACTTGATGGCAGGGACCCCAAC-3′

The following internal primer pairs were used for constructing the chicken MDGA1 R156N-S502N-R680N glycan wedge mutant;
R156N (TVFLRCTVN to TVFLNCTVN);
FP: 5′-GAGAAGACTGTCTTCCTCAATTGTACCGTCAACTCCAAC-3′
RP: 5′-GTTGGAGTTGACGGTACAATTGAGGAAGACAGTCTTCTC-3′
S502N (LRLESVSRD to LRLENVSRD);
FP: 5′-GGAAGCTGCGCCTGGAGAATGTCAGCCGAGACATGAG-3′
RP: 5′-CTCATGTCTCGGCTGACATTCTCCAGGCGCAGCTTCC-3′
R680N (LAQRNTIQ to LAQNNTIQ);
FP: 5′-GTCAGGCAGCTGGCTCAGAACAACACCATCCAAACCTTC-3′
RP: 5′-GAAGGTTTGGATGGTGTTGTTCTGAGCCAGCTGCCTGAC-3′

β-NRX1

The following internal primer pair was used for the introduction of human β-NRX1 spliced sequence #4 (SS4; GNNDNERLAIARQRIPYRLGRVVDEWLLDK) into human β-NRX1(–4) (UniProt: P58400; between Ala204 and Gly205);
FP: 5′-CGCATTCCCTATCGGCTAGGGAGAGTGGTGGACGAATGGCTGCTCGATAAAGGGAGGCAACTGACCATCTTCAACTCAC-3′
RP: 5′-CCCTAGCCGATAGGGAATGCGTTGCCGTGCTATGGCTAACCTCTCATTGTCGTTGTTTCCAGCTGGGTATCTCTCAATGAC-3′

TSP1

The following internal primer pair was used for introducing the C992S mutation (Kvansakul et al., 2004) into human TSP1 (UniProt: P07996; QTVNCDPGL to QTVNSDPGL);

FP: 5′-CAGACTGTCAACAGTGATCCTGGACTC-3′

RP: 5′-GAGTCCAGGATCACTGTTGACAGTCTG-3′

Protein crystallization

Crystallization trials, using 100 nL protein solution plus 100 nL reservoir solution in sitting drop vapor diffusion format, were set up in 96-well Greiner plates using a Cartesian Technologies robot (Walter et al., 2005).

Purified chicken MDGA1$_{ECTO}$ (cMDGA1$_{ECTO}$; Gln19-Lys919), containing the Arg120Lys mutation, concentrated to 5.0 g/L and treated with endoglycosidase F1 (Endo F1; 1:100 w/w) for 30 min at 294K immediately prior to dispensing the crystallization drops, crystallized in 0.1M HEPES pH7.5, 4% w/v polyethylene glycol 8000. The Arg120Lys mutation was introduced into cMDGA1$_{ECTO}$ to bring the sequence in line with rat, mouse and human isoforms (Figure S1B).

Crystals of cMDGA1$_{ECTO}$ grown in this condition were fragmented, and the obtained seed stock (Walter et al., 2008) was used as an additive during crystallization trials of selenomethionine- (SeMet) labeled cMDGA1$_{ECTO}$. Matrix screens were performed using precipitant concentration and seed stock dilution as variables. SeMet-labeled cMDGA1$_{ECTO}$, concentrated to 5.0 g/L, crystallized in 0.1M HEPES pH7.5, 3% w/v polyethylene glycol 8000, using a 32-fold diluted native cMDGA1$_{ECTO}$ seed stock dispensed in 20 nL drops. Crystals were cryoprotected using reservoir solution containing 20% (v/v) PEG200.

Purified glycosylated human NL1(–A+B)$_{ECTO}$ (hNL1(–A+B)$_{ECTO}$; Gln46-Asp635), concentrated to 10.0 g/L, crystallized in 0.2M KSCN, 0.1M Bis-tris propane pH 8.5, 20% w/v PEG3350. Crystals were cryoprotected using reservoir solution containing 20% (v/v) PEG200.

To crystallize the hNL1(–A–B)$_{ECTO}$–cMDGA1$_{ECTO}$ complex, purified hNL1(–A–B)$_{ECTO}$ (Gln46-Asp635; concentrated to 2.92 g/L = 45.30 μM) and cMDGA1$_{ECTO}$ (Gln19-Lys919 with Arg120Lys mutation; concentrated to 4.41 g/L = 42.94 μM) were mixed as follows; 80 μL hNL1(–A–B)$_{ECTO}$ was combined with 102 μL cMDGA1$_{ECTO}$ (resulting in a 1:1.25 NL1:MDGA1 monomer-to-monomer molar stoichiometric ratio), 18 μL purification buffer (10 mM HEPES pH7.5, 150 mM NaCl), and 50 μL dilution buffer (10 mM HEPES pH7.5, 150 mM NaCl, 1M NDSB-256). The final concentration of hNL1(–A–B)$_{ECTO}$ thus was 0.93 g/L; that of cMDGA1$_{ECTO}$ was 1.76 g/L; and that of NDSB-256 was 250 mM. This preparation was treated with endoglycosidase F1 (Endo F1; 1:100 w/w) for 30 min at 294K immediately prior to dispensing the crystallization drops. The hNL1(–A–B)$_{ECTO}$–cMDGA1$_{ECTO}$ complex crystallized in 0.1M Na.HEPES pH 7.0, 7.5% w/v PEG8000. Crystals were cryoprotected using reservoir solution containing 33% (v/v) PEG200.

Crystallographic data collection and structure determination

Diffraction data for cMDGA1$_{ECTO}$ were collected at Diamond Light Source (DLS) beamline I03 to a nominal resolution of 3.20 Å in space group (SG) $P2_12_12_1$. X-ray fluorescence wavelength scans were performed to experimentally determine the Selenium absorption K-edge peak. The cMDGA1$_{ECTO}$ structure was determined using Single Anomalous Diffraction (SAD); the heavy-atom Selenium substructure was solved using SHELXD (Schneider and Sheldrick, 2002) at 3.70 Å, and phase determination, phase extension and density modification was performed using PHENIX Autosol (Terwilliger et al., 2009). Automated model building programs failed to reliably place stretches of β strand, necessitating manual model building of the complete structure.

Diffraction data for hNL1(–A+B)$_{ECTO}$ were collected at Diamond Light Source (DLS) beamline I24 to a nominal resolution of 2.55 Å in SG $P22_12_1$. The structure was determined by molecular replacement using the program Phaser (McCoy et al., 2007), and using the mouse NL1 (PDB: 3BIX) crystal structure as search model.

Diffraction data for the hNL1(–A–B)$_{ECTO}$–cMDGA1$_{ECTO}$ complex were collected at Diamond Light Source (DLS) beamline I04-1 to a nominal resolution of 3.30 Å in SG $P2_12_12$. The structure was determined by molecular replacement using the program Phaser (McCoy et al., 2007), employing the refined hNL1(–A+B)$_{ECTO}$ (in which the spliced sequence B was excised from the molecular model) and cMDGA1$_{ECTO}$ crystal structures we determined here as search models.

All data were indexed, integrated, and scaled using the automated XIA2 expert system (Winter et al., 2013), using the Labelit (Sauter et al., 2004), POINTLESS and AIMLESS (Evans, 2006, 2011), and XDS (Kabsch, 2010) programs. Crystallographic data collection and refinement statistics are presented in Table S1.

Crystallographic refinement and model analysis

Maximum-likelihood refinement of cMDGA1$_{ECTO}$, hNL1(–A+B)$_{ECTO}$ and the hNL1(–A–B)$_{ECTO}$–cMDGA1$_{ECTO}$ complex was initially performed with Refmac using "jelly body" restraints (Murshudov et al., 2011), and finally with the PHENIX suite (Adams et al., 2010), with automated X-ray and atomic displacement parameter (ADP) weight optimization applied throughout, and torsion angle non-crystallographic symmetry (NCS) and high-resolution reference structure restraints applied where suitable. All manual model building was performed using Coot (Emsley et al., 2010). Structure validation was performed with the PHENIX program suite using MolProbity routines (Adams et al., 2010; Chen et al., 2010).

Interface analysis was performed using PISA (Krissinel and Henrick, 2007) as implemented in Coot (Emsley et al., 2010), and using the program Intervor (Loriot and Cazals, 2010). Calculation of pairwise root-mean-square deviations (rmsd) between structural model

coordinates was performed using the program PyMol (Schrodinger, 2010). Molecular representations were made using the program PyMol (Schrodinger, 2010).

Sequence alignments and conservation analysis

Mining of protein sequence databases was performed using the Delta-Blast program (Altschul et al., 1990). Sequence lists were manually curated and sequences were aligned using the program MUSCLE (Edgar, 2004). Sequence conservation scores for individual residue positions of NL1, −2, −3, −4, and −5 (1046 total unique sequences) and MDGA1 and −2 (420 total unique sequences) homologs were assigned to NL1 and MDGA1 structural templates extracted from the hNL1(–A–B)$_{ECTO}$–cMDGA1$_{ECTO}$ complex, respectively, using the ConSurf web server (Ashkenazy et al., 2010). Sequence alignments were visualized using the program ALINE (Bond and Schüttelkopf, 2009).

Small-angle X-ray scattering (SAXS)

Purified cMDGA1$_{ECTO}$ (Gln19-Lys919; with Arg120Lys mutation) was treated with endoglycosidase F1 (Endo F1; 1:100 w/w) for 12 hr at 294K and re-purified using SEC in 10 mM HEPES pH 7.50, 150 mM NaCl. SAXS data were collected at beamline BM29 of the European Synchrotron Radiation Facility (ESRF, Grenoble, France) (Pernot et al., 2013) at 293 K within a momentum transfer (q) range of 0.01 $Å^{-1} < q < 0.45$ $Å^{-1}$, where $q = 4\pi\sin(\theta)/\lambda$, and 2θ is the scattering angle. The X-ray wavelength was 0.9950 Å, and data were collected on a Pilatus 1M detector. cMDGA1$_{ECTO}$ was measured at concentrations of 1.50 and 3.36 g/L in 10 mM HEPES pH 7.50, 150mM NaCl. Data reduction and calculation of invariants was carried out using standard procedures implemented in the ATSAS (Petoukhov et al., 2012) and ScÅtter (Rambo and Tainer, 2013) suites. A merged dataset was obtained by merging the low-angle part of the low-concentration dataset with the high-angle part of the high-concentration dataset.

A molecular model for the C-terminal Mam$_8$ domain was generated by homology modeling starting from the crystal structure of the N-terminal RPTPmu MAM domain (PDB: 2C9A, UniProt: P28827) (Aricescu et al., 2006a) using the SWISS-MODEL server (Biasini et al., 2014). This model was concatenated with the cMDGA1$_{ECTO}$ crystal structure, and manually placed near the C terminus of the FnIII$_7$ domain. Missing side chains, loops, and C-terminal His6-tag were added to the resulting assembled model using the MODELER (Webb and Sali, 2014) "Model/Refine Loops" routine as implemented in Chimera (Pettersen et al., 2004).

Coarse-grained molecular dynamics (MD) simulations were performed using the program Allosmod (Weinkam et al., 2012). Five independent runs were performed, each consisting of 30 independent trajectories generating 100 models. From this total pool of 15,000 models, automated selection of the minimal set of models that best described the scattering data was performed with the program MES (Hammel, 2012), and calculation and fitting of scattering patterns were performed with the program FoXS (Schneidman-Duhovny et al., 2013). This whole procedure was automated with the AllosMod-FoXS web server (Guttman et al., 2013). The MDGA1 solution structure was accurately ($\chi^2 = 1.17$) modeled as a five-membered ensemble of monomeric conformers with pronounced flexibility at the FnIII$_7$-Mam$_8$ domain linkage.

Surface plasmon resonance (SPR) with soluble proteins

cDNA for the immobilized proteins was cloned into the pHLsec-Avitag3 vector (Aricescu et al., 2006b), resulting in proteins carrying a C-terminal biotin ligase (BirA) recognition sequence (Avitag). Constructs were co-transfected with pDisplay-BirA-ER (Addgene plasmid 20856; coding for an ER-resident biotin ligase) (Howarth et al., 2008) for in vivo biotinylation in HEK293T cells in small-scale 6- or 12-well plates in a 3:1 pHLsec:pDisplay stoichiometric ratio. A final concentration of 100 μM D-biotin was maintained in the expression medium to ensure near-complete biotinylation of the recognition sequence. After 48 hr of expression, conditioned medium was collected and dialysed against 10 mM Tris pH 7.4, 150 mM sodium chloride, 3 mM calcium chloride and 0.005% (v/v) Tween-20 (TBS-CT). SPR experiments were performed on a Biacore T200 machine (GE Healthcare) operated at a data collection frequency of 10 Hz; i.e. a temporal resolution of 0.1 s. Streptavidin (Sigma-Aldrich) was chemically coupled via amine coupling chemistry onto CM5 chips to a response unit (RU) level of 5000 RU. Then, biotinylated proteins were captured to the desired RU level. In each instance, for every two analyte binding cycles, a buffer injection was performed, allowing for double referencing of the binding responses (Myszka, 1999).

Due to (i) sample consumption associated with equilibrium affinity experiments of high-nanomolar to low-micromolar interactions and (ii) the limited production yield of MDGA1 and −2 proteins, we prioritized testing the full matrix of NL–MDGA isoform interactions over performing replicate experiments of only a selected number of interactions.

Interaction of chicken MDGA1$_{ECTO}$ with chicken MDGA1$_{ECTO}$ and MDGA1$_{ECTO}^{GLYCAN\ WEDGE}$

cMDGA1$_{ECTO}$ and cMDGA1$_{ECTO}^{GLYCAN\ WEDGE}$ (triple glycan wedge (GW) mutant; Arg680Asn-Ser502Asn-Arg156Asn) variants were immobilized at a level of 2000 RU to maximize the likelihood of detecting a potentially weak binding event. SPR running buffer composition was TBS-CT supplemented with 1.0 g/L bovine serum albumin (BSA; yielding TBS-CTB buffer) as passivating agent to prevent binding to the carboxymethyldextran-based SPR chips. MDGA1$_{ECTO}$ was prepared by SEC in TBS-CT. BSA was added to the concentrated stock solutions to a final concentration of 1.0 g/L. Injection of 18 concentrations of cMDGA1$_{ECTO}$ prepared in a two-fold dilution series from a 100 μM stock concentration was performed in order of increasing concentration. Each sample was injected for 150 s at a flow rate of 25 μL/min, followed by a 180 s dissociation phase. No self-association binding event could be detected.

Interaction of human β-NRX1$_{LNS6}$(–4), β-NRX1$_{LNS6}$(+4), MDGA1$_{ECTO}$ and MDGA2$_{ECTO}$ with human NL1(–A–B)$_{ECTO}$, NL2(–A)$_{ECTO}$, NL3(–A)$_{ECTO}$, NL4$_{ECTO}$ and NL5$_{ECTO}$

NL1(–A–B)$_{ECTO}$, NL2(–A)$_{ECTO}$, NL3(–A)$_{ECTO}$, NL4$_{ECTO}$ and NL5$_{ECTO}$ were immobilized at a level of 500 RU. SPR running buffer composition was TBS-CTB. β-NRX1$_{LNS6}$(–4), β-NRX1$_{LNS6}$(+4), MDGA1$_{ECTO}$ and MDGA2$_{ECTO}$ were prepared by SEC in TBS-CT. BSA was added to the concentrated stock solutions to a final concentration of 1.0 g/L. Injection of 15 concentrations of β-NRX1$_{LNS6}$(–4), β-NRX1$_{LNS6}$(+4), MDGA1$_{ECTO}$ and MDGA2$_{ECTO}$, prepared in a two-fold dilution series from a 50 μM stock concentration, was performed in order of increasing concentration. Each sample was injected for 150 s at a flow rate of 25 μL/min, followed by a 180 s dissociation phase. In the case of MDGA1$_{ECTO}$ and MDGA2$_{ECTO}$, the surfaces were regenerated using consecutive 30 s injections of 10 mM Tris pH 7.4, 100 mM L-Arginine/L-Glutamate, 1M NaCl. Equilibrium binding analysis was performed using Scrubber 2.0 (BioLogic Software) and data was fitted to a 1:1 Langmuir binding model in Prism 6 (Graphpad).

Interaction of human NL1(–A–B)$_{ECTO}$ with human hevin, human TSP1, and mouse α-NRX1$_{ECTO}$(–4)

Human hevin, human TSP1 and mouse α-NRX1$_{ECTO}$(–4) were immobilized at a level of 2000 RU. SPR running buffer composition was TBS-CTB. Human NL1(–A–B)$_{ECTO}$ was prepared by SEC in TBS-CT. BSA was added to the concentrated stock solutions to a final concentration of 1.0 g/L. Injection of 14 concentrations of NL1(–A–B)$_{ECTO}$, prepared in a two-fold dilution series from a 25 μM stock concentration, was performed in order of increasing concentration. Each sample was injected for 150 s at a flow rate of 25 μL/min, followed by a 180 s dissociation phase. In the case of the interaction with α-NRX1$_{ECTO}$(–4), the surfaces were regenerated using a 30 s injection of 10 mM Tris pH 8.0, 350 mM EDTA, 100 mM NaCl. Equilibrium binding analysis was performed using Scrubber 2.0 (BioLogic Software) and the α-NRX1$_{ECTO}$(–4) data was fitted to a two-state Langmuir binding model in Prism 6 (Graphpad).

Interaction of human β-NRX1$_{LNS6}$(–4), β-NRX1$_{LNS6}$(+4), MDGA1$_{ECTO}$ and MDGA2$_{ECTO}$ with human NL1(–A–B)$_{ECTO}$, NL1(–A–B)$_{ECTO}$ ΔSite I, NL1(–A–B)$_{ECTO}$ ΔSite II and NL1(–A–B)$_{ECTO}$ ΔSite I+II

NL1(–A–B)$_{ECTO}$, NL1(–A–B)$_{ECTO}$ ΔSite I, NL1(–A–B)$_{ECTO}$ ΔSite II and NL1(–A–B)$_{ECTO}$ ΔSite I+II were immobilized at a level of 550 RU. SPR running buffer composition was TBS-CTB. β-NRX1$_{LNS6}$(–4), β-NRX1$_{LNS6}$(+4), MDGA1$_{ECTO}$ and MDGA2$_{ECTO}$ were prepared by SEC in TBS-CT. BSA was added to the concentrated stock solutions to a final concentration of 1.0 g/L. Injection of 15 concentrations of β-NRX1$_{LNS6}$(–4), β-NRX1$_{LNS6}$(+4), MDGA1$_{ECTO}$ and MDGA2$_{ECTO}$, prepared in a two-fold dilution series from a 50 μM stock concentration, was performed in order of increasing concentration. Each sample was injected for 150 s at a flow rate of 25 μL/min, followed by a 180 s dissociation phase. In the case of MDGA1$_{ECTO}$ and MDGA2$_{ECTO}$, the surfaces were regenerated using consecutive 30 s injections of 10 mM Tris pH 7.4, 100 mM L-Arginine/L-Glutamate, 1M NaCl. Equilibrium binding analysis was performed using Scrubber 2.0 (BioLogic Software) and data was fitted to a 1:1 Langmuir binding model in Prism 6 (Graphpad).

Interaction of human β-NRX1$_{LNS6}$(–4), β-NRX1$_{LNS6}$(+4), MDGA1$_{ECTO}$ and MDGA2$_{ECTO}$ with human NL1(–A–B)$_{ECTO}$, NL1(–A–B)$_{ECTO}$ Arg450Cys, NL3(–A)$_{ECTO}$, and NL3(–A)$_{ECTO}$ Arg451Cys

NL1(–A–B)$_{ECTO}$ and NL1(–A–B)$_{ECTO}$ Arg450Cys were immobilized at a level of 500 RU. NL3(–A)$_{ECTO}$ and NL3(–A)$_{ECTO}$ Arg451Cys were immobilized at a level of 1000 RU. SPR running buffer composition was TBS-CTB. β-NRX1$_{LNS6}$(–4), β-NRX1$_{LNS6}$(+4), MDGA1$_{ECTO}$ and MDGA2$_{ECTO}$ were prepared by SEC in TBS-CT. BSA was added to the concentrated stock solutions to a final concentration of 1.0 g/L. Injection of 15 concentrations of β-NRX1$_{LNS6}$(–4), β-NRX1$_{LNS6}$(+4), MDGA1$_{ECTO}$ and MDGA2$_{ECTO}$, prepared in a two-fold dilution series from a 50 μM stock concentration, was performed in order of increasing concentration. Each sample was injected for 150 s at a flow rate of 25 μL/min, followed by a 180 s dissociation phase. In the case of MDGA1$_{ECTO}$ and MDGA2$_{ECTO}$, the surfaces were regenerated using consecutive 30 s injections of 10 mM Tris pH 7.4, 100 mM L-Arginine/L-Glutamate, 1M NaCl. Equilibrium binding analysis was performed using Scrubber 2.0 (BioLogic Software) and data was fitted to a 1:1 Langmuir binding model in Prism 6 (Graphpad).

Interaction of human β-NRX1$_{LNS6}$(–4), β-NRX1$_{LNS6}$(+4), MDGA1$_{ECTO}$ and MDGA2$_{ECTO}$ with human NL1$_{ECTO}$ SSA variants

NL1(–A–B)$_{ECTO}$, NL1(+A1–B)$_{ECTO}$, NL1(+A2–B)$_{ECTO}$ and NL1(+A1+A2–B)$_{ECTO}$ were immobilized at a level of 500 RU. SPR running buffer composition was TBS-CTB. β-NRX1$_{LNS6}$(–4), β-NRX1$_{LNS6}$(+4), MDGA1$_{ECTO}$ and MDGA2$_{ECTO}$ were prepared by SEC in TBS-CT. BSA was added to the concentrated stock solutions to a final concentration of 1.0 g/L. Injection of 15 concentrations of β-NRX1$_{LNS6}$(–4), β-NRX1$_{LNS6}$(+4), MDGA1$_{ECTO}$ and MDGA2$_{ECTO}$, prepared in a two-fold dilution series from a 50 μM stock concentration, was performed in order of increasing concentration. Each sample was injected for 150 s at a flow rate of 25 μL/min, followed by a 180 s dissociation phase. In the case of MDGA1$_{ECTO}$ and MDGA2$_{ECTO}$, the surfaces were regenerated using consecutive 30 s injections of 10 mM Tris pH 7.4, 100 mM L-Arginine/L-Glutamate, 1M NaCl. Equilibrium binding analysis was performed using Scrubber 2.0 (BioLogic Software) and data was fitted to a 1:1 Langmuir binding model in Prism 6 (Graphpad).

Interaction of human β-NRX1$_{LNS6}$(–4), β-NRX1$_{LNS6}$(+4), MDGA1$_{ECTO}$ and MDGA2$_{ECTO}$ with human NL2$_{ECTO}$ and NL3$_{ECTO}$ SSA variants

NL2(–A)$_{ECTO}$, NL2(+A)$_{ECTO}$, NL3(–A)$_{ECTO}$, NL3(+A1)$_{ECTO}$, NL3(+A2)$_{ECTO}$ and NL3(+A1+A2)$_{ECTO}$ were immobilized at a level of 500 RU. SPR running buffer composition was TBS-CTB. β-NRX1$_{LNS6}$(–4), β-NRX1$_{LNS6}$(+4), MDGA1$_{ECTO}$ and MDGA2$_{ECTO}$ were prepared by SEC in TBS-CT. BSA was added to the concentrated stock solutions to a final concentration of 1.0 g/L. Injection of 15 concentrations of β-NRX1$_{LNS6}$(–4), β-NRX1$_{LNS6}$(+4), MDGA1$_{ECTO}$ and MDGA2$_{ECTO}$, prepared in a two-fold dilution series from a 50 μM stock concentration, was performed in order of increasing concentration. Each sample was injected for 150 s at a flow rate of 25 μL/min, followed by a 180 s dissociation phase. In the case of MDGA1$_{ECTO}$ and MDGA2$_{ECTO}$, the surfaces were regenerated using consecutive

30 s injections of 10 mM Tris pH 7.4, 100 mM L-Arginine/L-Glutamate, 1M NaCl. Equilibrium binding analysis was performed using Scrubber 2.0 (BioLogic Software) and data was fitted to a 1:1 Langmuir binding model in Prism 6 (Graphpad).

Interaction of human β-NRX1$_{LNS6}$(–4), β-NRX1$_{LNS6}$(+4), MDGA1$_{ECTO}$ and MDGA2$_{ECTO}$ with human NL1(–A–B)$_{ECTO}$, NL1(–A+B)$_{ECTO}$, and NL1(–A+B Asn300Gln)$_{ECTO}$

NL1(–A–B)$_{ECTO}$, NL1(–A+B)$_{ECTO}$, and NL1(–A+B Asn300Gln)$_{ECTO}$ were immobilized at a level of 500 RU. SPR running buffer composition was TBS-CTB. β-NRX1$_{LNS6}$(–4), β-NRX1$_{LNS6}$(+4), MDGA1$_{ECTO}$ and MDGA2$_{ECTO}$ were prepared by SEC in TBS-CT. BSA was added to the concentrated stock solutions to a final concentration of 1.0 g/L. Injection of 15 concentrations of β-NRX1$_{LNS6}$(–4), β-NRX1$_{LNS6}$(+4), MDGA1$_{ECTO}$ and MDGA2$_{ECTO}$, prepared in a two-fold dilution series from a 50 μM stock concentration, was performed in order of increasing concentration. Each sample was injected for 150 s at a flow rate of 25 μL/min, followed by a 180 s dissociation phase. In the case of MDGA1$_{ECTO}$ and MDGA2$_{ECTO}$, the surfaces were regenerated using consecutive 30 s injections of 10 mM Tris pH 7.4, 100 mM L-Arginine/L-Glutamate, 1M NaCl. Equilibrium binding analysis was performed using Scrubber 2.0 (BioLogic Software) and data was fitted to a 1:1 Langmuir binding model in Prism 6 (Graphpad).

Surface plasmon resonance (SPR) with the NMDA receptor

SPR experiments were performed on a Biacore T200 machine (GE Healthcare) operated at a data collection frequency of 10 Hz; i.e. a temporal resolution of 0.1 s. Streptactin XT (IBA Lifesciences) was chemically coupled via amine coupling chemistry onto CM5 chips to a response unit (RU) level of 5000 RU. Then, OneStrep-tagged rat GluN1a-GluN2B heterotetrameric NMDA receptor (NMDAR) was captured to a level of 5000 RU. SPR running buffer composition was 200 mM NaCl, 20 mM HEPES pH 7.4, 10 mM Glycine, 10 mM Glutamate, 3mM $CaCl_2$, 0.010% LMNG. For the interaction with NL1(–A–B)$_{ECTO}$, a single-cycle kinetics (SCK) approach was adopted. Injection of 5 concentrations of NL1(–A–B)$_{ECTO}$, prepared in a two-fold dilution series from a 25 μM stock concentration, was performed in order of increasing concentration. Each sample was injected for 120 s at a flow rate of 25 μL/min, followed by a 60 s intermittent dissociation phase or a final 600 s dissociation phase.

Isothermal titration calorimetry (ITC)

Calorimetric measurements were carried out using samples purified by SEC in HBS-C buffer (10 mM HEPES pH 7.50, 150 mM sodium chloride and 3 mM calcium chloride). Experiments were carried out using a VP-ITC MicroCalorimeter (GE Healthcare) at 295 K, and data were analyzed using the Origin ITC analysis software package. Titrations were always preceded by an initial injection of 3 μL and were carried out using sequential 10 μL injections with continuous stirring. The data were fitted to the "one binding site model" and apparent molar reaction enthalpy ($\Delta H°$), apparent entropy ($\Delta S°$), association constant (K_A), and binding stoichiometry (N) was determined.

Analytical ultracentrifugation (AUC)

Sedimentation velocity (SV) experiments were performed using a Beckman Optima XL-I analytical centrifuge operated at a run temperature of 293K. Human MDGA1$_{ECTO}$ was concentrated to 60 μM (6.31 g/L) in TBS-CT buffer. Samples were held in Epon sector-shaped 2-channel centerpieces (6 mm path length) and were spun at 40,000 rpm. 200 sample distribution scans were taken incrementally, spaced 4 min apart. Data were collected using 280 nm absorbance optics.

Data were analyzed using the program Sedfit (Brown and Schuck, 2006). Scans 7-200 were used in the continuous c(s) distribution analysis. Analysis was performed with a floating frictional ratio and baseline, S_{MIN} = 0.0, S_{MAX} = 20, and a resolution value of 100. A value of 0.73 mL/g was used for the partial specific volumes. A buffer density value of 1.00527 g/cm^3 and buffer viscosity value of 0.01022 Poise was calculated using the Sednterp online application. Figures were prepared using the program GUSSI (Brautigam, 2015).

Co-culture and immunocytochemistry

Constructs of the native, full-length (FL) human MDGA1 (Gln19-Arg955), and human MDGA2 (Gln21-Arg956), fused N-terminally with a HA epitope tag, and without C-terminal tags (yielding HA-MDGA1-2$_{FL}$), were cloned into the pHLsec vector (Aricescu et al., 2006b).

Constructs of the full-length human NL1 (–A ± B, –A ± B_Asn300Gln, ΔSite I, ΔSite II, ΔSite I+II, –A–B_Arg450Cys; Gln46-Val840), human NL2 (–A; Glu38-Val835), human NL3 (–A, –A_Arg451Cys; Gln38-Val848), and human NL4 (NL4(X); Gln42-Val816), fused N-terminally with a Myc epitope tag and fused C-terminally with ECFP (enhanced cyan fluorescent protein; yielding myc-NL1-4$_{FL}$), were cloned into the pHLsec vector (Aricescu et al., 2006b). NL1(–A–B)$_{FL}$_Arg450Cys and NL3(–A)$_{FL}$_Arg451Cys used native signal sequences and were not C-terminally fused to ECFP, to avoid the potential impact of these modifications on surface trafficking of the mutants.

Low density primary hippocampal cultures were prepared from E18 rat embryos as previously described (Kaech and Banker, 2006) and as approved by the University of British Columbia Animal Care Committee. Neuron cultures were maintained in Neurobasal (NB) medium (Thermo Fisher Scientific) supplemented with GlutaMAX-I (Thermo Fisher Scientific), B-27 supplement (Thermo Fisher Scientific) and 100 μM APV (Abcam). COS-7 cells were cultured in DMEM supplemented with 10% bovine growth serum and 100 I.U./mL penicillin-streptomycin. Cells were transfected using TransIT-LT1 transfection reagent (Mirus Bio) with (i) 0.5 μg myc-NL1-4$_{FL}$ and 1.1 μg HA-CD4, 1.1 μg HA-MDGA1$_{FL}$, or 1.6 μg HA-MDGA2$_{FL}$ for low ratio experiments; (ii) 0.5 μg myc-NL1-4$_{FL}$ and 1.75 μg HA-CD4, 1.75 μg HA-MDGA1$_{FL}$, or 2.5 μg HA-MDGA2$_{FL}$ for medium ratio experiments; and (iii) 0.5 μg myc-NL1-4$_{FL}$ and 2.5 μg

HA-CD4, 2.5 μg HA-MDGA1$_{FL}$, or 3.6 μg HA-MDGA2$_{FL}$ for high ratio experiments. HA-MDGA1$_{FL}$ and HA-MDGA2$_{FL}$ plasmid DNA amounts were adjusted to achieve similar surface protein levels. One day post-transfection, COS-7 cells were seeded onto 14 day in vitro (DIV) hippocampal cultures. After 20-24 hr the co-cultures were fixed in 4% paraformaldehyde (PFA) and 4% sucrose in PBS (pH 7.4) for 12 min at room temperature and incubated with blocking solution (3% bovine serum albumin, 5% normal goat serum in PBS) for 30 min at 310K. Surface NLs and MDGAs were labeled by incubating with anti-myc and anti-HA antibodies, respectively, for 1 hr at 310K. The cells were permeabilized with 0.2% Triton X-100 in PBS, blocked for 30 min at 310K and incubated with anti-synapsin1 and anti-tau antibodies overnight at 277K. Secondary antibodies were applied for 30 min at 310K and the coverslips were mounted onto glass slides with elvanol (Tris-HCl, glycerol, and polyvinyl alcohol with 2% 1,4-diazabi-cyclo[2,2,2]octane).

The following primary antibodies were used: rabbit polyclonal anti-c-*myc* (1:1000, Sigma), mouse monoclonal anti-HA (1:1000, IgG2b, clone 12CA5, Roche), mouse monoclonal anti-synapsin1 (1:8000, IgG1, clone 46.1, Synaptic Systems), mouse monoclonal anti-tau1 (1:4000, IgG2a, clone PC1C6, Millipore). The following secondary antibodies were used: goat AMCA-conjugated anti-rabbit (1:400, Jackson ImmunoResearch), goat Alexa Fluor 488-conjugated anti-mouse IgG2b (1:1000, Thermo Fisher Scientific), goat Alexa Fluor 568-conjugated anti-mouse IgG1 (1:1000, Thermo Fisher Scientific), goat Alexa Fluor 647-conjugated anti-mouse IgG2a (1:1000, Thermo Fisher Scientific).

Image acquisition and analysis

Fluorescence microscopy was performed using a Zeiss Axioplan2 microscope. All images were acquired with a 63x oil immersion objective (NA 1.4) using MetaMorph imaging software (Molecular Devices). COS-7 cells with similar levels of both surface myc and HA were chosen for imaging and analysis for each co-culture experiment (Figure S6B). Image acquisition and analysis was performed with the experimenter blind to the experimental condition. Analysis was performed using NIH ImageJ software (Schneider et al., 2012). The total integrated intensity of punctate synapsin staining signal on a COS-7 cell was measured and normalized to the tau-positive axon contacting area. Cell surface levels of NLs and MDGAs were quantified by measuring mean intensity of myc and HA staining, respectively, on cells. Post-analysis images were adjusted for brightness and contrast across the entire image for presentation. Statistical analysis was performed using GraphPad Prism software. One-way ANOVA with post hoc Bonferroni multiple comparison test was used and statistical significance was set at $p < 0.05$. All data are presented as mean ± SEM from three independent experiments. Statistics are presented in Tables S3–S5.

Surface expression of the NL3(–A) Arg451Cys mutant

Constructs of full-length (Gln38-Val848) wild-type human NL3(–A) and mutant NL3(–A) Arg451Cys, fused N-terminally to a Myc and V5 epitope tag, were cloned into the pCAGGS vector, generating pCAGGS-myc-V5-NL3(WT) and pCAGGS-myc-V5-NL3(R451C). Both constructs used native signal sequences and were not C-terminally fused to ECFP. Low density primary hippocampal cultures were prepared from E18 rat embryos as previously described (Kaech and Banker, 2006). Neuron cultures were maintained in Neurobasal (NB) medium (Thermo Fisher Scientific) supplemented with GlutaMAX-I (Thermo Fisher Scientific), B-27 supplement (Thermo Fisher Scientific) and 100 μM APV (Abcam). Cells were transfected by nucleofection using 2 μg of plasmid DNA and were then plated on coverglasses. After 3 days in vitro (DIV) the neurons were fixed in 4% paraformaldehyde (PFA) and 4% sucrose in PBS (pH 7.4) for 12 min at room temperature and incubated with blocking solution (3% bovine serum albumin, 5% normal goat serum in PBS) for 30 min at 310K. Surface NL3 was labeled by incubating with anti-V5 antibody overnight at 277K. The cells were permeabilized with 0.2% Triton X-100 in PBS, blocked for 30 min at 310K and incubated with anti-myc antibody overnight at 277K.

The following primary antibodies were used: rabbit polyclonal anti-c-*myc* (1:1000, Sigma) and mouse monoclonal anti-V5 (1:1000, IgG2a, Thermo Fisher). The following secondary antibodies were used: goat Alexa Fluor 488-conjugated anti-rabbit (1:1000, Thermo Fisher Scientific) and goat Alexa Fluor 647-conjugated anti-mouse IgG2a (1:1000, Thermo Fisher Scientific).

Fluorescence microscopy was performed using a Zeiss Axioplan2 microscope. All images were acquired using MetaMorph imaging software (Molecular Devices) and with a 10x air objective to capture the entire neuron in one field of view. The neurons were visualized using the 488 channel for myc and 647 channel for V5.

Pulldown and mass spectrometry

Affinity chromatography was performed as previously described (Savas et al., 2014). Briefly, for each MDGA-Fc bait protein, five P21 rat brains were homogenized in homogenization buffer (4 mM HEPES, 0.32 M sucrose) with protease inhibitors using a glass Dounce homogenizer. Homogenates were centrifuged at 1,000 g for 15 min at 277 K. Supernatants were centrifuged again at 1000 g for 15 min. The resulting supernatants were then centrifuged at 10,000 g for 20 min. The pellet P2, containing crude synaptosomes, was resuspended in homogenization buffer and centrifuged at 10,000 g for 20 min, yielding pellet P2′ that contained washed crude synaptosomes. Pellet P2′ was extracted in 20 mM Tris pH 8.0, 0.1 mM $CaCl_2$ and 1% (w/v) Triton X-100 for 2 hr at 277 K. Extracts were centrifuged at 10,000 g for 30 min and the supernatants were diluted 1:1 with extraction buffer. Protein A beads (Pierce, 250 μL slurry) bound to 100 μg human Fc control protein or MDGA1-, MDGA1ΔIg1-3, or MDGA2-Fc proteins were added and rotated O/N at 277 K. Beads were packed into Poly-Prep chromatography columns (BioRad) and washed with 50 mL of high-salt wash buffer (50 mM HEPES pH 7.4, 300 mM NaCl, 0.1 mM $CaCl_2$, 5% glycerol and protease inhibitors), followed by a wash with 10 mL low-salt wash buffer (50 mM HEPES pH 7.4, 150 mM NaCl, 0.1 mM $CaCl_2$, 5% glycerol and protease inhibitors). Bound proteins were eluted from the beads by incubation with Pierce elution buffer and TCA precipitated overnight. For the MS analysis, we required

each protein to have two peptide matches and each peptide to have at least 1 tryptic terminus and an overall protein false discovery rate (FDR) < 1.2% for each dataset. Proteins shown in Figure S5B and Table S2 are the complete set of proteins found in both MDGA1- or MDGA2-Fc purifications after removing background proteins identified in Fc negative control purifications. Only proteins identified with two or more spectral counts were included in the analysis.

DATA AND SOFTWARE AVAILABILITY

The accession number for the crystal structure of human NL1(–A+B)$_{ECTO}$ reported in this paper is PDB: 5OJK. The accession number for the crystal structure of chicken MDGA1$_{ECTO}$ reported in this paper is PDB: 5OJ2. The accession number for the crystal structure of the complex between human NL1(–A–B)$_{ECTO}$ and chicken MDGA1$_{ECTO}$ reported in this paper is PDB: 5OJ6.

Neuron, Volume *95*

Supplemental Information

Structural Mechanism for Modulation of Synaptic Neuroligin-Neurexin Signaling by MDGA Proteins

Jonathan Elegheert, Vedrana Cvetkovska, Amber J. Clayton, Christina Heroven, Kristel M. Vennekens, Samuel N. Smukowski, Michael C. Regan, Wanyi Jia, Alexandra C. Smith, Hiro Furukawa, Jeffrey N. Savas, Joris de Wit, Jo Begbie, Ann Marie Craig, and A. Radu Aricescu

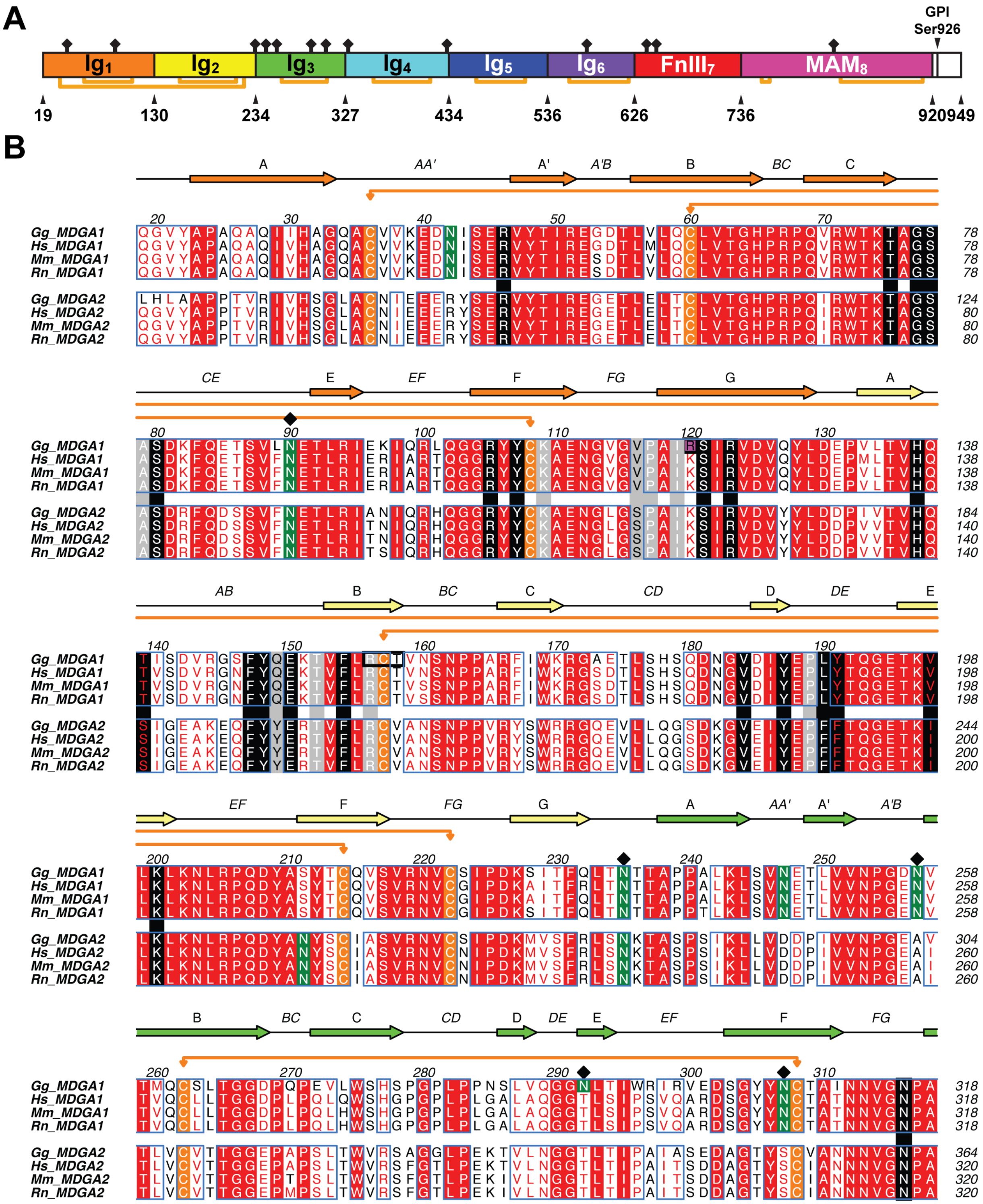
A
GPI
Ser926
Ig1
Ig2
Ig3
Ig4
Ig5
Ig6
FnIII7
MAM8
19
130
234
327
434
536
626
736
920
949
B
Gg_MDGA1
Hs_MDGA1
Mm_MDGA1
Rn_MDGA1
Gg_MDGA2
Hs_MDGA2
Mm_MDGA2
Rn_MDGA2

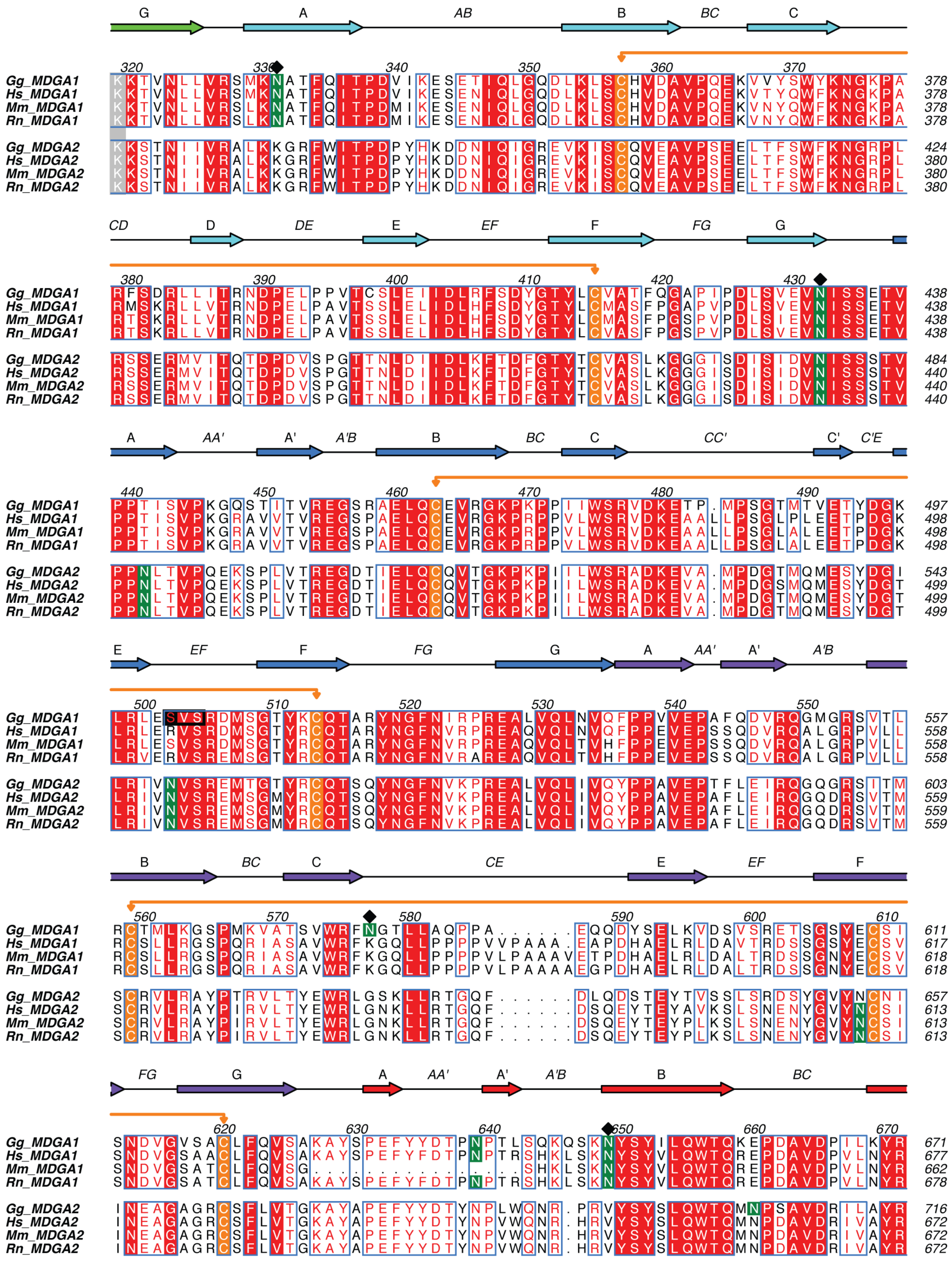
Gg_MDGA1
Hs_MDGA1
Mm_MDGA1
Rn_MDGA1
Gg_MDGA2
Hs_MDGA2
Mm_MDGA2
Rn_MDGA2

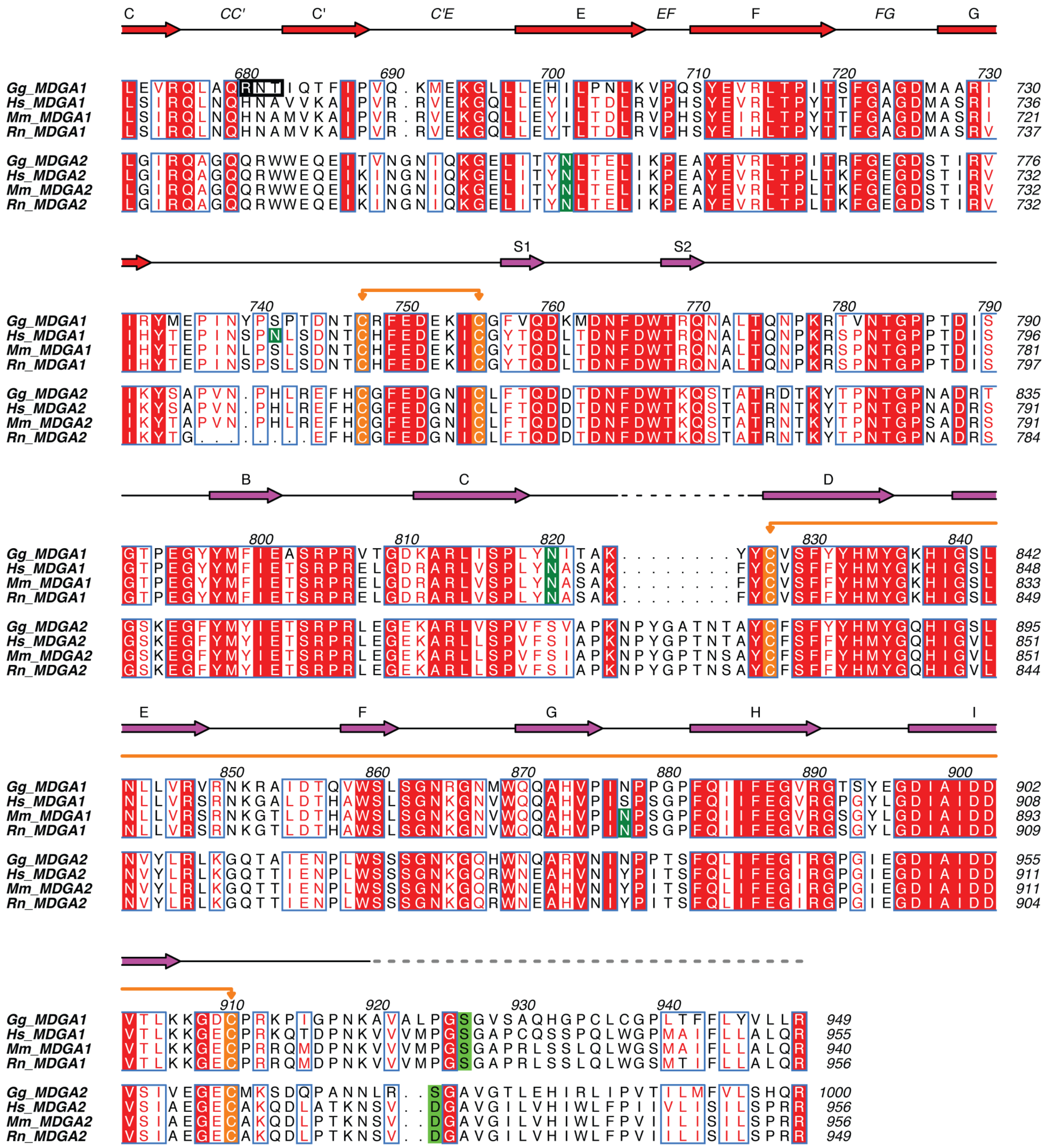

Figure S1. Sequence Alignment of the MDGA1 and -2 Ectodomains. Related to Figure 1 and Figure 2.

(A) Schematic representation of the chicken MDGA1 domain structure. Black diamonds denote predicted N-linked glycosylation sites.

(B) Amino acid sequences are taken from the UniProt database (entry code); *Gg*_MDGA1 (Q0WYX8), *Hs*_MDGA1 (Q8NFP4), *Mm*_MDGA1 (Q0PMG2), *Rn*_MDGA1 (P85171), *Gg*_MDGA2 (F1NIA0), *Hs*_MDGA2 (Q7Z553), *Mm*_MDGA2 (P60755), *Rn*_MDGA2

(P60756). *Gg*; *Gallus gallus*, *Hs*; *Homo sapiens*, *Mm*; *Mus Musculus*, *Rn*; *Rattus norvegicus*. Secondary structure elements for *Gg*_MDGA1 are annotated above the alignment. MDGA1 cysteine residues participating in disulfide bonds are shaded in orange and connected by orange lines. Putative N-linked glycosylation sites for all MDGA members are shaded in dark green. Black diamonds further highlight crystallographically confirmed N-linked MDGA1 glycosylation sites. The predicted residue positions where amide linking of the GPI anchor occurs (Ser932 in *Hs*_MDGA1 and Asp931 in *Hs*_MDGA2) are shaded in green. Positions where N-linked glycan consensus sequences (Asn-X-Ser/Thr) were inserted at cMDGA1$_{ECTO}$ crystallographic homophilic interfaces (Arg156Asn in Ig$_2$, Ser502Asn in Ig$_5$, and Arg680Asn in FnIII$_7$) are boxed in black. The Arg120Lys (R120K) mutation, introduced to bring the chicken MDGA1 sequence in line with the human, rat and mouse MDGA1-2 sequences at that position, is shaded in magenta and boxed in black. MDGA Ig$_1$, Ig$_2$, and Ig$_3$ residues in the "core" and at the "rim" of the NL–MDGA interface are highlighted in black and grey vertical rectangles, respectively. Ig$_1$ is an I-set Ig superfamily (IgSF) domain, however it lacks strand C' and D (AA'BCEFG). Ig$_2$ is a canonical C1-set IgSF domain (ABCDEFG). Ig$_3$ is an I-set IgSF domain, however it lacks strand C' (AA'BCDEFG). Ig$_4$ is a canonical C1-set IgSF domain (ABCDEFG). Ig$_5$ is an I-set IgSF domain, however it lacks strand D (AA'BCC'EFG). Ig$_6$ is an I-set IgSF domain, however it lacks strand C' and D (AA'BCEFG), similarly to Ig$_1$. Additionally, there is no canonical disulfide bond between strand B (Cys559) and strand F (Cys609) in Ig$_6$. Instead, Cys559 pairs with Cys620 on strand G. Finally, FnIII$_7$ is C2-set, and strand A is divided into strand A and A', separated by a long loop (AA'BCC'EFG).

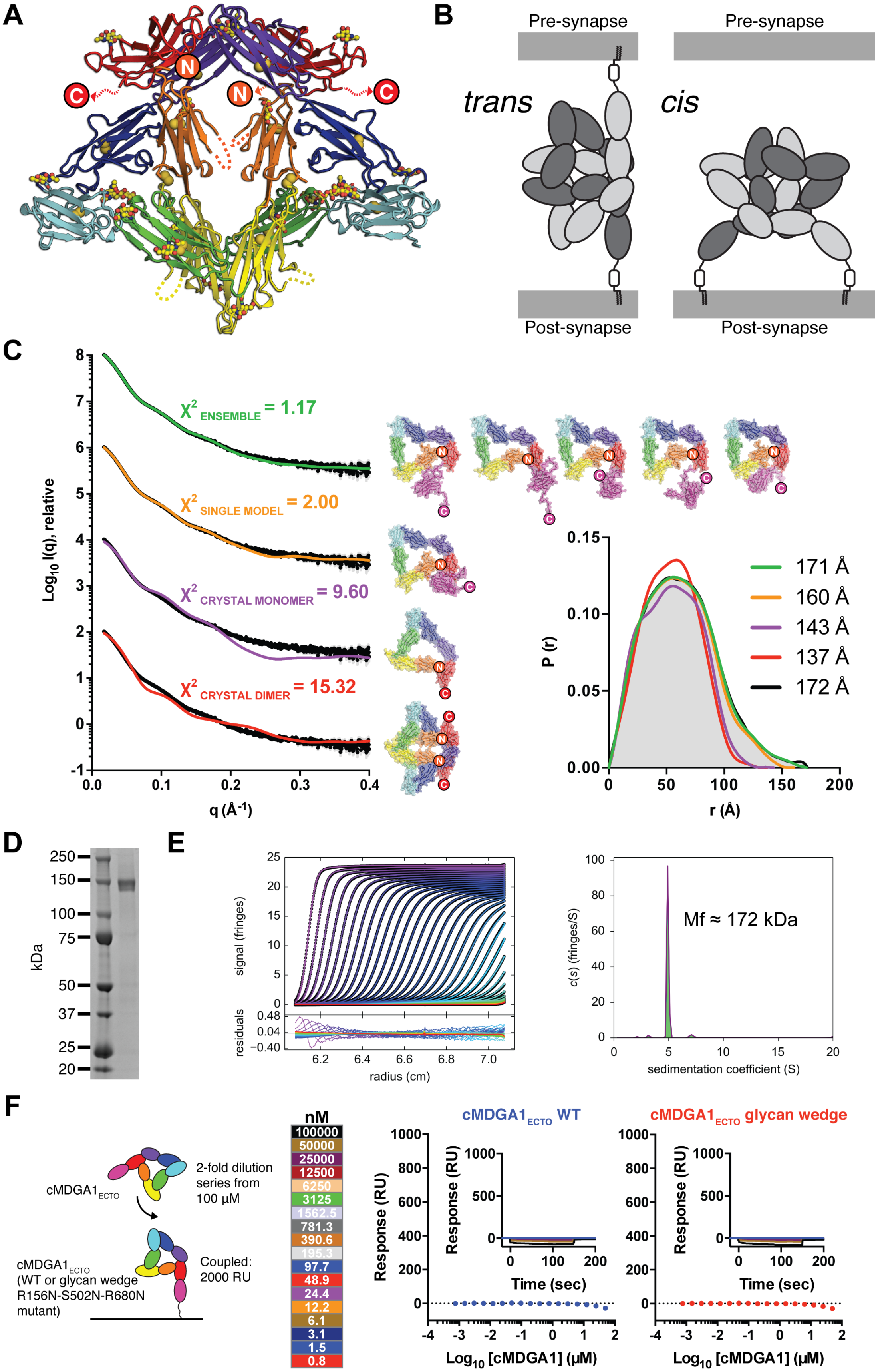
A
N
C
B
Pre-synapse
trans
cis
Post-synapse
C
χ2 ENSEMBLE = 1.17
χ2 SINGLE MODEL = 2.00
χ2 CRYSTAL MONOMER = 9.60
χ2 CRYSTAL DIMER = 15.32
Log10 I(q), relative
q (Å-1)
P (r)
r (Å)
171 Å
160 Å
143 Å
137 Å
172 Å
D
kDa
250
150
100
75
50
37
25
20
E
signal (fringes)
residuals
radius (cm)
c(s) (fringes/S)
Mf ≈ 172 kDa
sedimentation coefficient (S)
F
cMDGA1ECTO
2-fold dilution series from 100 µM
cMDGA1ECTO (WT or glycan wedge R156N-S502N-R680N mutant)
Coupled: 2000 RU
nM
100000
50000
25000
12500
6250
3125
1562.5
781.3
390.6
195.3
97.7
48.9
24.4
12.2
6.1
3.1
1.5
0.8
cMDGA1ECTO WT
cMDGA1ECTO glycan wedge
Response (RU)
Time (sec)
Log10 [cMDGA1] (µM)

Figure S2. MDGA1 is Monomeric in Solution. Related to Figure 1.

(A) Cartoon representation of the intertwined crystallographic cMDGA1$_{ECTO}$ dimer.

(B) Schematic representation of the potential MDGA1 homophilic *trans*-dimer or *cis*-dimer, GPI-anchored to the pre- and/or postsynaptic membranes.

(C) Solution structure of cMDGA1$_{ECTO}$. Experimental scattering curves (black) and calculated scattering patterns (colored) are shown to a maximal momentum transfer of q = 0.40 Å^{-1}. Individual data:fit pairs are displaced along an arbitrary y axis to allow for better visualization. Bottom curve: cMDGA1 crystallographic dimer (red). Second curve from bottom: extracted cMDGA1$_{ECTO}$ crystallographic monomer (purple). Second curve from top: best single monomeric model (orange). Top curve: best five-membered minimal ensemble (green). Atomic models, corresponding to the individual curves, are shown in ribbon and surface representation. The inset shows the experimental (black line and grey shade) and calculated (colored) pairwise distance distribution (P(r)) functions and derived maximum intra-particle distance (D_{MAX}) values.

(D) SDS-PAGE of glycosylated human MDGA1$_{ECTO}$ (hMDGA1$_{ECTO}$), showing an apparent molecular weight of ~150 kDa.

(E) SV-AUC data for hMDGA1$_{ECTO}$ at 60 µM. The sedimentation coefficient is ~5.1 S, and there is no indication of oligomer formation. The derived molar mass is consistent with a glycosylated, monomeric hMDGA1$_{ECTO}$ molecule.

(F) Lack of detectable cMDGA1$_{ECTO}$ self-interaction. Schematic representation of the structure-guided cMDGA1$_{ECTO}$ glycan wedge mutant (cMDGA1$_{ECTO}$-GW; combined Arg156Asn, Ser502Asn and Arg680Asn mutations); these mutations were chosen to disrupt crystallographically observed homophilic interfaces. Binding isotherms and sensorgrams for the (self-)interaction of wild-type cMDGA1$_{ECTO}$ and cMDGA1$_{ECTO}$-GW with wild-type cMDGA1$_{ECTO}$ are shown**.** cMDGA1$_{ECTO}$-GW serves as negative control for the detection of a potential cMDGA1$_{ECTO}$–cMDGA1$_{ECTO}$ self-interaction.

A

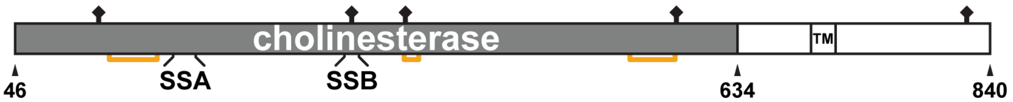

B

NL1 A1	VKRISKECARKPGKKICRKG
NL1 A2	GPLTKKQTDDLGDNDGAEDE
NL1 A1A2	VKRISKECARKPGKKICRKGGPLTKKQTDDLGDNDGAEDE
NL2 A	GPLTKKRDEATLNPPDT
NL3 A1	VKRISKECARKPNKKICRKG
NL3 A2	GSGAKKQGEDLADNDGDEDE
NL3 A1A2	VKRISKECARKPNKKICRKGGSGAKKQGEDLADNDGDEDE

C

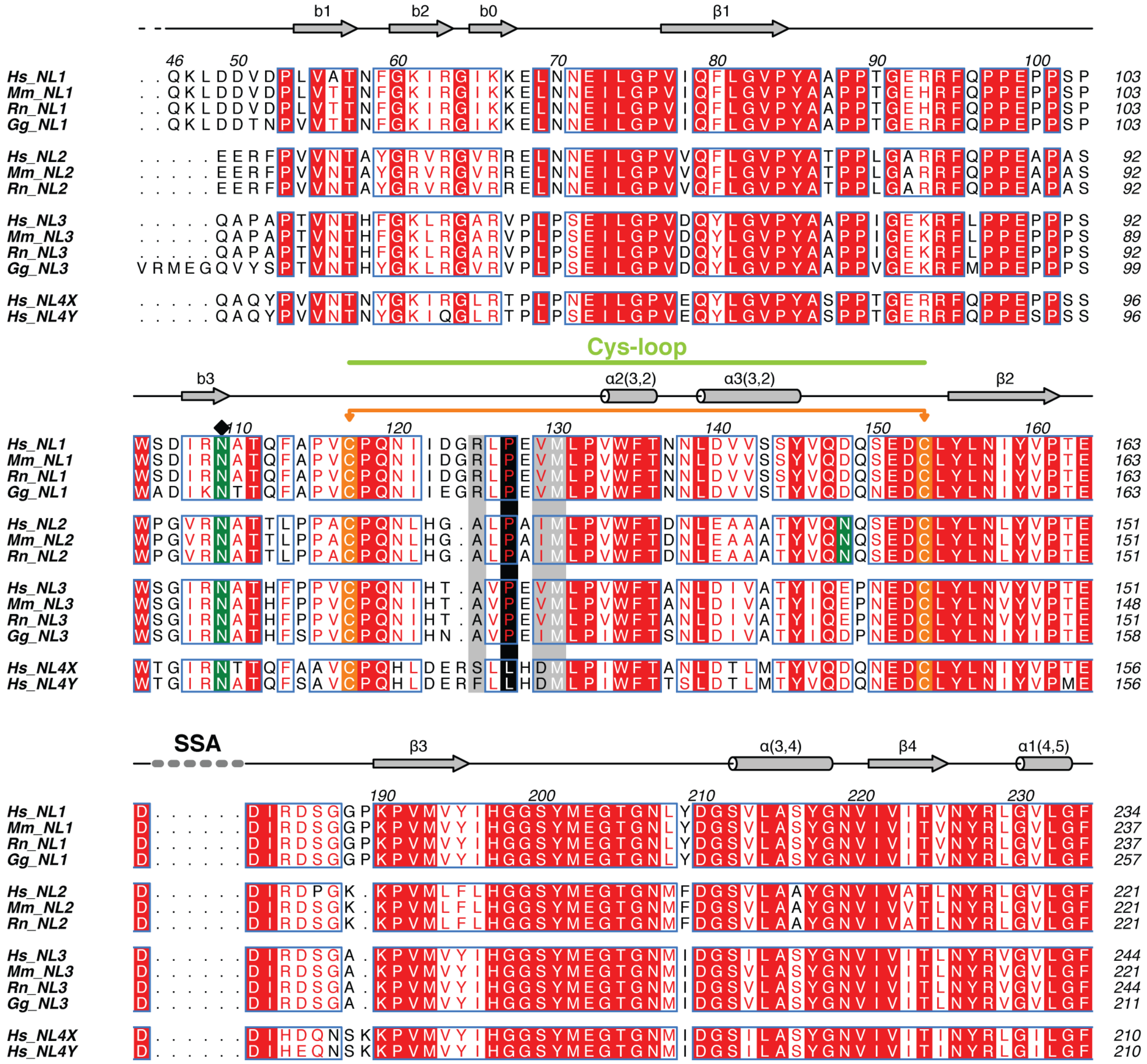

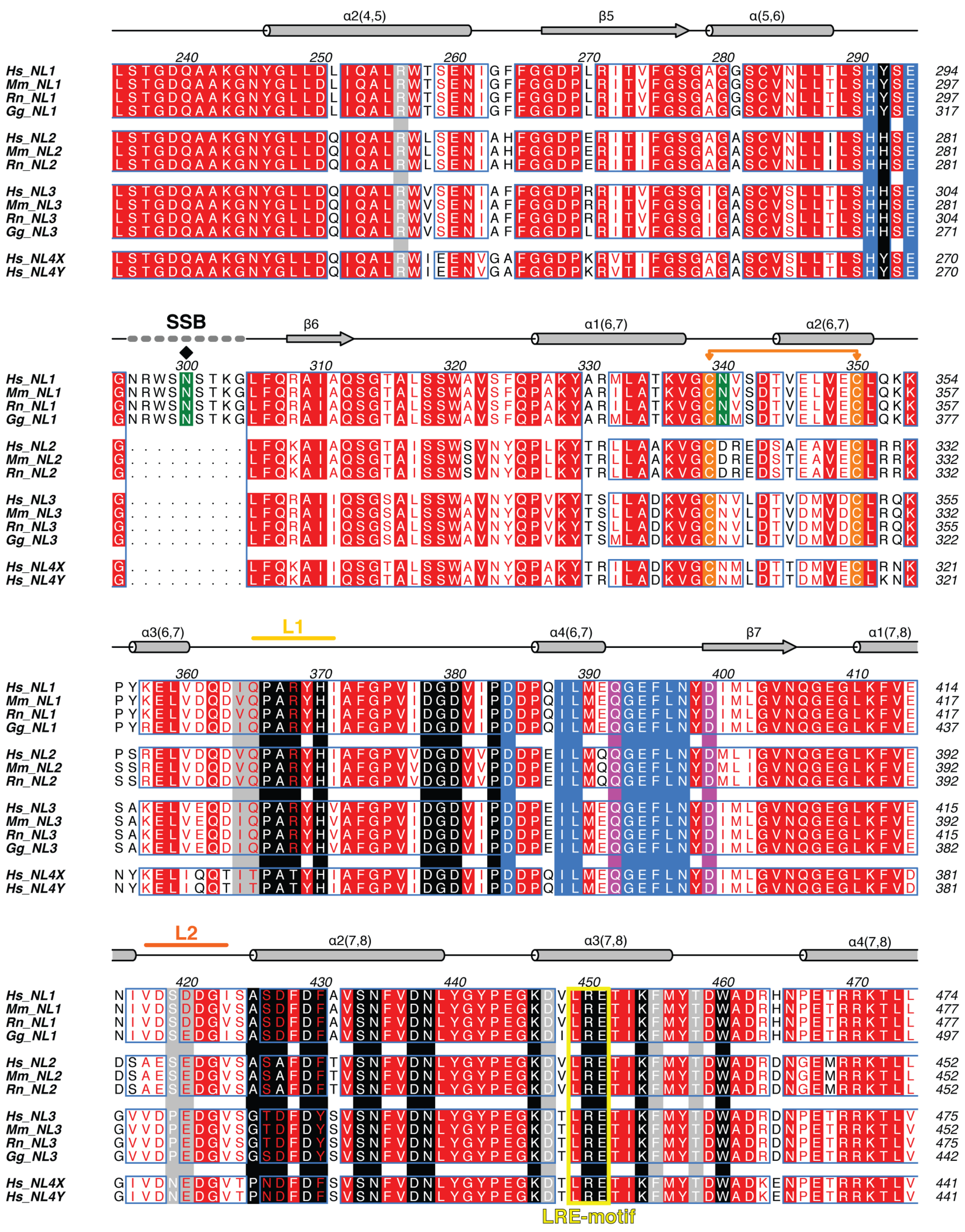
α2(4,5)
β5
α(5,6)
SSB
β6
α1(6,7)
α2(6,7)
α3(6,7)
L1
α4(6,7)
β7
α1(7,8)
L2
α2(7,8)
α3(7,8)
α4(7,8)
Hs_NL1
Mm_NL1
Rn_NL1
Gg_NL1
Hs_NL2
Mm_NL2
Rn_NL2
Hs_NL3
Mm_NL3
Rn_NL3
Gg_NL3
Hs_NL4X
Hs_NL4Y
LRE-motif

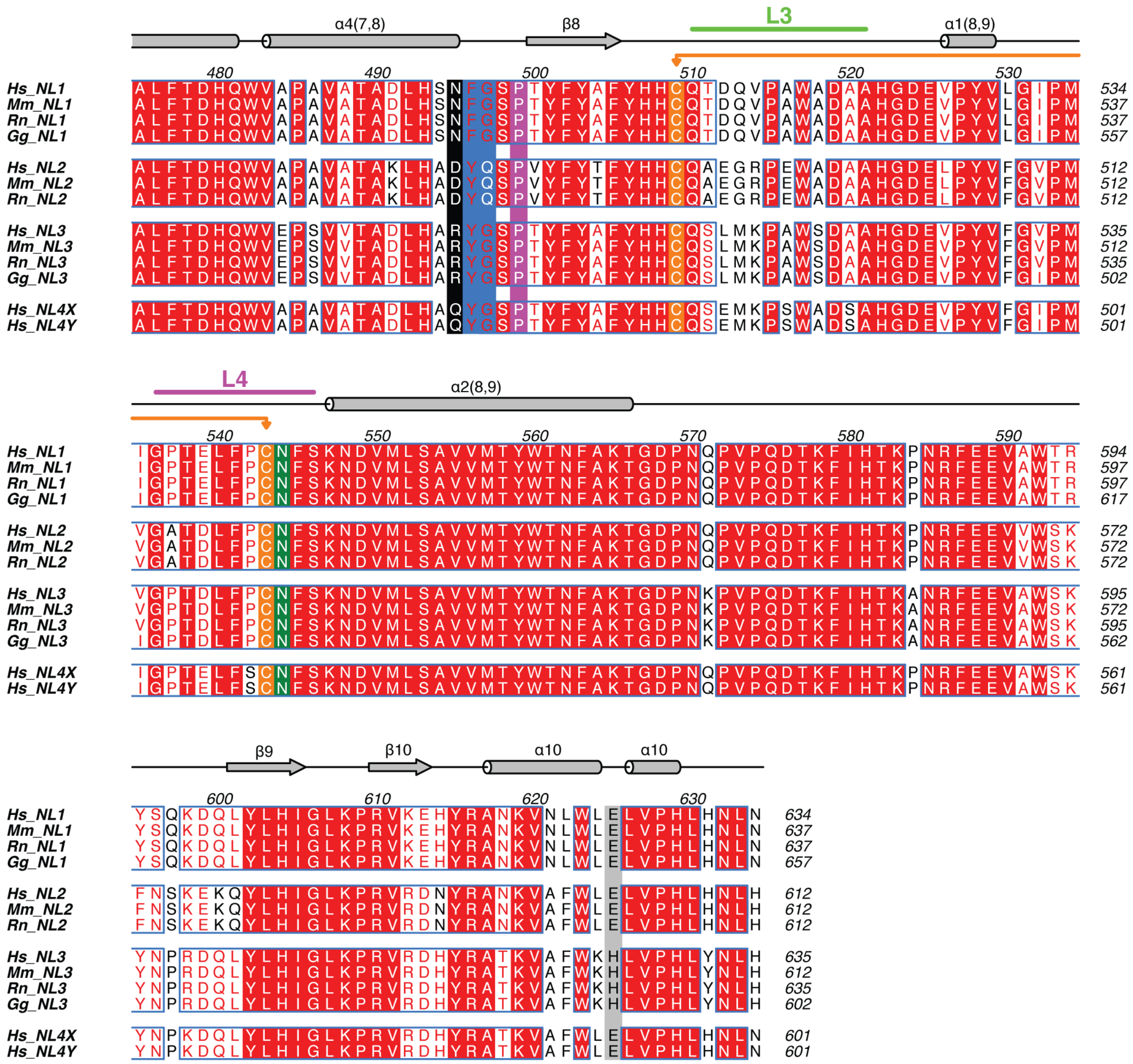

Figure S3. Sequence Alignment of the NL1, -2, -3, -4 and -5 Cholinesterase Domains. Related to Figure 2.

(A) Schematic representation of the human NL1 domain structure. SSA: spliced sequence A, SSB: spliced sequence B, TM: trans-membrane.

(B) Sequences of the different possible NL1, NL2 and NL3 spliced sequences A.

(C) Amino acid sequences are taken from the UniProt database (entry code); *Hs*_NL1 (Q8N2Q7), *Mm*_NL1 (Q99K10), *Rn*_NL1 (Q62765), *Gg*_NL1 (D2X2H3), *Hs*_NL2 (Q8NFZ4), *Mm*_NL2 (Q69ZK9), *Rn*_NL2 (Q62888), *Hs*_NL3 (Q9NZ94), *Mm*_NL3 (Q8BYM5), *Rn*_NL3 (Q62889), *Gg*_NL3 (D3WGL3), *Hs*_NL4X (Q8N0W4), *Hs*_NL4Y (*Hs*_NL5: Q8NFZ3). *Hs*; *Homo sapiens*, *Mm*; *Mus Musculus*, *Rn*; *Rattus norvegicus*, *Gg*; *Gallus gallus*. Secondary structure elements for

*Hs*_NL1 are annotated above the alignment. *Hs*_NL1 cysteine residues participating in disulfide bonds are shaded in orange and connected by orange lines. Putative N-linked glycosylation sites for all NL members are shaded in dark green (black diamonds further highlight crystallographically confirmed N-linked NL1 glycosylation sites). NL residues unique to the "core" and "rim" of the NL–MDGA interface are highlighted in black and grey vertical rectangles, respectively. NL residues unique to the NL–NRX interface are highlighted in pink vertical rectangles. NL residues that are shared between the NL–MDGA and NL–NRX interfaces are highlighted in blue vertical rectangles. The Leu-Arg-Glu (LRE) motif, conserved in all NLs and located in the α3(7,8) helix, is boxed in yellow. The positions of the Cys-loop and of loops L1, L2, L3 and L4 are indicated.

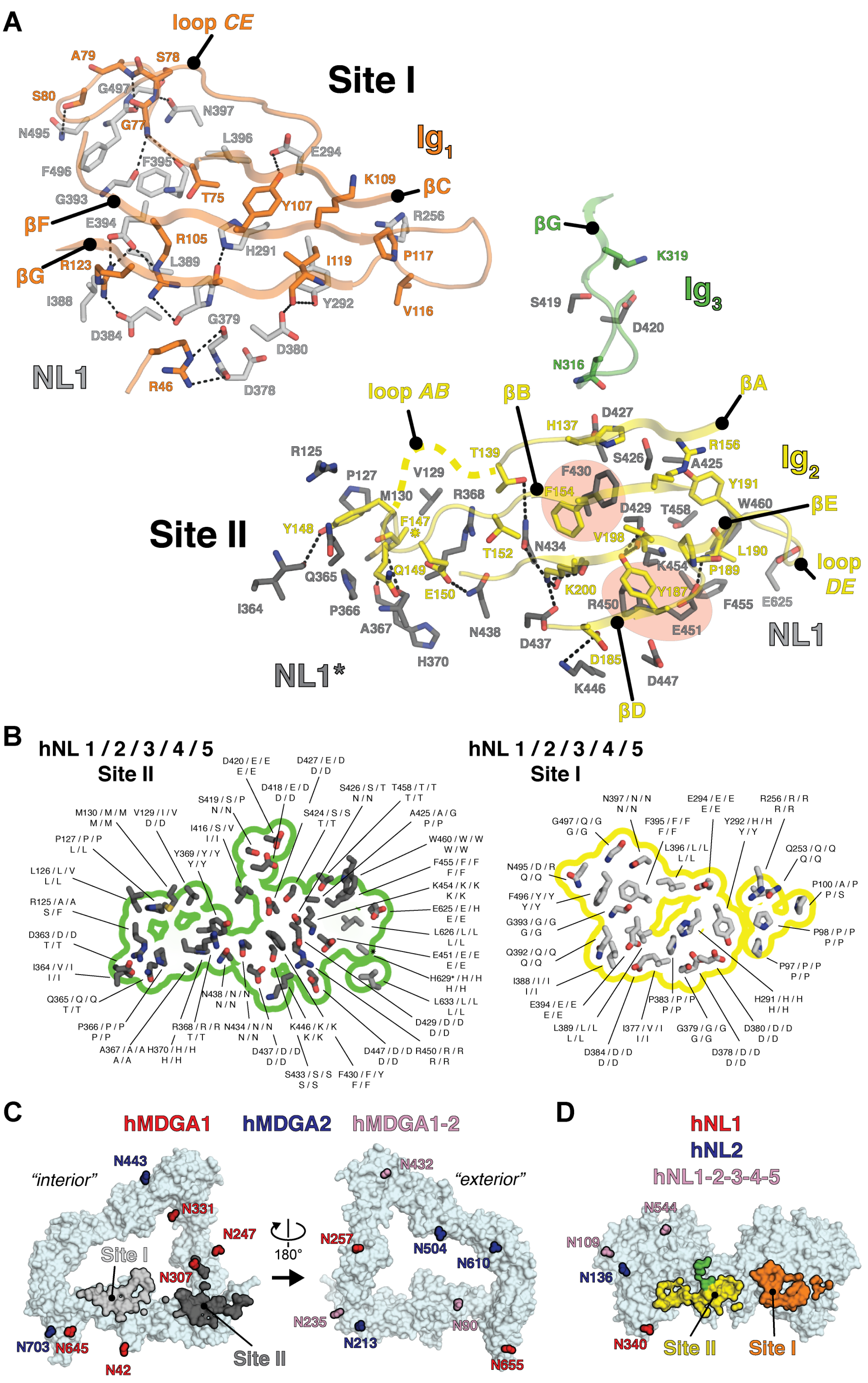

A
loop CE
Site I
Ig1
βC
βF
βG
NL1
A79
S78
S80
G497
N397
N495
G77
L396
E294
F395
F496
G393
T75
Y107
K109
E394
R256
R105
H291
R123
L389
I119
P117
I388
Y292
V116
D384
G379
D380
R46
D378
βG
K319
Ig3
S419
D420
N316
loop AB
βB
βA
H137
D427
R125
T139
R156
Ig2
S426
A425
P127
V129
F430
Y191
M130
R368
F154
D429
T458
W460
βE
Site II
Y148
F147
N434
V198
L190
loop DE
Q149
K454
P189
Q365
E150
T152
K200
Y187
I364
R450
F455
E625
P366
A367
N438
E451
NL1
H370
D437
D185
NL1*
K446
D447
βD
B
hNL 1 / 2 / 3 / 4 / 5
Site II
hNL 1 / 2 / 3 / 4 / 5
Site I
C
hMDGA1
hMDGA2
hMDGA1-2
"interior"
"exterior"
N443
N432
N331
N247
N257
N504
N610
Site I
180°
N307
N235
N213
N90
N703
N645
Site II
N655
N42
D
hNL1
hNL2
hNL1-2-3-4-5
N544
N109
N136
N340
Site II
Site I

Figure S4. Details and Conservation of the NL–MDGA Site I and Site II Interfaces. Mapping of Human MDGA1 and MDGA2 Glycosylation Sites. Related to Figure 3.

(A) Atomic details of the NL1–MDGA1 Site I and Site II interfaces. Putative hydrogen bonds and hydrophilic interactions are indicated with black dashed lines. Star symbols (*) indicate residues for which side chain electron density was not clearly discernable. The $Phe_{NL1}430$-$Phe_{MDGA1}154$ π-π sandwich stacking interaction, as well as $Arg_{NL1}450$ and $Glu_{NL1}451$ of the NL1 LRE motif, are highlighted with shaded ovals.

(B) View of the NL1 interaction interface. Site I and Site II interfaces are outlined by yellow and green lines, respectively. Per residue position, equivalent residues in human NL1, -2, -3, -4(X) and -5(=4(Y)) are annotated to highlight overall sequence conservation of the interaction interfaces. Star symbols (*) indicate residues for which side chain electron density was not clearly discernable.

(C) N-linked glycosylation sites common to human MDGA1 and -2 (pink), or unique to human MDGA1 (red) or human MDGA2 (blue) are annotated onto the $cMDGA1_{ECTO}$ structure. Site I and Site II interfaces are shown in surface representation. The hMDGA1-specific Asn307 (N307) is proximal to the edge of the Site II interface.

(D) N-linked glycosylation sites common to human NL1-2-3-4-5 (pink), or unique to human NL1 (red) or human NL2 (blue) are annotated onto the $hNL1(–A–B)_{ECTO}$ structure. Site I and Site II interfaces are shown in surface representation as in Figure 3A.

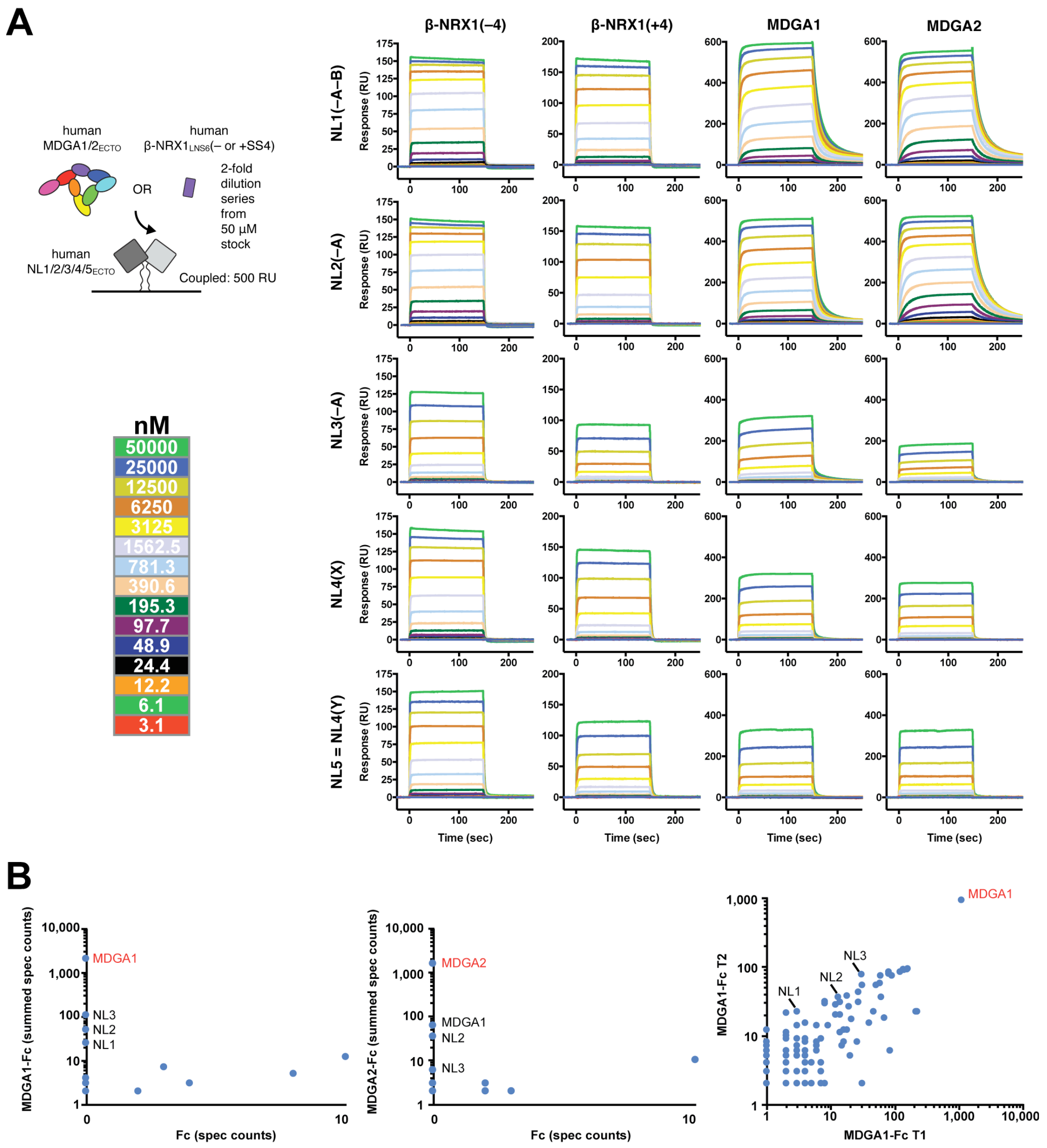

Figure S5. NL–NRX and NL–MDGA SPR Equilibrium Experiments. MDGA1 and -2 Pulldown Experiments. Related to Figure 5.

(A) Schematic representation of the SPR setup, and sensorgrams for the interaction of the human NL1, NL2, NL3, NL4 and NL5 cholinesterase domains, lacking splice inserts, with human β-NRX1$_{\mathrm{LNS6}}$ lacking and containing spliced sequence 4 (β-NRX1$_{\mathrm{LNS6}}$(–4) and β-NRX1$_{\mathrm{LNS6}}$(+4), respectively), and with human MDGA1$_{\mathrm{ECTO}}$ and MDGA2$_{\mathrm{ECTO}}$. The corresponding binding isotherms and K_D values are presented in Figure 5A.

(B) The graphs show the summed spectra count (spec count) for all surface proteins identified in two independent MDGA1-Fc or MDGA2-Fc pulldown experiments from rat brain synaptosome extracts, each compared to two negative control Fc experiments. NL1-3 are the main surface proteins specifically identified by MDGA1-Fc. MDGA2-Fc bait protein identifies NL2 and NL3 as interactors.

A

	DNA amount (μg) / plasmid ratio vs. NL1/2/3/4$_{FL}$		
	LOW	MEDIUM	HIGH
MDGA1$_{FL}$	1.1 / 2.2	1.75 / 3.5	2.5 / 5.0
MDGA2$_{FL}$	1.6 / 3.2	2.5 / 5.0	3.6 / 7.2
CD4	1.1 / 2.2	1.75 / 3.5	2.5 / 5.0
NL1/2/3/4$_{FL}$	0.5	0.5	0.5

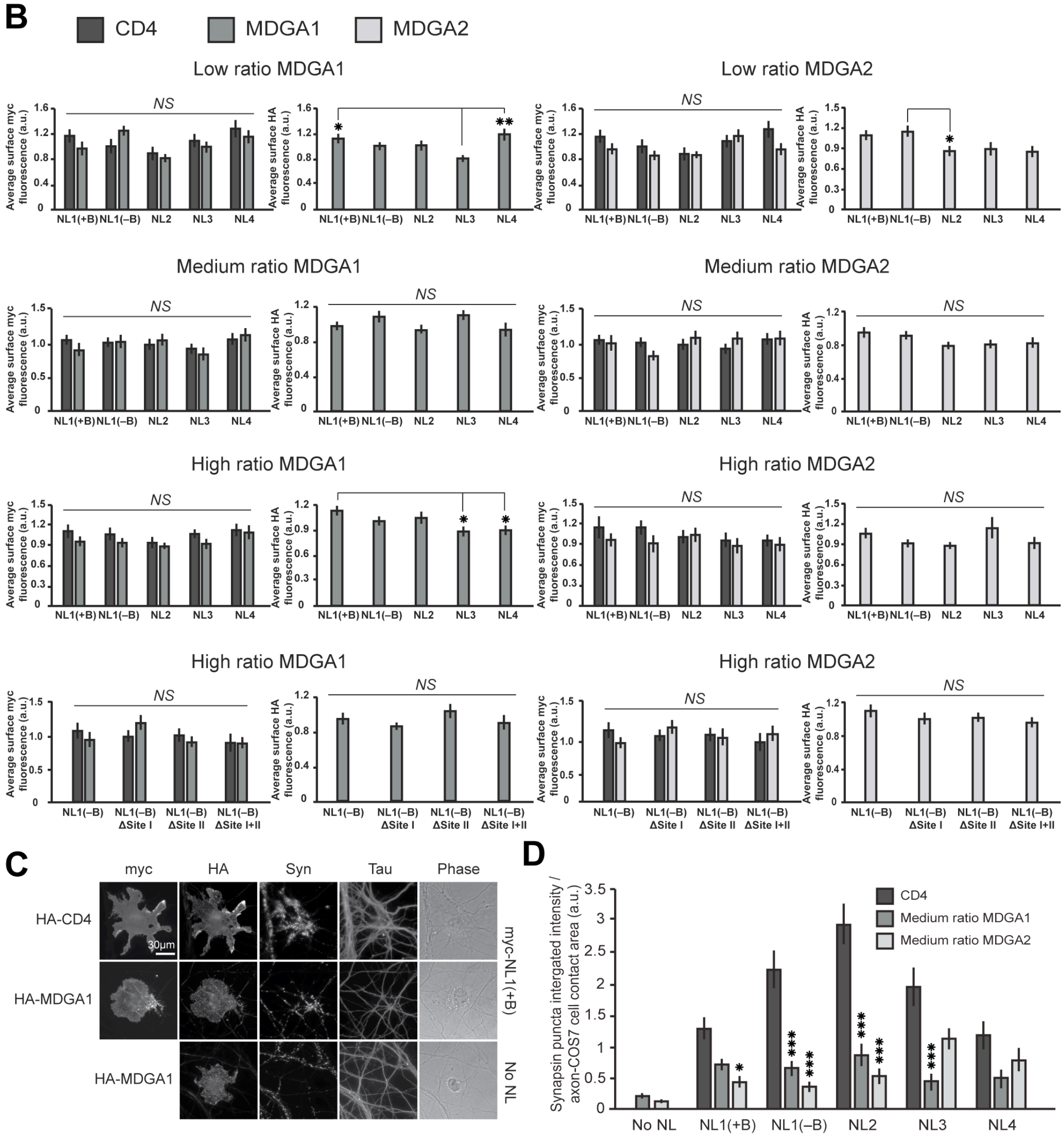

Figure S6. Comparable Levels of Surface NLs And MDGAs in the Hemi-synapse Formation Assays. Related to Figure 5, Figure 6, and Figure 8.

(A) Summary of MDGA:NL plasmid DNA ratios.

(B) COS-7 cells chosen for analysis displayed similar levels of myc-NL1-4 and HA-MDGA1-2 in each co-culture experiment. For each condition (low ratio MDGA1, medium ratio MDGA1,

high ratio MDGA1, low ratio MDGA2, medium ratio MDGA2, and high ratio MDGA2) the mean myc signal (for surface NLs) and mean HA signal (for surface MDGAs) was measured and cells that did not have similar levels of each protein were excluded from analysis. One-way ANOVA with Bonferonni *post hoc* comparison was used to determine statistical significance. Error bars represent the SEM. *; $p < 0.05$, **; $p < 0.01$.

(C) Examples are shown for all the channels imaged for myc-NL1(–A+B) co-transfected with HA-CD4 or HA-MDGA1, and for the HA-MDGA1 only (no NL) negative control. Scale bar is 30 μm.

(D) The bar graph shows the hemi-synapse formation assay data for MDGA1 and MDGA2 at medium ratio without normalization. Each NL co-transfected with CD4 shows a different baseline level of synapsin clustering, with NL2 being the most potent and NL4 being the least potent at hemi-synapse induction. Error bars represent the SEM. *; $p < 0.05$, ***; $p < 0.001$.

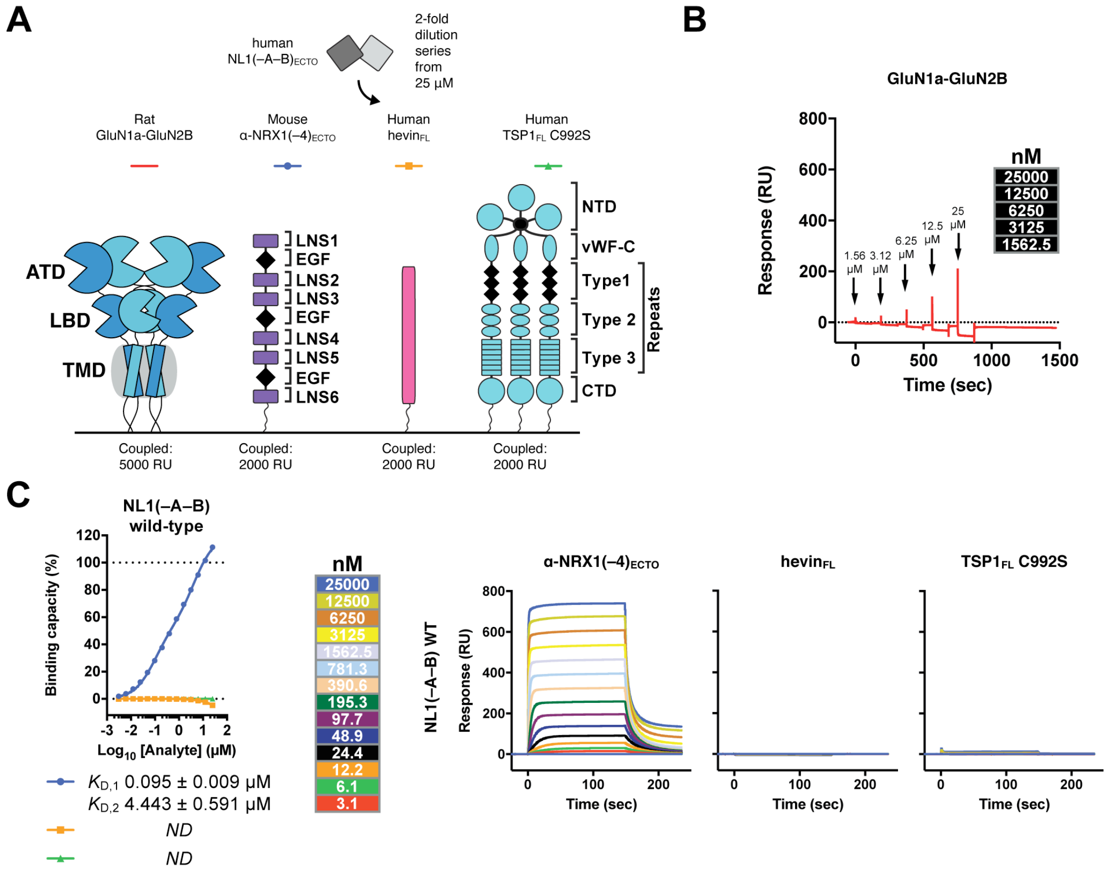

Figure S7. Assessment of Binding of NL1 with Hevin, Thrombospondin-1 and the NMDA Receptor. Related to Figure 5.

(A) Schematic representation of the SPR setup.

(B) Single-cycle kinetics (SCK) sensorgram of the interaction of NL1(–A–B)$_{\mathrm{ECTO}}$ with the GluN2a–GluN2B NMDA receptor.

(C) Binding isotherms and sensorgrams for the interaction of the human NL1(–A–B)$_{\mathrm{ECTO}}$ cholinesterase domains with mouse α-NRX1$_{\mathrm{ECTO}}$(–4), full-length (FL) human hevin (hevin$_{\mathrm{FL}}$) and human TSP1$_{\mathrm{FL}}$.

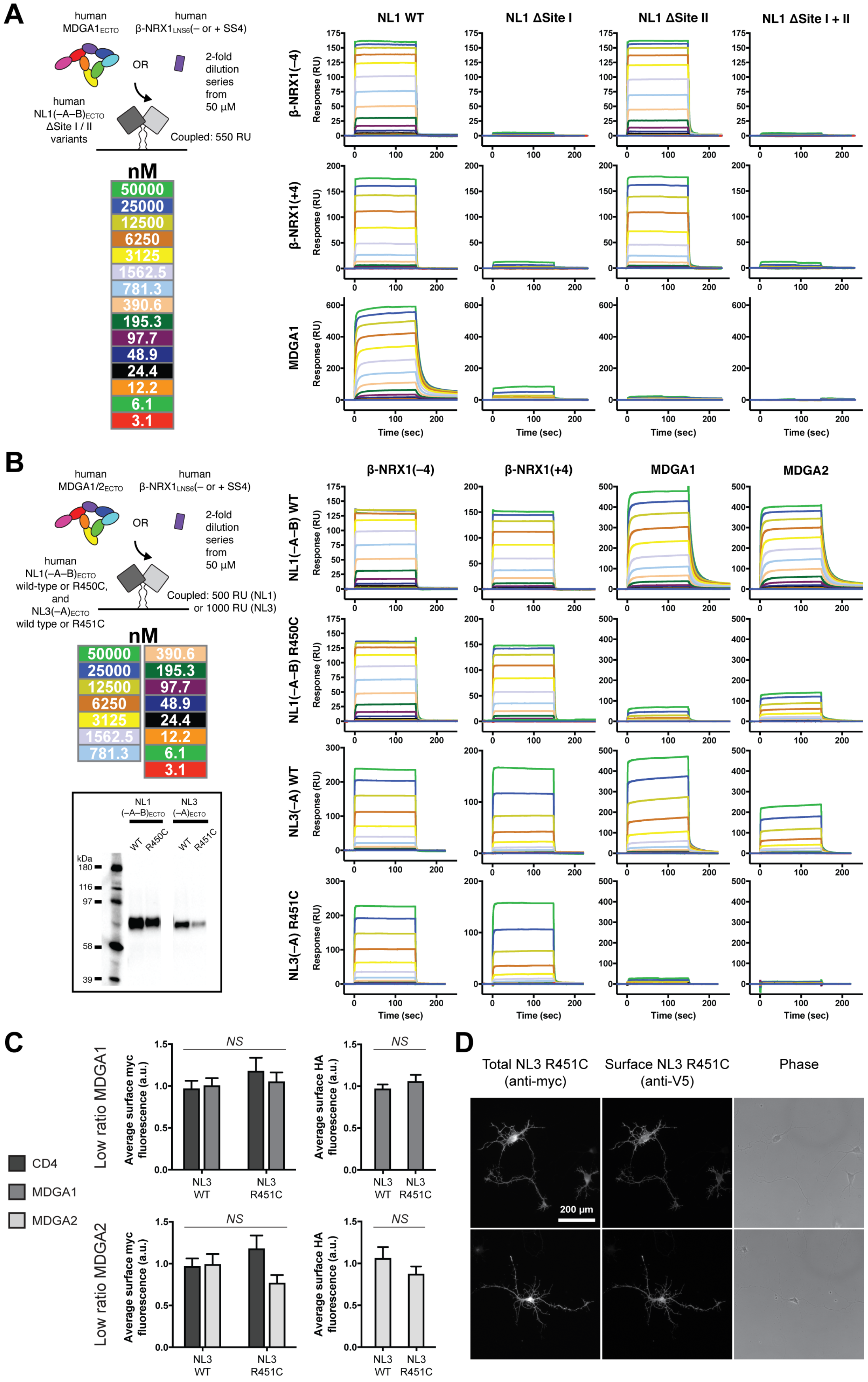
A
human MDGA1ECTO
human β-NRX1LNS6(– or + SS4)
OR
2-fold dilution series from 50 µM
human NL1(–A–B)ECTO ΔSite I / II variants
Coupled: 550 RU
nM
50000
25000
12500
6250
3125
1562.5
781.3
390.6
195.3
97.7
48.9
24.4
12.2
6.1
3.1
NL1 WT
NL1 ΔSite I
NL1 ΔSite II
NL1 ΔSite I + II
β-NRX1(–4)
β-NRX1(+4)
MDGA1
Response (RU)
Time (sec)
B
human MDGA1/2ECTO
human β-NRX1LNS6(– or + SS4)
OR
2-fold dilution series from 50 µM
human NL1(–A–B)ECTO wild-type or R450C, and NL3(–A)ECTO wild type or R451C
Coupled: 500 RU (NL1) or 1000 RU (NL3)
nM
NL1 (–A–B)ECTO
NL3 (–A)ECTO
WT
R450C
WT
R451C
kDa
180
116
97
58
39
β-NRX1(–4)
β-NRX1(+4)
MDGA1
MDGA2
NL1(–A–B) WT
NL1(–A–B) R450C
NL3(–A) WT
NL3(–A) R451C
Response (RU)
Time (sec)
C
CD4
MDGA1
MDGA2
Low ratio MDGA1
Low ratio MDGA2
Average surface myc fluorescence (a.u.)
Average surface HA fluorescence (a.u.)
NS
NL3 WT
NL3 R451C
D
Total NL3 R451C (anti-myc)
Surface NL3 R451C (anti-V5)
Phase
200 µm

Figure S8. Mutational Analysis of the NL1 Site I and Site II Interfaces. Effect of the ASD-linked NL3 Mutation Arg451Cys on the Interaction of NL3 with MDGA1 and MDGA2. Related to Figure 6 and Figure 7.

(A) Schematic representation of the SPR setup, and sensorgrams for the interaction of wild-type human NL1(–A–B)$_{ECTO}$ and the human NL1(–A–B)$_{ECTO}$ Site I (ΔSite I), Site II (ΔSite II) and Site I+II (ΔSite I+II) core interface mutants, with human β-NRX1$_{LNS6}$ lacking and containing spliced sequence 4 (β-NRX1$_{LNS6}$(–4) and β-NRX1$_{LNS6}$(+4), respectively), and with human MDGA1$_{ECTO}$. Consistent with the respective crystal structures (Figure 4A), ΔSite II abolishes MDGA1 binding, but does not affect β-NRX1 binding. The corresponding binding isotherms and K_D values are presented in Figure 6B.

(B) Schematic representation of the SPR setup, and sensorgrams for the interaction of the human NL1, NL1 Arg450Cys, NL3 and NL3 Arg451Cys cholinesterase domains, lacking splice inserts, with human β-NRX1$_{LNS6}$ lacking and containing spliced sequence 4 (β-NRX1$_{LNS6}$(–4) and β-NRX1$_{LNS6}$(+4), respectively), and with human MDGA1$_{ECTO}$ and MDGA2$_{ECTO}$. The corresponding binding isotherms and K_D values are presented in Figure 7C. The inset shows a Western blot of the NL1(–A–B)$_{ECTO}$-WT, NL1(–A–B)$_{ECTO}$-R450C, NL3(–A)$_{ECTO}$-WT and NL3(–A)$_{ECTO}$-R451C proteins secreted by HEK293T cells into the growth medium. Detection of the biotinylated Avitag was performed using streptavidin-HRP conjugate (1:50,000 dilution, Sigma Aldrich).

(C) Comparable levels of surface NL3 and MDGAs in the hemi-synapse formation assay. COS-7 cells chosen for analysis displayed similar levels of myc-NL3 and HA-MDGA1-2 in each co-culture experiment. The mean myc signal (for surface NL3s) and mean HA signal (for surface MDGAs) was measured and cells that did not have similar levels of each protein were excluded from analysis. One-way ANOVA with Bonferonni *post hoc* comparison was used to determine statistical significance. Error bars represent the SEM.

(D) Expression of full-length human Myc-V5-NL3-R451C on the cell surface of rat hippocampal neurons. Representative images of neurons immunostained for total myc-NL3 or for surface V5-NL3 are shown. Scale bar is 200 μm.

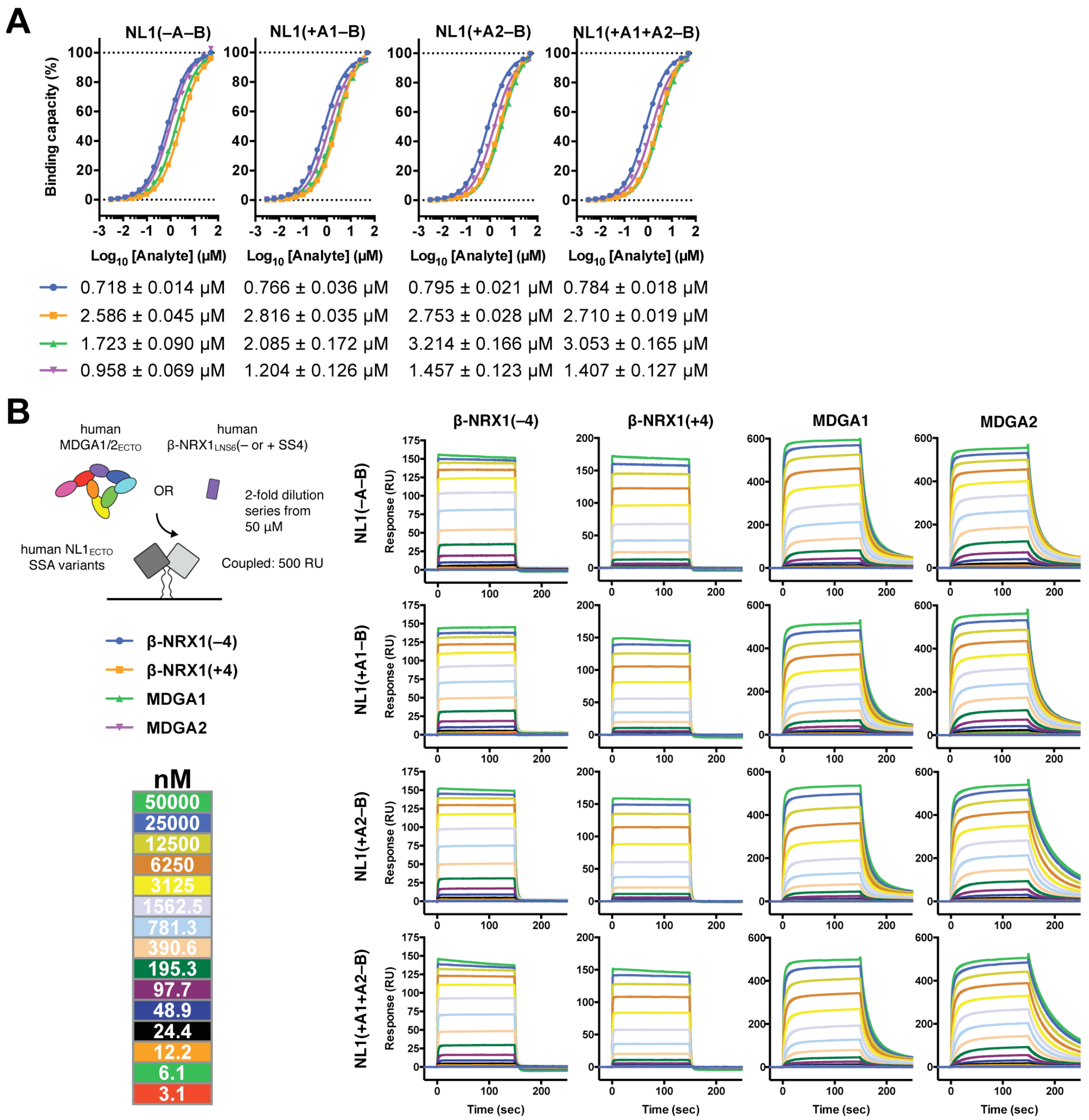

Figure S9. NL1 Spliced Sequence A (SSA) Does Not Modulate NL1–NRX or NL1–MDGA Interactions. Related to Figure 8.

(A) Binding isotherms and summary of K_D values for the interaction of the human $NL1(\pm A1\pm A2–B)_{ECTO}$ cholinesterase domains with human β-$NRX1_{LNS6}$ lacking and containing spliced sequence 4 (β-$NRX1_{LNS6}$(–4) and β-$NRX1_{LNS6}$(+4), respectively), and with human $MDGA1_{ECTO}$ and $MDGA2_{ECTO}$. Data for $NL1(–A–B)_{ECTO}$ have been repeated here from Figures 5A and S5A for clearer comparison.

(B) Schematic representation of the SPR setup and sensorgrams corresponding to (A)**.**

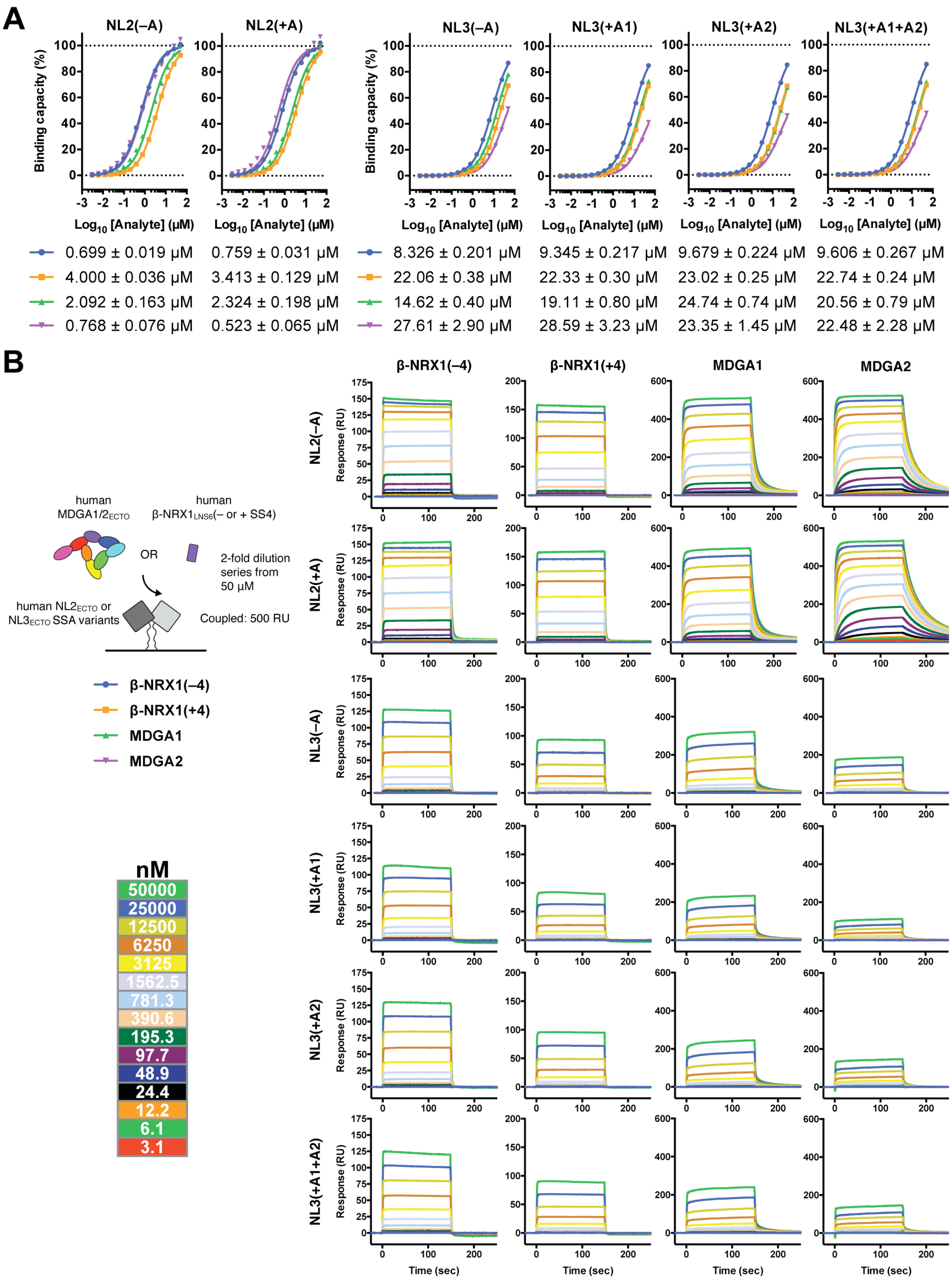

Figure S10. NL2 or NL3 Spliced Sequences A (SSA) Do Not Modulate NL2/3–NRX or NL2/3–MDGA Interactions. Related to Figure 8.

(A) Binding isotherms and summary of K_D values for the interaction of the human NL2(±A)$_{ECTO}$ or NL3(±A1±A2)$_{ECTO}$ cholinesterase domains with human β-NRX1$_{LNS6}$ lacking and containing

spliced sequence 4 (β-NRX1$_{LNS6}$(–4) and β-NRX1$_{LNS6}$(+4), respectively), and with human MDGA1$_{ECTO}$ and MDGA2$_{ECTO}$. Data for NL2(–A)$_{ECTO}$ and NL3(–A)$_{ECTO}$ have been repeated here from Figures 5A and S5A for clearer comparison.

(B) Schematic representation of the SPR setup and sensorgrams corresponding to (A)**.**

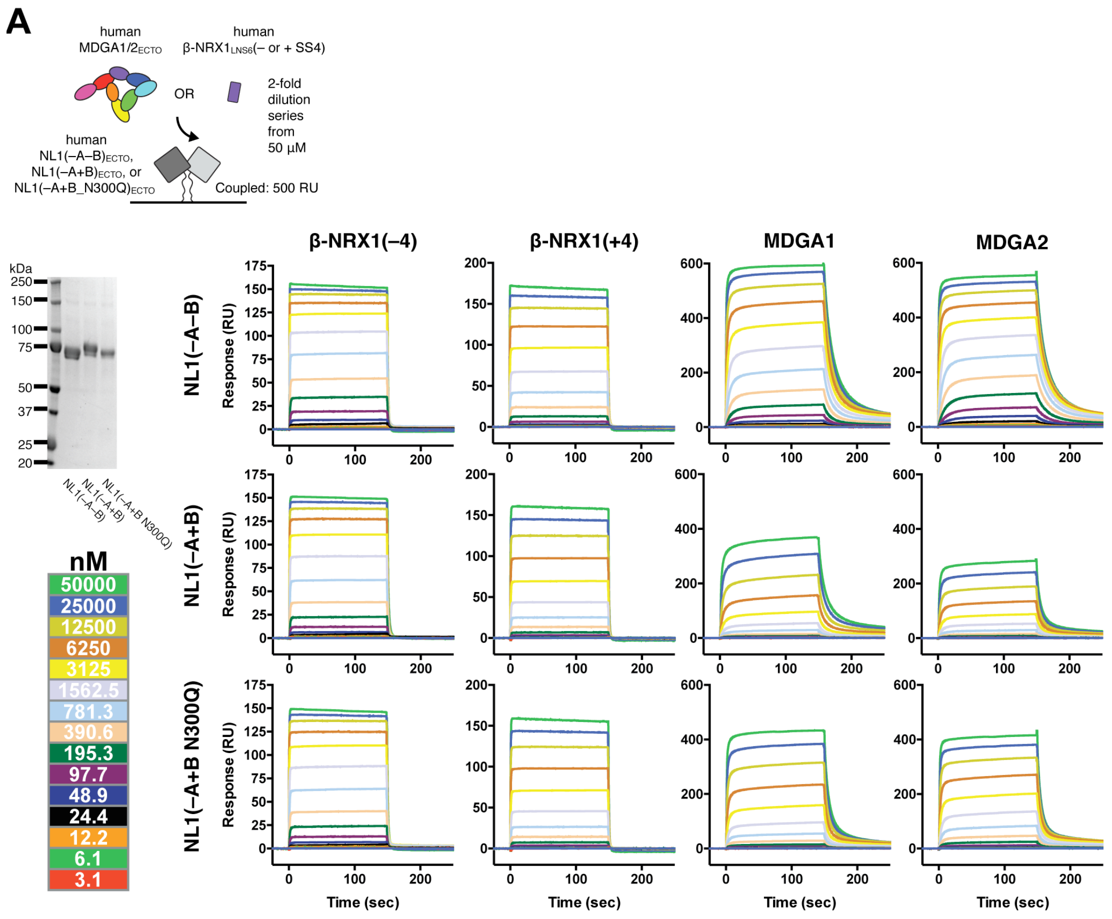

Figure S11. NL1 Spliced Sequence B (SSB) Differentially Modulates NL1–NRX and NL1–MDGA Interactions. Related to Figure 8.

(A) Schematic representation of the SPR setup, and sensorgrams for the interaction of human NL1(–A–B)$_{ECTO}$, NL1(–A+B)$_{ECTO}$ and NL1(–A+B_Asn300Gln)$_{ECTO}$ with human β-NRX1$_{LNS6}$ lacking and containing spliced sequence 4 (β-NRX1$_{LNS6}$(–4) and β-NRX1$_{LNS6}$(+4), respectively), and with human MDGA1$_{ECTO}$ and MDGA2$_{ECTO}$. The SDS-PAGE gel shows NL1(–A–B)$_{ECTO}$, NL1(–A+B)$_{ECTO}$ and NL1(–A+B_Asn300Gln)$_{ECTO}$, expressed in HEK 293T cells, indicating their relative differences in apparent molecular weight due to their different glycosylation states. Data for NL1(–A–B)$_{ECTO}$ have been repeated here from Figures 5A and S5A for clearer comparison. The corresponding binding isotherms and K_D values are presented in Figure 8C.

Table S1. Crystallographic Data Collection and Refinement Statistics. Related to Figure 1, Figure 2, and Figure 8.

	**hNL1 (–A+B) $_{ECTO}$**	**cMDGA1$_{ECTO}$ SeMet-labeled**	**hNL1(–A–B)$_{ECTO}$–cMDGA1$_{ECTO}$**
PDB code	5OJK	5OJ2	5OJ6
**DATA COLLECTION**			
Source	DLS I24	DLS I03	DLS I04-1
Wavelength λ (Å)	0.96860	0.97938 (peak)	0.92000
No. of crystals	1	1	1
Resolution (Å)	51.70-2.55 (2.62-2.55)	96.65-3.20 (3.28-3.20)	92.07-3.30 (3.39-3.30)
Space group	$P22_12_1$	$P2_12_12_1$	$P2_12_12$
Cell dimensions; a, b, c (Å)	49.57, 118.05, 214.34	103.18, 109.04, 208.77	109.42, 184.14, 96.47
Unique reflections	40826 (3077)	39616 (2874)	29576 (2080)
Multiplicity	7.5 (7.3)	11.0 (10.9)	4.5 (4.6)
Completeness (%)	97.3 (98.8)	100.0 (99.8)	98.7 (95.4)
R_{MERGE} (%)	17.9 (126.6)	14.8 (134.8)	7.5 (84.0)
R_{MEAS} (%)	19.3 (136.2)	15.5 (141.5)	9.5 (106.3)
R_{PIM} (%)	6.8 (48.4)	4.6 (42.3)	5.8 (64.1)
$CC_{1/2}$ (%)	99.6 (54.1)	99.8 (62.2)	99.9 (63.8)
CC* (%)	99.9 (83.8)	99.9 (87.6)	99.9 (88.3)
Average $I/\sigma(I)$	8.4 (1.5)	13.6 (1.8)	15.6 (1.6)
**REFINEMENT**			
Resolution (Å)	51.70-2.55 (2.61-2.55)	96.65-3.20 (3.24-3.20)	72.38-3.30 (3.40-3.30)
Reflections (WORK / FREE set)	38734 / 2005	35762 / 3851	28098 / 1474
R_{WORK} / R_{FREE} (%)	0.2360 / 0.2780 (0.3331 / 0.3658)	0.2144 / 0.2514 (0.3767 / 0.3808)	0.2269 / 0.2771 (0.3181 / 0.3838)
CC_{WORK} / CC_{FREE} in highest resolution shell (%)	0.732 / 0.629	0.579 / 0.563	0.814 / 0.676
No. of atoms (NL1 / MDGA1 / NAG-BMA-MAN / PGE / water)	8406 / 0 / 14 / 20 / 109	0 / 10788 / 299 / 0 / 0	4278 / 5381 / 151 / 0 / 0
B factors (Å^2) (NL1 / MDGA1 / NAG-BMA-MAN / PGE / water)	50.8 / - / 71.3 / 52.9 / 44.6	- / 105.2 / 146.7 / - / -	92.5 / 129.6 / 158.0 / - / -
R.m.s.d. Bonds (Å)	0.003	0.004	0.003
R.m.s.d. angles (°)	0.782	0.986	0.939
Ramachandran			
Favored (%)	94.79	92.37	92.78
Allowed (%)	5.21	7.56	7.14
Outliers (%)	0.00	0.07	0.08
Molprobity score / percentile	1.58 / 99th	1.82 / 100th	1.99 / 100th

Numbers in parentheses refer to the highest resolution shell.

R.m.s.d.: root mean square deviation from ideal geometry.

R_{PIM}: precision-indicating merging R-factor.

R_{MEAS}: multiplicity-corrected R_{SYM}.

$CC_{1/2}$, CC*: correlation coefficients between random half data sets.

CC_{WORK} / CC_{FREE}: standard and cross-validated correlations of the experimental intensities with the intensities calculated from the refined molecular model.

NAG: N-Acetyl-D-Glucosamine.

BMA: Beta-D-mannose.

MAN: Alpha-D-mannose.

PGE: Triethylene glycol.

Table S2. Mass Spectrometry Summary Data File. Related to Figure S5.

Mass spectrometry summary data file for MDGA1 (two independent experiments), MDGA2 (two independent experiments), and MDGA1ΔIg1-3 (one experiment). Column headers containing spectra counts are highlighted in bold. Protein descriptions are listed in the far-right column. Data are sorted by MDGA1-Fc spectra count in descending order.

Table S3. One-way Analysis of Variance of the Effect of Co-expression of NL with MDGA1 or MDGA2 at Low, Medium and High Plasmid Ratios. Related to Figure 5 and Figure 8.

ANOVA	F value			*p* value
MDGA1 : NL1-4 low	9.87			< 0.0001
Bonferroni post hoc comparison	Mean difference	t value	Significant at *p* < 0.05	95% confidence interval of mean difference
NL1(+B): CD4 vs. MDGA1	0.1009	0.4912	No	-0.5770 to 0.7789
NL1(–B): CD4 vs. MDGA1	0.1761	0.8487	No	-0.5085 to 0.8607
NL2: CD4 vs. MDGA1	1.526	6.791	Yes	0.7849 to 2.268
NL3: CD4 vs. MDGA1	0.9278	4.497	Yes	0.2471 to 1.608
NL4: CD4 vs. MDGA1	0.02018	0.0918	No	-0.7054 to 0.7458

ANOVA	F value			*p* value
MDGA1 : NL1-4 medium	15.18			< 0.0001
Bonferroni post hoc comparison	Mean difference	t value	Significant at *p* < 0.05	95% confidence interval of mean difference
NL1(+B): CD4 vs. MDGA1	0.5515	1.892	No	-0.4076 to 1.511
NL1(–B): CD4 vs. MDGA1	1.541	5.076	Yes	0.5423 to 2.540
NL2: CD4 vs. MDGA1	2.028	6.794	Yes	1.046 to 3.010
NL3: CD4 vs. MDGA1	1.484	4.861	Yes	0.4795 to 2.488
NL4: CD4 vs. MDGA1	0.6552	2.026	No	-0.4087 to 1.719

ANOVA	F value			*p* value
MDGA1 : NL1-4 high	31.63			< 0.0001
Bonferroni post hoc comparison	Mean difference	t value	Significant at *p* < 0.05	95% confidence interval of mean difference
NL1(+B): CD4 vs. MDGA1	0.9434	4.373	Yes	0.2338 to 1.653
NL1(–B): CD4 vs. MDGA1	2.165	10.35	Yes	1.477 to 2.853
NL2: CD4 vs. MDGA1	1.856	8.241	Yes	1.115 to 2.596
NL3: CD4 vs. MDGA1	1.457	6.674	Yes	0.7390 to 2.175
NL4: CD4 vs. MDGA1	0.4879	2.106	No	-0.2741 to 1.250

ANOVA	F value			*p* value
MDGA2 : NL1-4 low	14.83			< 0.0001
Bonferroni post hoc comparison	Mean difference	t value	Significant at *p* < 0.05	95% confidence interval of mean difference
NL1(+B): CD4 vs. MDGA2	0.7083	3.642	Yes	0.06660 to 1.350

NL1(–B): CD4 vs. MDGA2	1.273	6.726	Yes	0.6488 to 1.898
NL2: CD4 vs. MDGA2	1.653	7.840	Yes	0.9573 to 2.349
NL3: CD4 vs. MDGA2	0.4456	2.302	No	-0.1929 to 1.084
NL4: CD4 vs. MDGA2	-0.06702	0.3139	No	-0.7716 to 0.6375

ANOVA	F value			*p* value
MDGA2 : NL1-4 medium	14.08			< 0.0001
Bonferroni post hoc comparison	Mean difference	t value	Significant at $p < 0.05$	95% confidence interval of mean difference
NL1(+B): CD4 vs. MDGA2	1.031	3.334	Yes	0.01342 to 2.048
NL1(–B): CD4 vs. MDGA2	1.825	5.805	Yes	0.7903 to 2.859
NL2: CD4 vs. MDGA2	2.376	7.688	Yes	1.359 to 3.393
NL3: CD4 vs. MDGA2	0.7810	2.354	No	-0.3110 to 1.873
NL4: CD4 vs. MDGA2	0.3797	1.125	No	-0.7313 to 1.491

ANOVA	F value			*p* value
MDGA2 : NL1-4 high	13.61			< 0.0001
Bonferroni post hoc comparison	Mean difference	t value	Significant at $p < 0.05$	95% confidence interval of mean difference
NL1(+B): CD4 vs. MDGA2	0.8524	3.362	Yes	0.01736 to 1.687
NL1(–B): CD4 vs. MDGA2	0.9596	3.702	Yes	0.1059 to 1.813
NL2: CD4 vs. MDGA2	1.717	6.998	Yes	0.9088 to 2.524
NL3: CD4 vs. MDGA2	0.5741	2.304	No	-0.2463 to 1.395
NL4: CD4 vs. MDGA2	0.6755	2.762	No	-0.1299 to 1.481

Table S4. One-way Analysis of Variance of the Effect of NL(–B) ΔSite I, ΔSite II and ΔSite I+II Mutant Co-expression with MDGA1 and MDGA2 at High Plasmid Ratio. Related to Figure 6.

ANOVA	F value			p value
MDGA : NL1(–B) ΔI ΔII high	24.16			< 0.0001
Bonferroni post hoc comparison	Mean difference	t value	Significant at $p < 0.05$	95% confidence interval of mean difference
NL1(–B) WT: CD4 vs. MDGA1	2.526	8.856	Yes	1.531 to 3.520
NL1(–B) ΔI: CD4 vs. MDGA1	-0.1027	0.3800	No	-1.045 to 0.8395
NL1(–B) ΔII: CD4 vs. MDGA1	-0.5891	2.029	No	-1.601 to 0.4232
NL1(–B) ΔI+II: CD4 vs. MDGA1	-0.06621	0.2213	No	-1.109 to 0.9770
NL1(–B) WT: CD4 vs. MDGA2	2.217	7.486	Yes	1.184 to 3.250
NL1(–B) ΔI: CD4 vs. MDGA2	-0.01825	0.06645	No	-0.9757 to 0.9392
NL1(–B) ΔII: CD4 vs. MDGA2	-0.7561	2.655	No	-1.749 to 0.2369
NL1(–B) ΔI+II: CD4 vs. MDGA2	0.05128	0.1676	No	-1.015 to 1.118

Table S5. One-way Analysis of Variance of the Effect of NL3 R451C Mutant Co-expression with MDGA1 and MDGA2 at Low Plasmid Ratio. Related to Figure 7.

ANOVA	F value			p value
MDGA : NL3 low	14.78			< 0.0001
Bonferroni post hoc comparison	Mean difference	t value	Significant at $p < 0.05$	95% confidence interval of mean difference
NL3 WT: CD4 vs. MDGA1	1.077	4.845	Yes	0.3727 to 1.782
NL3 WT: CD4 vs. MDGA2	-0.03604	0.1495	No	-0.7999 to 0.7279
NL3 R451C: CD4 vs. MDGA1	0.2690	1.052	No	-0.5415 to 1.079
NL3 R451C: CD4 vs. MDGA2	0.1859	0.6730	No	-0.6895 to 1.061
